# The Role of Self‐Collection in Optimising Cervical Screening for People With Intellectual Disability: A Multi Perspective Qualitative Study

**DOI:** 10.1111/hex.70920

**Published:** 2026-10-25

**Authors:** Lauren Winkler, Rosalie Power, Kimberley R. Allison, Iva Strnadová, Allison Carter, Jane Ussher, Heather Jolly, Caroline Basckin, Sally Sweeney, Julie Loblinzk, Deborah Bateson

**Affiliations:** ^1^ Cancer Elimination Collaboration, School of Public Health the University of Sydney Sydney New South Wales Australia; ^2^ Sydney Medical School the University of Sydney Sydney New South Wales Australia; ^3^ Translational Health Research Institute Western Sydney University Sydney New South Wales Australia; ^4^ School of Education, Faculty of Arts, Design and Architecture the University of New South Wales Sydney New South Wales Australia; ^5^ Disability Innovation Institute the University of New South Wales Sydney New South Wales Australia; ^6^ The Kirby Institute The University of New South Wales Sydney New South Wales Australia; ^7^ Family Planning Australia Sydney New South Wales Australia; ^8^ School of Medicine and Public Health The University of Newcastle Sydney New South Wales Australia

**Keywords:** cervical screening, disability justice, human papillomavirus, intellectual disability, reproductive justice, self‐collection, supported decision‐making

## Abstract

**Background:**

Evidence on whether self‐collection addresses inequities in cervical screening participation for people with intellectual disability is limited. This study explored how self‐collection is understood by people with intellectual disability and those in their support and healthcare networks and factors that support or hinder access.

**Methods:**

Semi‐structured interviews were undertaken with four participant groups in New South Wales. This analysis reports on participants who discussed self‐collection (*n* = 59) within a broader interview study (*N* = 66): people with intellectual disability (*n* = 9); family members and support people (*n* = 12); managers/leaders in disability organisations and private disability services (*n* = 19) and healthcare professionals (*n* = 19). Interviews used trauma‐informed and accessible methods and were analysed using reflexive thematic analysis within a co‐production framework.

**Results:**

Three themes captured participants’ perspectives: (1) *Reclaiming bodily autonomy*: self‐collection restored decision‐making power by allowing people to collect their own vaginal sample or choose and guide a trusted healthcare professional to assist (2) *Safety, trust, and freedom from harm*: Self‐collection was perceived as less invasive than a speculum examination and supported safer participation for those with healthcare anxiety or trauma (3) *Structural inequities and ableist gatekeeping*: Limited confidence with self‐sampling, ableist assumptions, clinician‐centred pathways, and uncertainty around support roles constrained access.

**Conclusions:**

Self‐collection offers important opportunities to improve cervical screening participation for people with intellectual disability but requires multisectoral reforms including accessible sexual and reproductive health education and screening information informed by lived experience, trauma‐informed and rights‐based training across health and disability workforces and alternative screening models for safer home‐based self‐collection. These changes are essential to realise the reproductive autonomy and safety of people with intellectual disability and ensure meaningful progress towards equitable cervical cancer elimination.

**Patient or Public Contribution:**

People with intellectual disability were involved throughout the study as co‐researchers, advisors, and active research participants. An Advisory Group including two members with intellectual disability, with support from their designated support person, informed the study design, recruitment strategies, accessible methods, and interpretation of findings, with pre‐ and post‐meeting support to ensure meaningful and safe participation. The study adhered to principles of the inclusive research framework, ‘Doing Research Inclusively: Guidelines for Co‐Producing Research with People with Disability’ whereby all study materials, consent processes, and interviews used multimodal and accessible formats such as Easy Read, video, visual supports and body‐mapping and were trauma‐informed. Study findings were translated into accessible cervical screening resources by partnering with people with intellectual disability in the community, including people living in regional areas, in the planning, creation and feedback of the resources. These methods of inclusive research supported rapid translation of study findings into practical resources with potential for rapid scale‐up through Australia's National Cervical Screening Program, to support equitable access to cervical screening by people with intellectual disability.

## 1 Background

Australia is anticipated to be the first country to reach the World Health Organization (WHO) cervical cancer elimination target of fewer than four cases per 100,000 people annually, and by as early as 2035 [1, 2]. Yet equity in cervical screening remains under‐realised for people with intellectual disability. Despite comprising 1% of the world's population (> 8 million people), people with intellectual disability experience some of the lowest rates of preventive healthcare [3]. A recent meta‐analysis found that people with intellectual disability are significantly less likely to participate in cervical screening than people without intellectual disability (OR 0.30, 95% CI: 0.23–0.41), with a pooled cervical screening prevalence of 35% (95% CI: 26%–45%) [4], well below the WHO elimination target of 70% [1].

The consequences of these screening inequities experienced by people with intellectual disability on cervical cancer incidence and mortality remains poorly understood, largely due to limited population‐level data linking intellectual disability, screening participation and cancer outcomes [5]. For example, while a recent Australian retrospective cohort study in New South Wales examining cancer incidence in people with and without intellectual disability did not find a higher incidence of cervical cancer among this population, the authors highlighted several important limitations [5]. These included the likely under‐representation of people with milder intellectual disability in administrative datasets, the inability to examine outcomes by severity of intellectual disability, potential bias arising from probabilistic data linkage, and the small number of cases available for analysis of less common cancers such as cervical cancer [5].

To increase participation in under‐screened people, Australia's National Screening Program (NCSP) introduced a major policy reform in July 2022, providing universal access to a self‐collected vaginal Human Papillomavirus (HPV) test (henceforth referred to as self‐collection) for all people with a cervix aged 25 to 74 years [6, 7]. In Australia, self‐collection is offered in the primary care setting within a practitioner‐supported model [7]. This approach has important advantages as it allows appropriately trained Healthcare Professionals (HCPs) to provide patient education about screening options and supports coordination of follow‐up if HPV is detected [8].

National guidelines provide flexibility in how a self‐collected sample is taken [9]. Individuals can choose to collect their own vaginal sample, or request assistance with taking the vaginal swab by an HCP (‘assisted self‐collection’) if they cannot take the sample themselves or lack the confidence to do so [9]. National programme data [10] and implementation studies show that self‐collection improves access to, and participation in, cervical screening among under‐screened groups, including Indigenous people [11, 12], those with low socioeconomic status, and ethnically diverse communities [13, 14], who describe it as less embarrassing, more empowering, convenient and easier to do than a speculum examination [15]. However, comparable evidence is lacking for people with intellectual disability [4], particularly with regards to people's preferences for participating in self‐collection, and how screening models can be tailored to address the needs of this underserved population [16].

Improving access to self‐collection with and for people with intellectual disability requires a multisectoral approach grounded in Disability Justice and Reproductive Justice frameworks [17, 18]. Reproductive Justice, originating in the activism of women of colour, recognises that many people lack the structural conditions required to make meaningful reproductive decisions [19]. These include equitable access to abortion, contraception and safe sex and people's right to bodily autonomy [20, 21]. Disability Justice intersects with Reproductive Justice by examining how disability shapes people's access to reproductive healthcare [20]. Disability Justice foregrounds the ableist attitudes, practices and policies that have desexualised people with intellectual disability and violated their human rights by denying them the right to decide if, when, and how to have children [22]. These ongoing injustices are enacted against people with intellectual disability through forced sterilisation, exclusion from sexual and reproductive education, and the denial of contraception, fertility and pregnancy‐related care [23].

Research by Lee and colleagues shows that ableist assumptions can lead HCPs to deprioritise cervical screening for people with intellectual disability [24]. Through interviews with HCPs, these authors found that some HCPs assumed sexual inactivity and low risk of HPV infection for patients with intellectual disability without assessing risk factors such as nonconsensual sex [24]. These misassumptions, combined with challenges in communication and HCP uncertainty about whether patients understood screening and could provide informed consent, led to increased caution and delays, and in some instances, HCPs not performing screening, despite patients’ eligibility for the test [24].

Stereotypes that desexualise people with intellectual disability further disregards their heightened risk of sexual violence [25, 26], which increases exposure to HPV [27] and presents additional trauma‐related barriers to screening [4]. In Australia, 90% of women with intellectual disability have experienced sexual assault, compared with approximately 11% of women without intellectual disability [25]. Limited training in trauma‐informed practice for HCPs, including lack of attention to emotional and physical safety, and insufficient recognition of trauma histories, can further erode people's trust in HCPs and contribute to screening avoidance [28]. When these patterns persist, they do more than limit participation, they actively reproduce sexual and reproductive injustice. Within this context, self‐collection has genuine potential to advance sexual and reproductive autonomy for people with intellectual disability by restoring decision‐making power and control over one's body [29].

For people with intellectual disability, access to self‐collection also requires community awareness and knowledge of the test. A systematic review by Power et al showed a widespread absence of accessible cervical screening information for people with intellectual disability [4]. For example, a large US study interviewing over 200 eligible screeners with intellectual disability found that only a third could define cervical screening (based on Pap tests at the time of data collection), and even fewer respondents knew the recommended timing for repeating screening [30]. Without accessible information, including Easy Read, to understand the importance of HPV screening in preventing cervical cancer, screening choices and preparation needs, people with intellectual disability remain forcibly dependent on their support networks and HCPs to initiate and navigate the self‐collection pathway, creating additional access barriers [28, 31].

This qualitative study is part of a 3‐year government‐funded project (ScreenEQUAL) for achieving equitable cervical screening participation in the NCSP by people with intellectual disability [31] and was informed by an inclusive research framework; ‘Doing Research Inclusively: Guidelines for Co‐Producing Research with People with Disability’ [32]. This paper reports on interview data which were used to guide accessible resource development and aims to understand the potential for self‐collection in supporting cervical screening for this population.

### 1.1 Objective

To explore perceptions influencing the equitable participation of people with intellectual disability in self‐collection, this study addressed the following research question: How do people with intellectual disability and key stakeholders perceive the role of self‐collection in supporting cervical screening participation for people with intellectual disability?

Multisectoral perspectives were gathered from people with intellectual disability, family members and supporters, managers/leaders in disability organisations and private disability services, and HCPs, to inform systems level change. To deepen our analysis, we drew on principles from Disability Justice [22] and Reproductive Justice [17] which foreground the views, priorities, and rights of people with intellectual disability in shaping self‐collection policy and practice.

## 2 Methods

### 2.1 Study Design

This study forms part of the broader ScreenEQUAL project, a two‐stage mixed‐methods co‐production study [31]. Stage 1 comprised qualitative interviews and accessible workshops to co‐produce a suite of multi‐modal Easy Read cervical screening resources for people with intellectual disability, accompanying materials for families and support people, and trauma‐informed HCP training (2023–2024). Stage 2 involved a single‐arm trial with people with intellectual disability and associated sub‐studies with families and supporters, and HCPs, to evaluate the impact of these co‐produced study outputs (2025–2026). Findings from stage 2 will be reported separately. This analysis builds on previously published findings from Stage 1 interviews with people with intellectual disability exploring cervical screening experiences and barriers to participation [28]. Here, we examine opportunities and challenges associated with self‐collection across multiple stakeholder groups, drawing on Disability Justice and Reproductive Justice frameworks.

### 2.2 Co‐Production Study Framework and Ethics

Academic researchers worked in partnership with a researcher and Advisory Group members with intellectual disability and their support people, using accessible communications methods (e.g., Easy Read materials, Photo Symbols) to facilitate meaningful engagement throughout all stages of the research [32]. Advisory Group members with intellectual disability comprised two female self‐advocates from community‐controlled advocacy organisations in Australia. Lived experience members brought substantial experience in promoting the rights of people with intellectual disability, supporting self‐determination in healthcare, fostering community leadership, and advocating for equitable access to health services and informed decision‐making (Supporting Information: File S1). Multidisciplinary oversight was provided by an additional 10 Advisory Group members without intellectual disability from cancer and health sectors, with representation from regional and rural settings, Aboriginal and Torres Strait Islander, and LGBTQI+ communities (Supporting Information: File S1). The ScreenEQUAL Advisory Group met twice yearly, with remuneration and pre‐and‐post meeting supports offered to Advisory Group members with intellectual disability to honour their lived experience expertise, facilitate participation, and reduce potential distress.

We embedded a trauma‐informed approach throughout the project to promote safety, trust, predictability, and empowerment [33]. This included a continuous consent process whereby consent was revisited by the research team before, during, and after data collection. A detailed description of the project's accessible research methods is published elsewhere [28, 31]. Interview environments were calm and supportive, with efforts made by the research team to ensure privacy, minimise triggers and respond with empathy and validation. Participants with intellectual disability determined where they wished to engage with the research team (e.g. trusted disability service), with interviewers employing active listening, strengths‐based language, and accessible information presented in Easy Read [31] (Supplementary Information: File S2). The interviewers offered participants frequent breaks, time to reflect, and post‐interview support as needed. All interviews involving people with intellectual disability were co‐facilitated by a researcher with intellectual disability and an academic researcher, ensuring contextual sensitivity to lived experience [28]. Ethics approval was granted by the University of Sydney Human Research Ethics Committee (HREC project number: 2023/146).

This study adheres to the Consolidated Criteria for Reporting Qualitative Research (COREQ) guidelines [34], with a completed COREQ checklist provided as Supporting Information: File S3.

### 2.3 Participants and Recruitment

Participants were recruited across four groups, which included: (1) People with intellectual disability: cisgender women, transgender men and non‐binary people with a cervix, aged 25 to 74 years, living in NSW who could communicate their experience in English; (2) Support people: family members, unpaid carers and disability support workers of individuals with intellectual disability eligible for cervical screening; (3) Managers/leaders in disability organisations and private disability services and (4) HCPs: GPs, Registered Nurses and Nurse Practitioners.

People with intellectual disability were recruited exclusively from New South Wales (NSW) because their participation required in‐person interviews using trauma‐informed and accessible methods. All other participant groups were invited to participate via videoconferencing (Microsoft Teams or Zoom) or telephone, enabling national recruitment.

Interviewers invited people with intellectual disability to participate through trusted community organisations, including the Council for Intellectual Disability, Self‐Advocacy Sydney, and Women with Disabilities Australia. These organisations were identified in consultation with the Advisory Group to minimise perceived coercion and reduce power imbalances in recruitment. Participating organisations shared co‐produced accessible study information with their members, through in‐person and/or online platforms, including professional websites, social media and email distribution lists. Other participant groups were recruited through the research team's professional networks, sector mailing lists, and community contacts. The research team also used snowball sampling, which involved inviting participants to share information about the research project with their social and/or professional networks. This approach facilitated trust and safety for participants with and without intellectual disability, who may otherwise be cautious of research invitations [32].

### 2.4 Data Collection

All participants took part in a broader semi‐structured qualitative interview about cervical screening lasting between 19 and 50 min [31]. Interviews with people with intellectual disability were conducted by the chief investigator with intellectual disability (JL) and an expert disability researcher (IS), using a conversational, participant‐led format [28]. Interviews with family members and support people, managers/leaders in disability organisations and private disability services and HCPs were conducted by experienced qualitative researchers on the project, including post‐doctoral researchers (LW, RP, CB) and a senior health promotion officer (HJ).

As this manuscript focuses specifically on perceptions and experiences of self‐collection, only interviews in which self‐collection was discussed in sufficient depth to address the study aim were included. Consistent with our participant‐centred and trauma‐informed approach, interviewers did not pursue discussion of self‐collection when participants experienced the topic as confusing or uncomfortable, or where time constraints limited opportunities for further exploration. This focused analytic sample was considered appropriate for an in‐depth qualitative exploration of the research question.

Semi‐structured interview schedules were tailored to each participant group. Interviews with people with intellectual disability explored knowledge, awareness, and preferences regarding self‐collection and assisted self‐collection, and factors that support or hinder safe access to the test (Supporting Information: File S4). Interviews with family members and support people focused on experiences supporting screening participation, barriers and facilitators to access, support needs, and resource preferences. Interviews with leaders and managers from disability organisations and private disability services explored individual‐, provider‐, and system‐level influences on screening participation, opportunities for service improvement, and resource needs. Interviews with HCPs examined current screening practices related to offering and providing self‐collection and assisted self‐collection, confidence and training needs, barriers to implementation, and opportunities to improve access to and uptake of the test among people with intellectual disability. Across all groups, participants were asked about the potential role of self‐collection and assisted self‐collection for people with intellectual disability.

We conducted interviews with people with intellectual disability in person; all other interviews were conducted by the research team online using audio‐visual conferencing software (i.e., Teams or Zoom), all of which were audio‐recorded for transcription and data analysis purposes only, with participants’ consent.

### 2.5 Data Analysis

Audio recordings were transcribed verbatim by members of the research team and checked for accuracy (LW, RP, CB, HJ). We used a professional transcription service for interviews involving people with intellectual disability. During transcript checking, the researchers removed identifying information and assigned each participant a pseudonym to protect confidentiality. We used reflexive thematic analysis [33, 34] to analyse the data set in NVivo, professional qualitative data analysis software (version 14) [35], across two stages: First, the transcripts were read and re‐read to develop a comprehensive understanding of participants’ accounts of cervical screening and an initial coding framework was collaboratively developed (LW, RP, CB and HJ), with input from senior investigators (JU, IS, JL). Coding remained iterative and dialogic, with team discussions used to refine codes and interpretations.

Second, data segments relating to self‐collection were identified through both pre‐defined interview prompts and inductive coding, then collated and analysed as a focused subset (LW, RP, CB and HJ). This approach allowed our team to examine self‐collection within the broader context of screening experiences while preserving the integrity of participants’ narratives. Codes were then clustered into conceptually related groups, with movement back and forth between data extracts, codes, and candidate themes, in keeping with a recursive analytic approach [36, 37].

Themes were defined by the research team as shared patterns of meaning around a central organising concept, rather than frequency‐based categories, ensuring divergent perspectives and analytic rigour were maintained. Following extensive discussion between the CI and research team, a refined set of themes was developed and organised into a coherent Reproductive and Disability Justice analytic narrative. Quotes illustrating key points were identified and attributed to the participant's pseudonym and role descriptor (e.g., ‘Florence, parent of a person with intellectual disability’) (LW, RP, CB and HJ). The analysis foregrounded the voices of people with intellectual disability while examining systemic and interpersonal factors shaping their views and experiences.

Throughout the analysis, the research team engaged in critical reflexive practice to examine how our perspectives, disciplinary backgrounds, and social positions may have shaped the research process and interpretation of the data [36, 37]. This involved questioning dominant assumptions embedded in academic research, particularly assumptions about autonomy, health literacy, and the ease of accessing and using HPV self‐collection. We documented our positionalities, assumptions, emotional responses, and interpretive decisions through team debriefs and ongoing critical dialogue. The research team included members with diverse professional and lived experience perspectives, including disability scholarship and advocacy (IS, JL), which supported critical engagement with issues of power, representation, and knowledge production. This approach aligns with Braun and Clarke's emphasis on thoughtful and situated reflexive analysis [36, 37] and supported ongoing reflection on how our perspectives may have shaped the analytic process.

## 3 Results

### 3.1 Participant Characteristics

Nine people with a cervix with intellectual disability participated in interview questions about self‐collection (median age 43.5 years). One participant identified as Aboriginal and Australian, four identified as Australian, and four identified as other Australian (European, Asian and Middle Eastern). Seven participants self‐reported ever having been screened. Twelve family members and support people, 19 managers/leaders in disability organisations and private disability services and 19 HCPs were also interviewed. No eligible participants withdrew from the study. Demographic characteristics are presented in Table 1; HCP and disability service provider professional characteristics are presented in Tables 2 and 3, respectively.

**Table 1:** Demographic data of the participants by participant group.

	People with intellectual disability (*n* = 9)	Families and support people (*n* = 12)	Managers/leaders in disability organisations and private disability services (*n* = 19)	Healthcare professionals (*n* = 19)^a^
	*n* (%)	*n* (%)	*n* (%)	*n* (%)
Age group				
18–19	0	1 (8.3)	0	0
20–29	3 (33.3)	2 (16.7)	2 (10.5)	0
30–39	0	3 (25.0)	4 (21.1)	5 (26.3)
40–49	3 (33.3)	0	5 (26.3)	4 (21.1)
50–59	1 (11.1)	4 (33.3)	4 (21.1)	3 (15.8)
60–69	1 (11.1)	2 (16.7)	2 (10.5)	6 (42.1)
Not reported	1 (11.1)	0	2 (10.5)	1 (5.3)
**Median age** (years, range)	43.5 (27.0–65.0)	42.0 (18.0–64.0)	43.0 (20.0–67.0)	50.5 (31.0–65.0)
Gender				
Female	8 (88.9)	12 (100)	16 (84.2)	18 (94.7)
Male	0	0	3 (15.8)	1 (5.3)
Non‐binary	1 (11.1)	0	0	0
Ethnicity^b^				
Aboriginal	1 (11.1)	0	0	—
Australian	4 (44.4)	8 (66.7)	12 (63.2)	—
Asian Australian	1 (11.1)	0	0	—
European Australian	2 (22.2)	3 (25.0)	2 (10.5)	—
Middle Eastern Australian	1 (11.1)	0	2 (10.5)	—
Mixed background	0	1 (8.3%)	0	—
Not reported	0	0	3 (15.8)	—
Location,^c^^,^ d				
Major city	5 (55.6)	9 75.0	10 (52.6)	7 (36.8)
Inner regional	1 (11.1)	2 (16.7)	4 (21.1)	5 (26.3)
Outer regional	2 (22.2)	0	0	7 (36.8)
Not reported	1 (11.1)	1 (8.3)	5 (26.3)	0
Relationship status				
Single	6 (66.7)	—	—	—
Partnered	1 (11.1)	—	—	—
Married	2 (22.2)	—	—	—
Role				
Paid support person		6 (50.0)	0	0
Family member	—	6 (50.0)	0	0
GP	—	0	0	8 (42.1)
Nurse (registered nurse/nurse practitioner)	—	0	0	11 (57.9)
Disability service manager	—	0	19 (100.0)	0
Screening status (self‐report)				
Ever screened	7 (77.8)	—		—
Never screened	2 (22.2)	—	—	—

^a^ HCPs were not asked about their ethnicity and cultural background.

^b^ Participants could identify as more than one ethnic group and/or cultural group, meaning the counts are not additive.

^c^ Location refers to residential postcode identified by participants with intellectual disability, and families and support people and postcode of primary workplace for HCPs and managers/leaders in disability organisations.

^d^ Location was classified using the Australian Statistical Geography Standard (ASGS), an objective measure of road distance from populated urban centres of > 250,000 people [38].

**Table 2:** Professional characteristics for HCPs (*N* = 19).

Professional characteristics	*n* (%)
Employment type	
Disability Organisation	1 (5.3)
Private practice	7 (36.8)
Women's health service	7 (36.8)
Not disclosed	4 (21.1)
Years in practice	
Median (range)	23 (6–45)
Ever performed cervical screening for people with intellectual disability	
Yes	19 (100.0)

**Table 3:** Professional characteristics of managers/leaders in disability organisations and private disability services (*N* = 19).

Professional characteristics	*n* (%)
Workplace setting	
Private disability service	8 (42.1)
Supported accommodation	4 (21.1)
Government	1 (5.3)
Public health	2 (10.5)
Advocacy	1 (5.3)
Non‐Government (cancer) Organisation	1 (5.3)
Not reported	2 (10.5)
Organisation	
Years established	
< 5 years	3 (15.8)
5−9 years	4 (21.1)
10+ years	10 (52.6)
Not reported	2 (10.5)
Policies and procedures for supporting cervical screening	
Yes	3 (15.8)
No	11 (57.9)
Not reported/unsure	5 (26.3)
Ever had training in cervical screening for people with intellectual disability	
Yes	2 (10.5)
No	14 (73.7)
Not reported/unsure	3 (15.8)

### 3.2 Overview of Reflexive Thematic Analyses

Reflexive thematic analysis identified three interrelated themes describing how self‐collection reshapes cervical screening for people with intellectual disability within a Reproductive and Disability Justice framework. First, participants described self‐collection as enabling greater control over screening decisions, including the right to consent to, or decline, screening, to determine how screening occurs, and to decide what assistance (if any) is involved. Secondly, self‐collection was framed as creating a safer and more acceptable screening pathway than clinician‐collection of samples from the cervix using a speculum, particularly for people with previous negative or traumatic screening experiences. Thirdly, participants identified structural barriers that continue to restrict equitable access, including ableist assumptions about sexuality and capacity, inaccessible screening information, uncertainty around assisted screening of a vaginal self‐collected sample by an HCP, and GP‐mediated service pathways for a follow‐up cytology test or specialist referral. Across the dataset, the voices of people with intellectual disability anchor the analysis, while perspectives from family members, support workers, HCPs, and managers/leaders in disability organisations and private disability services highlight how power within health and support systems shapes access to self‐collection (Figure 1).

**Figure 1:**
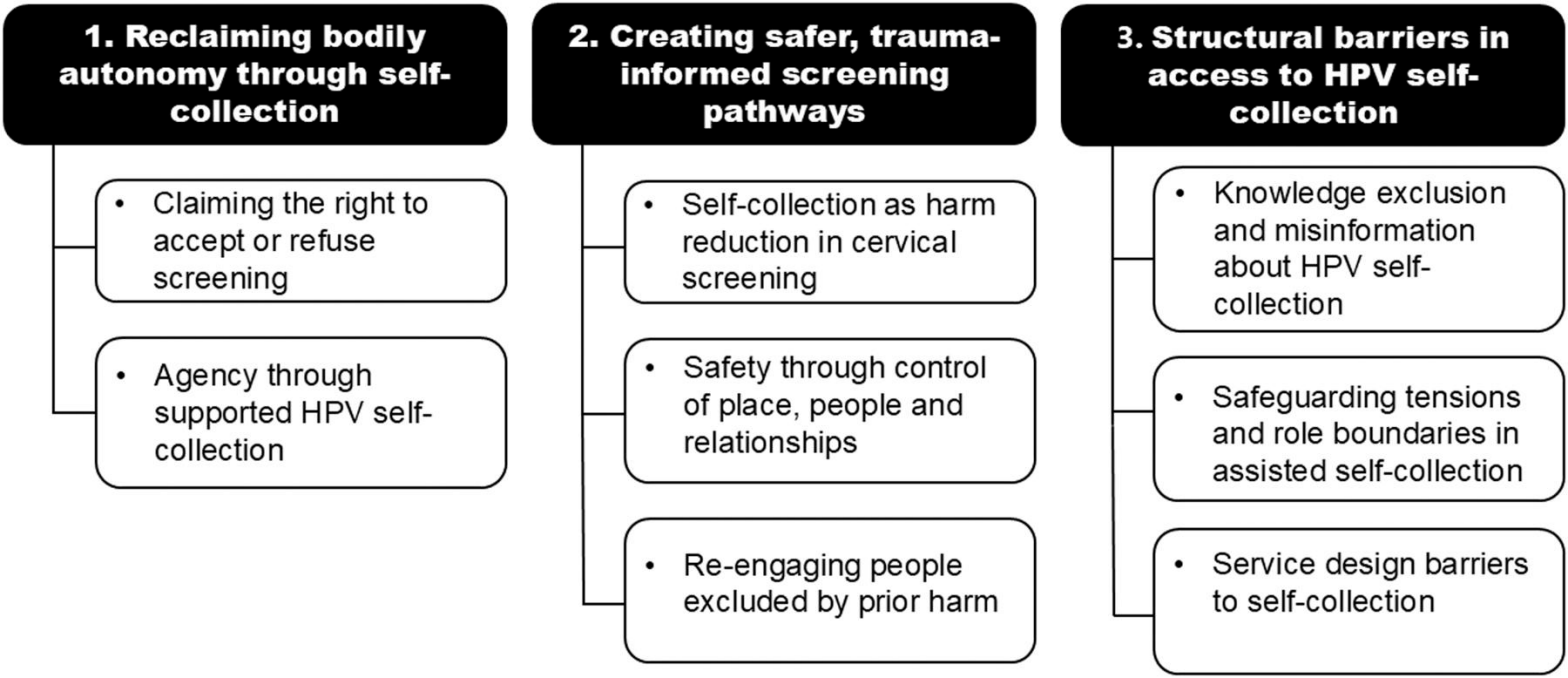
Graphic summary of reflexive thematic analysis. Overview of themes developed from the data analysis of people with intellectual disability, families and support people, managers/leaders in disability organisations and private disability services and HCPs.

### 3.3 Theme 1. ‘It's My Decision’: Reclaiming Bodily Autonomy Through Self‐Collection

Across groups, participants described self‐collection as restoring decision‐making power to people with intellectual disability. This was seen to occur by shifting screening practices, and the structural conditions in which screening is delivered, away from paternalistic control. Self‐collection promoted patient‐centred care by enabling the individual to determine whether to have the test, how it is performed, and what forms of support or assistance are involved.

Participants described autonomy not simply as the ability to perform the test independently, but as the ability to determine whether screening happened at all and what type of assistance, if any, they wanted. In this way, self‐collection was understood not simply as a form of preventive healthcare but as enabling a form of reproductive and sexual citizenship.

#### 3.3.1 ‘We Should Have the Right to Decide’: Claiming the Right to Accept or Refuse Screening

Participants with intellectual disability consistently emphasised the importance of being able to decide whether cervical screening occurred. For some, autonomy included the right to decline screening altogether. As Lexy (person with intellectual disability) explained: ‘I reckon we women should have a right to decide whether or not we should have it. I've never had mine done… Not once have I ever done it since becoming the legal age.’

HCPs similarly emphasised that supporting informed refusal was part of respectful care. Cynthia (GP) described discussing self‐collection with clients while recognising that the final decision remained theirs:

I've had a chat with a couple of my intellectual disability patients about it and in the end, they declined to have it. But that's fine. I spoke to them about it, showed the swab…at least I knew that somebody had had that chat with them about it.

Participants also framed autonomy as control over how screening occurred. Being able to choose the method of screening allowed individuals to engage with cervical screening on their own terms. Across interviews, self‐collection was therefore understood as expanding reproductive choice by allowing people with intellectual disability to determine whether screening occurred, and if so, what form it took.

#### 3.3.2 ‘Can the Doctor do it?’: Agency Through Assisted Self‐Collection

Autonomy in cervical screening was not limited to completing the test independently. Several participants with intellectual disability described preferring assistance with self‐collection of the vaginal sample, particularly when encountering the test for the first time or when they were uncertain about their anatomy. Cindy (person with intellectual disability) explained: ‘I'd have a doctor do it… Because I wouldn't know what I'm doing!’ Similarly, Salma (person with intellectual disability) felt that HCPs ‘have more experience and would know more,’ while Tanya (person with intellectual disability) reflected on her uncertainty: ‘See, I've never done it before… I don't know where I'm looking. I can't really work it out!’ Concerns about performing self‐collection incorrectly were common. Leo (person with intellectual disability) worried about ‘getting it wrong,’ explaining: ‘I don't know if I do it right, or if I muck it up.’

Managers and leaders from disability organisations reported similar anxieties among the people they supported. Irma (disability service manager) explained: ‘Even though we'd put in the language of ‘you can't get it wrong’… they weren't convinced.’ HCPs emphasised that choosing assistance could itself be an expression of agency. Frankie (nurse) described how a patient initially opted for self‐collection but later requested help:

When it came to the time to collect it, this particular woman said, ‘can you do it? Because I don't know where I'm going’ … That trust, rapport, giving time and giving choice is very important.

These accounts illustrate that autonomy in cervical screening operates along a spectrum. Participants wanted control not only over whether screening occurred but also over what forms of assistance were involved. Choosing and requesting assistance from an HCP in taking the vaginal sample due to difficulties or lack of confidence did not diminish people's autonomy, but only when the person understood their screening choices and rights (e.g. to change their mind at any time) and trusted their HCP to respect their screening decisions.

### 3.4 Theme 2. ‘Less Invasive… More Comfortable’: Creating Safer and More Trauma‐Informed Screening Pathways

All participant groups perceived self‐collection as changing the experience of cervical screening. Compared with clinician‐collection, participants described self‐collection as less invasive, more private, and more comfortable. These qualities were especially important for people who had previously avoided screening due to pain, fear, negative experiences, or trauma.

Accounts highlighted that self‐collection could create safer conditions for screening by reducing physical and emotional harm, enabling greater control over the screening environment, and reopening conversations with people who had disengaged from cervical screening.

#### 3.4.1 ‘Pap Smears Were Painful’: Self‐Collection as Harm Reduction in Cervical Screening

Many participants described clinician‐collection as painful or distressing. Leo (person with intellectual disability) explained that: ‘Pap smears were painful… That's why I wouldn't do it.’ HCPs similarly observed that speculum examinations could be confronting for some patients. Audrey (GP) noted: ‘A speculum examination is a big deal for a lot of this cohort… self‐collection has revolutionised that, in that we now have another avenue.’ By removing the need for a speculum examination, self‐collection reduced a key source of physical and emotional harm associated with cervical screening. Participants therefore framed self‐collection as a less intimidating and less harmful alternative to traditional screening procedures.

#### 3.4.2 ‘We did it at Home’: Safety Through Control of Place, People, and Relationships

Participants also emphasised the importance of feeling safe during screening. The ability to undertake self‐collection in familiar environments or with trusted people was often described as critical to engagement. For some individuals, home‐based self‐collection made screening possible in ways that clinic‐based self‐collection did not. For example, Jane (family member) explained: ‘In a GP clinic setting, it wouldn't happen… there was no way we could do it.’ HCPs described how assisted self‐collection conducted in familiar environments could support participation. Georgia (nurse) explained: ‘We did it at home… They were in their room… They had the comfort toys and things around them, and then they were quite relaxed and happy to do it.’ Control over who was present during screening was also important. Several participants expressed strong preferences regarding the gender of clinicians involved in intimate healthcare. As Lexy (person with intellectual disability) explained: ‘I would never let a man look at me down there.’ For many participants, having control over place, relationships, and the gender of care providers transformed self‐collection from an intimidating medical procedure into a manageable and dignified experience.

#### 3.4.3 ‘A Doorway Back to Screening’: Re‐Engaging People Excluded by Prior Harm

Participants perceived self‐collection as beneficial for reopening screening conversations with people with intellectual disability who had previously disengaged from cervical screening. Disability service managers noted that the availability of self‐collection created opportunities to revisit discussions that had previously been difficult; as Kamiliah noted, it provided: ‘a doorway.’ Cynthia (GP) similarly said: ‘I definitely think this is something we can discuss now… rather than it being like, ‘Oh God, how are they going to do a speculum?’ HCPs also observed that the self‐collection swab itself could change how screening was discussed. Audrey (GP) explained that when clinicians described speculum examinations, people often immediately refused screening, whereas introducing the swab allowed the conversation to continue. In this way, self‐collection was particularly helpful for HCPs re‐engaging individuals who had previously decided they would never undergo a clinician‐collected test.

### 3.5 Theme 3. ‘Hardly Anyone Knew It Existed’: Structural Barriers in Access to Self‐Collection

Despite the perceived benefits of self‐collection, participants consistently identified structural barriers that limited equitable access. These barriers operated across community, professional, and health system contexts, including low awareness of the test, assumptions that people with intellectual disability do not need cervical screening or cannot provide informed consent, unclear expectations about who could assist with the procedure, and service delivery models that relied heavily on GP‐mediated access due to limited availability of nurse‐led home visiting screening programmes. Together, these factors shaped whether self‐collection was offered, discussed, or practically accessible to people with intellectual disability.

#### 3.5.1 ‘They're not Sexually Active, so Why do They Need it?’ Knowledge Exclusion and Misinformation About Self‐Collection

Awareness of self‐collection was low across community and service settings. Cynthia, a GP who practices within a disability organisation, noted that: ‘Hardly anyone in the room was aware that a self‐collection… existed.’ Family members such as Barbara also described limited knowledge about the option: ‘I didn't even know that was possible… I don't feel I'm educated enough to give a good answer.’ Participants further described how ableist assumptions about the sexuality of people with intellectual disability could limit access to cervical screening. Hunter (disability support worker) explained that discussions often defaulted to assumptions that screening was unnecessary: ‘Most times you have to consent from the family and then… well, they're not sexually active, so why do they need it?’ HCPs observed that other clinicians were not routinely offering self‐collection in clinical practice. Dale (nurse) noted: ‘We're also finding anecdotally that a lot of doctors are not offering self‐collect.’ Misconceptions about the reliability of self‐collection also influenced uptake. Some participants compared self‐collection to COVID‐19 Rapid Antigen Tests (RATs) and assumed that a clinician‐collected sample using a speculum was more accurate compared with a self‐collected vaginal sample.

#### 3.5.2 ‘Where's the Line’: Safeguarding Tensions and Role Boundaries In Assisted Self‐Collection

Managers and leaders in disability organisations raised concerns about professional boundaries when assisting with self‐collection. Antoinette (disability service manager) explained that supporting someone with an intimate procedure could blur role expectations: ‘It muddies the relationship… If somebody who supports you can do something so intimate, then where is the line for other people?’ Participants also described situations in which HCPs expected support people to assist with self‐collection, outside of the clinical setting. Irma (disability service manager) explained: ‘The GP sent the person home… expecting the carer to help.’ For some family members, this created discomfort or safeguarding concerns. Liz (parent) reflected: ‘It opens up the expectation… that this can be done at home, perhaps by somebody else… I don't want it to be done by a support worker… It scares me.’ These accounts highlighted confusion by both disability sector workers and HCPs about who can assist in taking a vaginal sample at the request of a person who cannot, or does not feel confident to, take the sample themselves.

#### 3.5.3 ‘You have to Have a GP First’: Service Design Barriers to Self‐Collection

Participants also described structural barriers arising from the way self‐collection is delivered through existing health service models. In particular, the current requirement (in most parts of Australia) of attending a primary care clinic to receive the test was seen as limiting access for some people with intellectual disability. Cost was a recurring concern where practices did not bulk bill. Irma (disability service manager) explained that: ‘People see it as a real barrier that you have to have a GP consultation first… That's a concern for people on low income.’ For people living in rural and regional areas, the limited availability of HCPs further complicated access. Aria (disability service manager) noted that GPs in some communities ‘might be there 2 days a month,’ making it difficult to organise screening appointments. Participants also raised privacy concerns associated with seeking intimate healthcare in small communities. As Aria reflected: ‘Those communities are small… we've got to maintain that confidentiality.’ Together, these accounts highlight how system‐level features of service delivery continue to shape the accessibility of self‐collection for people with intellectual disability.

Several participants suggested that expanding the range of professionals able to provide self‐collection could improve access. Aria highlighted the role of pharmacists, noting that: ‘People know their pharmacists really well… utilising those networks is important.’ Similarly, Billy (nurse) suggested that involving community nurses ‘could reduce wait times and speed up access.’ Together, these accounts indicate that service delivery models centred primarily on GP consultations can unintentionally restrict access to self‐collection. Alternatively, participants suggested that leveraging broader community health workforces may improve the reach, timeliness, and privacy of cervical screening for people with intellectual disability, particularly in rural and regional settings.

## 4 Discussion

This study contributes new insights into how self‐collection may reshape cervical screening for people with intellectual disability. Viewed through a Reproductive and Disability Justice lens, our findings suggest that self‐collection has the potential to redistribute decision‐making power in cervical screening; however, they also reveal structural barriers that continue to limit equitable access to screening.

Participants’ accounts demonstrated that autonomy in cervical screening was not simply the ability to independently perform self‐collection; rather, consistent with disability scholarship, it operated relationally and contextually [22, 29]. For some participants with intellectual disability, autonomy meant self‐collecting their own vaginal sample; for others, choosing and guiding a trusted HCP to assist with self‐collection, or deciding not to screen. These findings challenge clinical interpretations of autonomy that equate independence with capacity [39, 40], framing self‐collection as enabling forms of reproductive agency that have historically been constrained by paternalistic approaches to sexual and reproductive healthcare for people with intellectual disability [41]. People with intellectual disability and support networks viewed self‐collection as less invasive and more acceptable than speculum‐based cervical screening but described limited knowledge and confidence with the test.

Although similar knowledge gaps are reported in the general population [42], for people with intellectual disability, these gaps reflect systemic injustices that restrict their bodily autonomy. Participants with intellectual disability often described fear of getting the test ‘wrong’ and limited familiarity with their reproductive anatomy. For some, these fears resulted in an immediate preference for HCP‐assisted self‐collection to prevent mistakes. These concerns were sometimes reinforced by family members, support people and HCPs, who felt people with intellectual disability could cause themselves physical harm, making self‐collection unsafe and risky. Albeit well‐intentioned, such responses are consistent with ableist systems that have judged, devalued, or punished behaviours associated with the person's intellectual disability and reproducing beliefs that people with intellectual disability cannot effectively manage their own bodies [21, 41].

These findings also indicate the impact of internalised ableism, whereby participants incorporated negative societal beliefs about their disability into their self‐concept, influencing expectations of their own competence and autonomy [43]. Within the context of cervical screening, participants’ concerns about ‘getting it wrong’ reflect not only limited access to tailored information and practical experience, but also the cumulative effects of messages received throughout their lives about dependence, risk, and perceived incapacity [41, 43]. Studies indicate that people's confidence with self‐collection often increases after the first attempt [44, 45, 46]. However, our findings indicate that people with intellectual disability may not be routinely afforded opportunities to practise and build confidence with self‐collection independently because of ableist assumptions about their capabilities [4, 28].

Participants’ comparisons between HPV self‐collection and self‐collection of a nasal COVID‐19 rapid antigen test (RAT), using a similar type of swab, reflect a broader shift in how self‐testing is understood by people with intellectual disability and their support networks. The COVID‐19 pandemic created a cultural precedent by normalising rapid self‐testing [47]. However, it also heightened awareness of variability in test performance, including concerns about user error and false negatives [48]. As a result, RATs may provide a salient reference point through which people with intellectual disability decide whether or not to engage in HPV self‐collection, potentially reinforcing misassumptions about test accuracy, which may impact on uptake. These findings highlight the need for clear, accessible communication for people with intellectual disability and their supporters that clarifies understanding about the equivalent accuracy of a self‐collected vaginal HPV sample compared with a clinician‐collected sample from the cervix, using a speculum [49].

Participants in this study also described common misassumptions that desexualised people with intellectual disability and denied their screening eligibility, meaning self‐collection was not routinely offered [24, 28]. This finding reflects a continued pattern of reproductive injustice for this population, highlighted by people's exclusion from mainstream reproductive education [50]. Structural reforms are therefore needed to ensure equitable access. These include government initiatives to protect reproductive education for young people with disabilities, including their sexual and reproductive anatomy [50] and to co‐produce accessible self‐collection information with screen‐eligible people with intellectual disability [29, 31]. These resources can also be used by HCPs to support understanding and accessible informed decision‐making. Trauma‐informed training for HCPs is also essential to ensure screening information and choices are presented safely [29], with attention to sexual abuse risk [51] and power differentials that can lead to acquiescence in healthcare interactions [29].

All participant groups identified easier access to home‐based self‐collection as a priority for people with intellectual disability. This preference aligns with national policy for more flexible, patient‐centred self‐collection pathways [52], with potential expansion of the programme to include nurse‐led home‐based models [53], as well as self‐collection kits, posted to peoples’ homes. Evidence from studies in First Nations communities shows that flexible models using trained non‐medical workers, such as Aboriginal community engagement workers [54] and community‐based research assistants [55], with clinical support as needed, can deliver tailored at‐home screening and achieve high self‐collection uptake in hard‐to‐reach settings (62%–81%). These approaches address limitations of the primary care pathway, including prior trauma [25], anxiety about male clinicians [28], and the potential for prohibitive HCP consultation costs [56, 57], which influenced some participants’ willingness to participate in cervical screening.

While most vaginal samples in Australia are collected in primary care, national guidelines support flexibility in collection location, including telehealth‐facilitated self‐collection, and in some settings, home‐based self‐collection with direct return of the vaginal sample to the laboratory [58]. As governments work to expand how self‐collection can be accessed for people with intellectual disability, our findings reveal important implications for policy and practice. Although some family members viewed the home environment as safe and supportive, others identified risks associated with blurred boundaries, inappropriate support worker involvement, and increased vulnerability to abuse. These concerns are heightened by higher rates of abuse in care services [26], limited knowledge about inappropriate touching [59], and lack of alternative supports as identified in this study.

These findings highlight the need for community‐led and co‐designed approaches to new service delivery models that expand access to self‐collection while avoiding unintended harm [31]. This study extends existing knowledge by demonstrating the importance of targeted disability sector training to facilitate safer access to self‐collection through clearly delineated non‐clinical roles for disability support workers and paid carers [31]. These roles include initiating screening conversations, supporting understanding of screening choices and rights using accessible information, and assisting with appointment scheduling, transport or attendance, but do not extend to assisting with taking the sample [9]. Additional mechanisms, such as the NCSP Personal Representative Function, may further support participation by enabling trusted family members or guardians to assist with administrative aspects of screening (e.g. receiving programme correspondence) [60].

Despite its perceived benefits, the potential disadvantages of self‐collection for people with intellectual disability should also be considered. In a recent qualitative study [22], Australian healthcare and disability service providers reported concerns about the design of current self‐collection swabs, which may be difficult for some people with dexterity or sensory impairments to use independently [61]. Specifically, participants highlighted that the swab and its encasing tube are particularly narrow and require fine motor skills to uncap and recap the device, as well as to effectively manipulate and insert the swab into the vagina [61]. While these concerns were not directly raised by participants in our study, future research is needed to better explore the physical accessibility of self‐collection devices among people with intellectual disability. These findings highlight the importance of considering the design and usability of the device itself, in addition to the accessibility of the screening pathways, when seeking to improve equitable cervical screening participation for and with people with intellectual disability.

Moreover, even within the context of universal access, self‐collection does not, in isolation, address inequities in cervical screening experienced by people with intellectual disability. Participants’ accounts demonstrate that progress towards cervical cancer elimination requires the dismantling of ableist assumptions and practices, ensuring people with intellectual disability are (1) recognised as sexual beings who can participate in healthcare decision‐making and (2) health systems invest in, and value, the co‐production of accessible screening information, trauma‐informed screening services, and safer, alternative screening pathways [62].

We argue that increasing the publication of co‐produced research with and for people with intellectual disability can shift community attitudes towards strengths‐based, person‐centred health systems [28]. Alongside this, multi‐sectoral workforce training in rights‐based intellectual disability health [29, 31] and inclusive public health campaigns [63] are needed to centre the voices and self‐determination of people with intellectual disability. Investment in data linkage initiatives is critical to understand whether longstanding disparities in cervical screening participation translate into inequities in cervical cancer outcomes for this population [5]. Government initiatives such as Australia's Person Level Integrated Data Asset (PLIDA) [64] offer an important opportunity to strengthen linkage between disability, screening and cancer datasets, enabling more robust monitoring of progress towards equitable cervical cancer elimination. Future linkage studies could quantify both absolute and relative inequalities in cervical cancer incidence and mortality for people with intellectual disability, providing critical evidence to guide policy priorities and resource allocation [65].

### 4.1 Strengths and Limitations

This study used an inclusive research framework enabling people with intellectual disability to contribute as co‐researchers, co‐advisors and participants. Recruitment was supported by trusted grassroots disability organisations and equivalent remuneration across participant groups, acknowledging the value and expertise of lived experience. While comprehensive recruitment strategies such as snowballing were used to reach a diverse representation of participants across groups, it remains possible that those who chose to participate had greater confidence and/or interest in participating in intellectual disability research and likely do not reflect the views of those uninterested or unsupportive of cervical screening in this population. The sample also comprised people with intellectual disability recruited from the State of New South Wales, and who could communicate verbally or through supported verbal communication, meaning the perspectives of people who use augmentative and alternative communication (AAC) or non‐verbal modes were not represented. These important gaps may limit the generalisability of the study, particularly for people who use AAC and may face additional barriers to sexual health communication.

## 5 Conclusion

Self‐collection offers a promising pathway to improving cervical participation for people with intellectual disability, yet its benefits remain unrealised. Achieving equitable access requires governments to affirm people's right to cervical screening, facilitated by coordinated and multisectoral reforms [4, 16]. Governments must provide foundational reproductive education tailored for young people with intellectual disability [50, 66], combined with Accessible co‐produced screening information [28, 31], and disability inclusive, trauma‐informed training across the health and disability workforce [31]. Cervical screening training must address ableist assumptions about sexuality and decision‐making capacity to ensure self‐collection is routinely offered by HCPs and clarifies support roles to reduce risks of abuse or re‐traumatisation. Mobilising skilled community nurses to deliver flexible, home‐based self‐collection may help to optimise pathway acceptability and safety. Together, these strategies can help translate the promise of self‐collection into meaningful reproductive justice and advance cervical cancer elimination for people with intellectual disability.

## Author Contributions

**Lauren Winkler:** writing – original draft, investigation, writing – review and editing; project administration, formal analysis, data curation. **Rosalie Power:** writing – review and editing, investigation, project administration, data curation, formal analysis. **Kimberley R Allison:** writing – review and editing, project administration, formal analysis. **Iva Strnadová:** conceptualisation, investigation, funding acquisition, writing – review and editing; methodology, formal analysis, supervision. **Allison Carter:** conceptualisation, writing – review and editing. **Jane Ussher:** conceptualisation, funding acquisition, methodology, writing – review and editing, formal analysis. **Heather Jolly:** project administration, writing – review and editing, investigation, formal analysis. **Caroline Basckin:** investigation, writing – review and editing, formal analysis, project administration. **Sally Sweeney:** conceptualisation, funding acquisition, writing – review and editing. **Julie Loblinzk:** conceptualisation, funding acquisition, methodology. **Deborah Bateson:** conceptualisation, investigation, funding acquisition, writing – review and editing, methodology, supervision.

## Conflicts of Interest

DB is a co‐PI on the implementation programme Elimination of Cervical Cancer in the Western Pacific, which has received support from the Minderoo Foundation and equipment donations from Cepheid Inc. The remaining authors declare that the research was conducted in the absence of any commercial or financial relationships that could be construed as a potential conflict of interest.

## Supporting information


Supporting File 1

Supporting File 2

Supporting File 3

Supporting File 4

Supporting File 5

## Data Availability

The data that support the findings of this study are not publicly available due to privacy and ethical restrictions relating to the sensitive nature of the information provided by participants. The data contain personally identifiable information and were collected under conditions of confidentiality. Requests for further information about the data may be directed to the corresponding author.
