# Supplementary material for: The Role of Self‐Collection in Optimising Cervical Screening for People With Intellectual Disability: A Multi Perspective Qualitative Study
Source: Health Expect. 2026 Oct 25;29(5):e70920. doi: 10.1111/hex.70920 (PMC13696019; doi:10.1111/hex.70920)
Supplement: Supplementary file 1 [file HEX-29-e70920-s004.pdf]

Easy Read

What do people with intellectual disability think about self-collection?

This booklet talks about

- how people with intellectual disability feel about cervical screening self-collection
- what people like and do not like about self-collection.

What we know

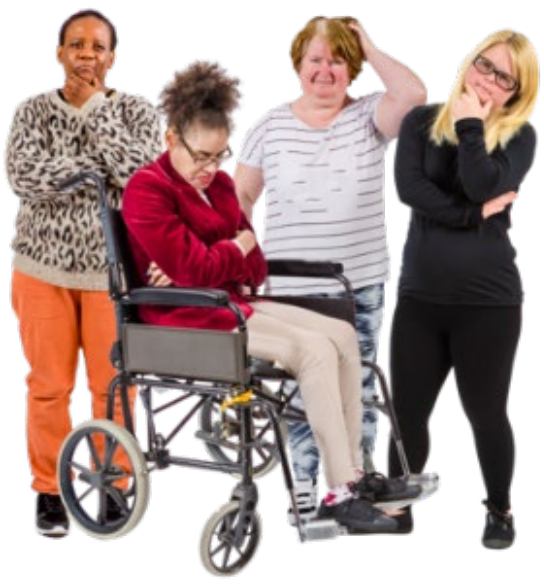

People with intellectual disability are

- less likely to have a **cervical screening test**

than people without intellectual disability.

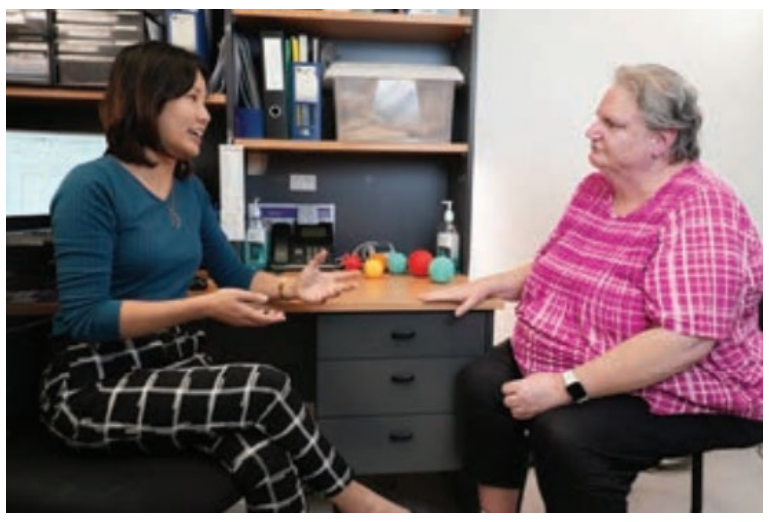

Cervical screening is a test

- to check your **cervix** is healthy.

It can protect you from having cervical cancer.

It works better than the old 'Pap Smear' test.

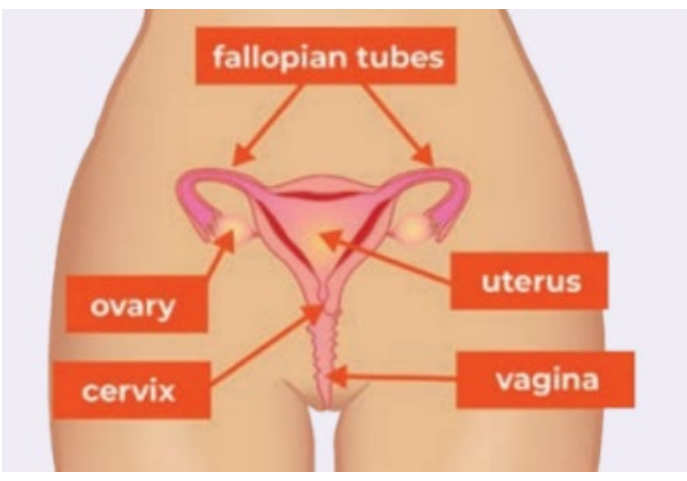

The **cervix** is inside your body

- at the top of your vagina.

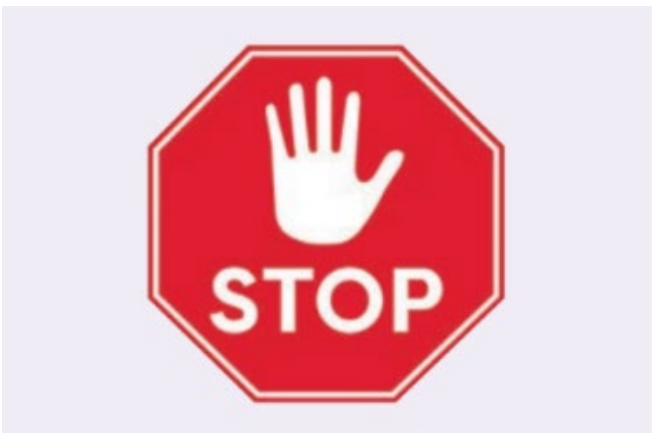

Cervical cancer is one of the easiest cancers to stop.

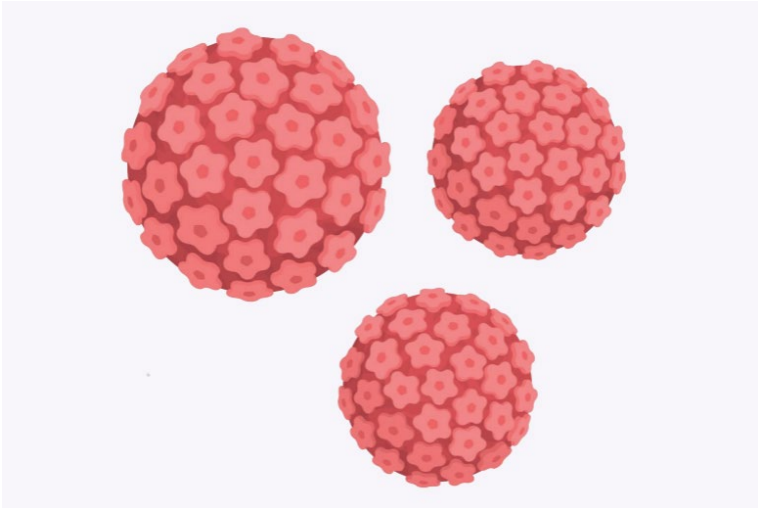

A virus called **Human Papillomavirus** can cause it.

We call it **HPV** for short.

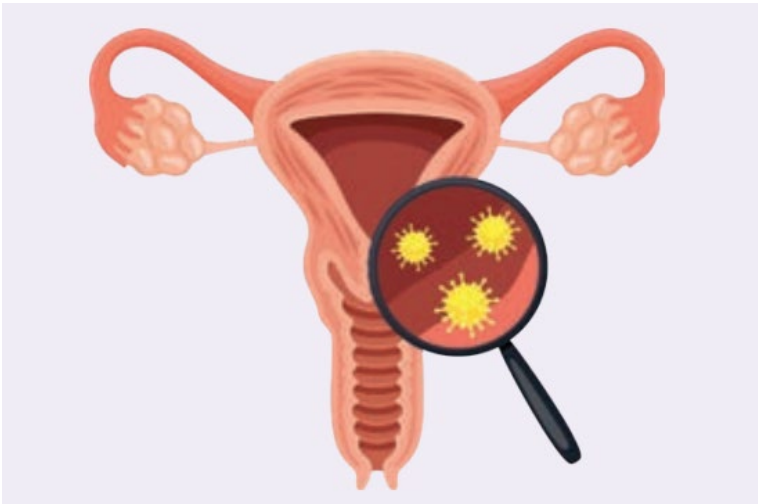

HPV can cause changes in the cervix

- that could lead to cancer.

What is self-collection?

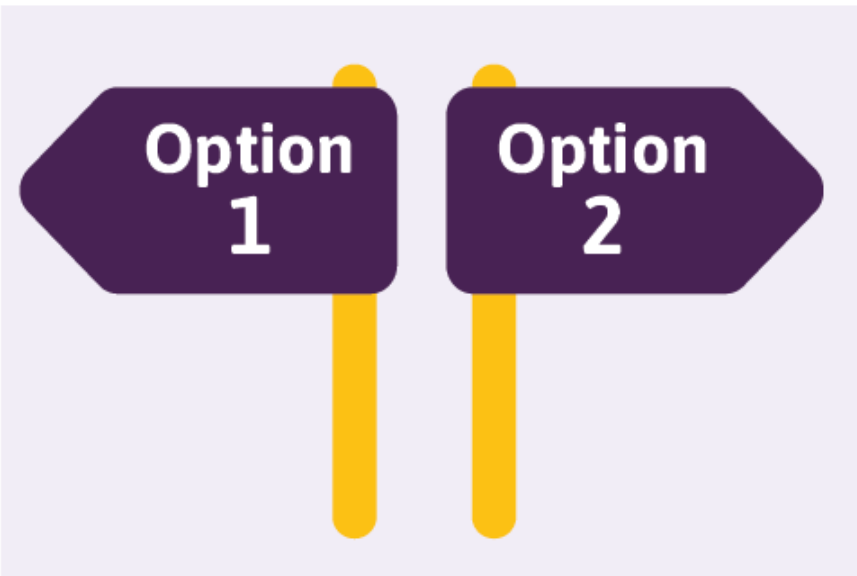

There are two ways to have the test in Australia.

Option 1 is a self-swab from the vagina.

Option 2 is a test from the cervix by a doctor or nurse.

Option 1

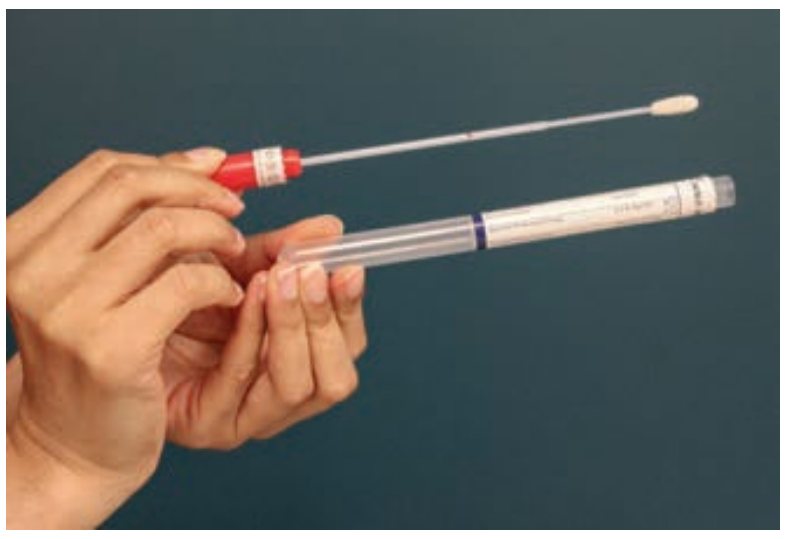

You can take the test on your own.

You put a swab into the lower part of your vagina.

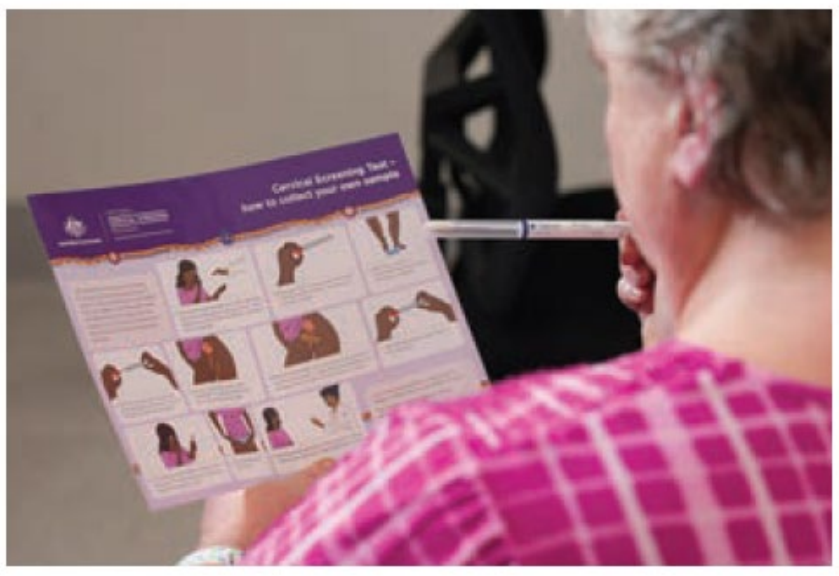

This is called **self-collection** or a self-swab.

It works as well as the test from the cervix.

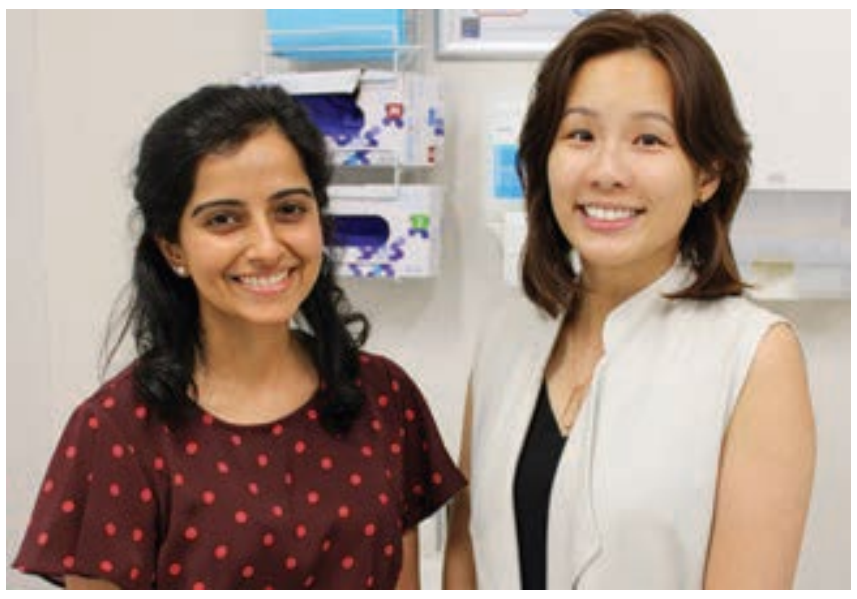

A doctor or nurse can do the swab test if you want help.

This is called **assisted self-collection.**

Option 2

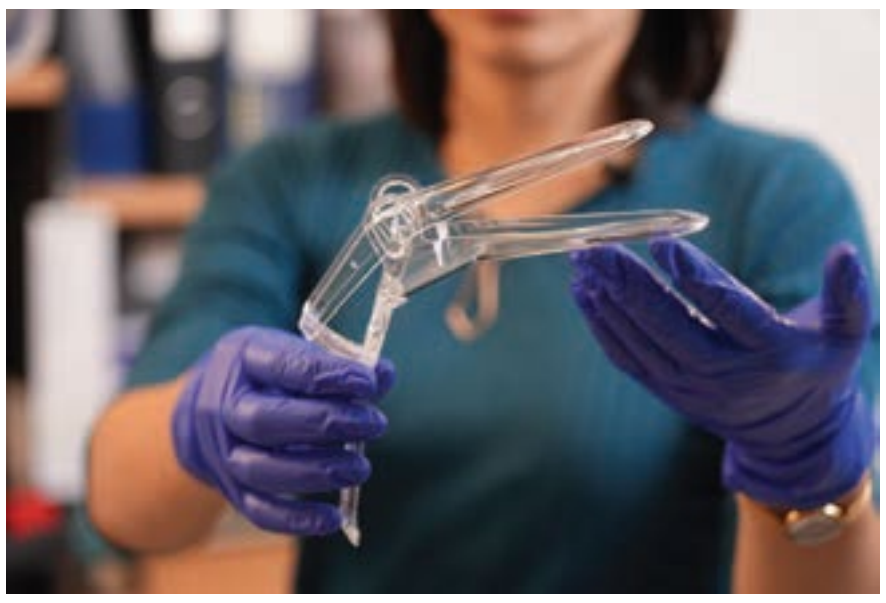

A doctor or nurse can take the test

from the cervix.

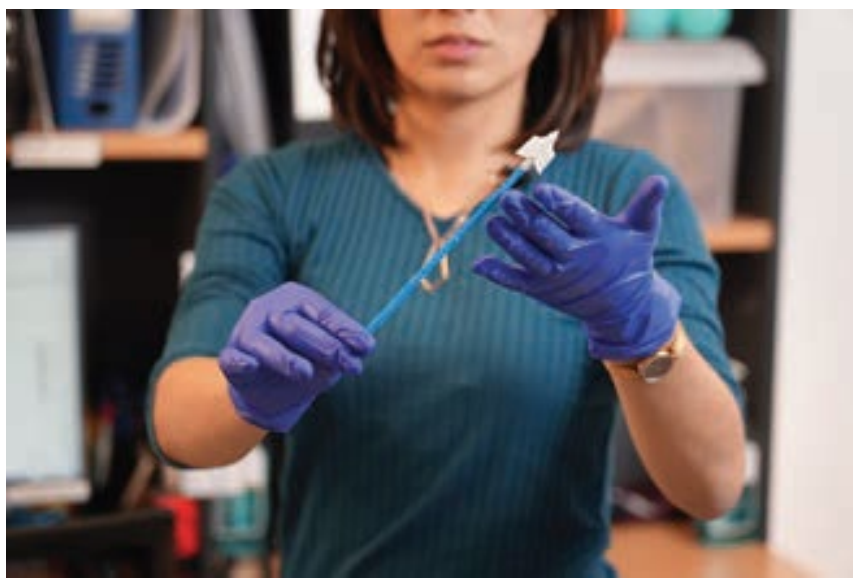

The doctor or nurse will use

- a tool called a **speculum** to see the cervix
- and a soft brush to take a sample.

Why is this study important?

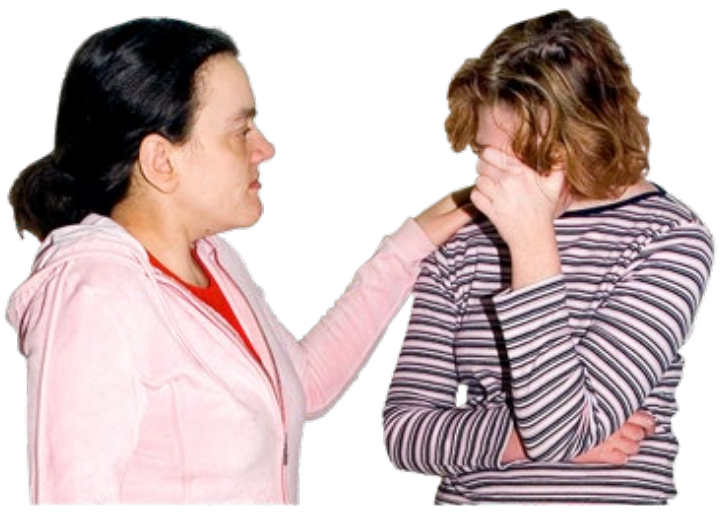

Many people with intellectual disability

- do not know about self-collection.

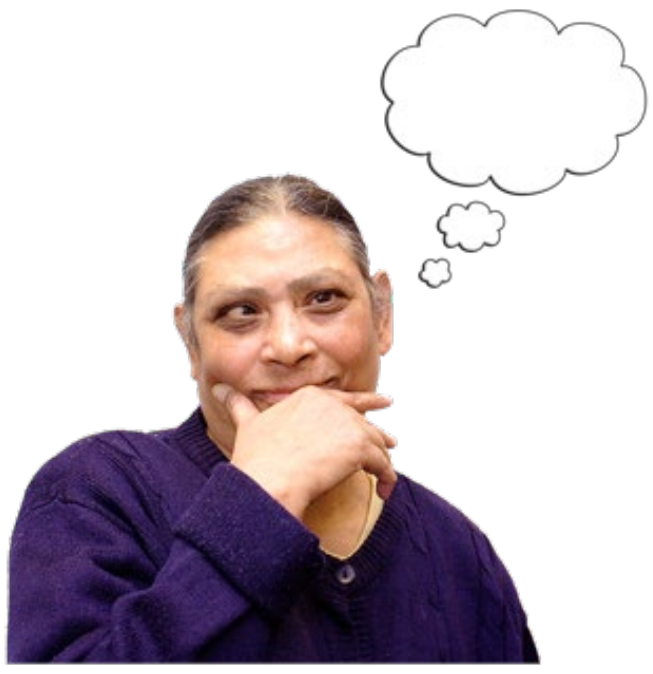

We need to find out from people with intellectual disability

- how they feel about self-collection
- what helps and does not help.

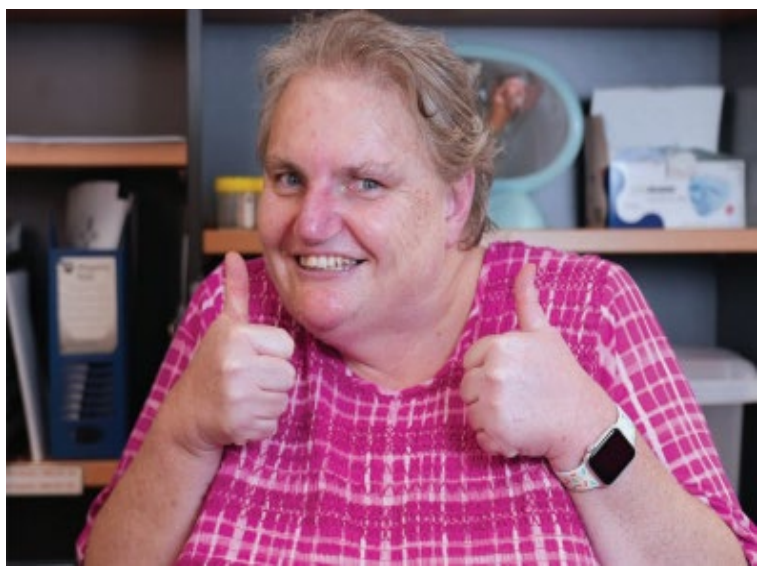

This information will help us make cervical screening

- better for people with intellectual disability.

What we did

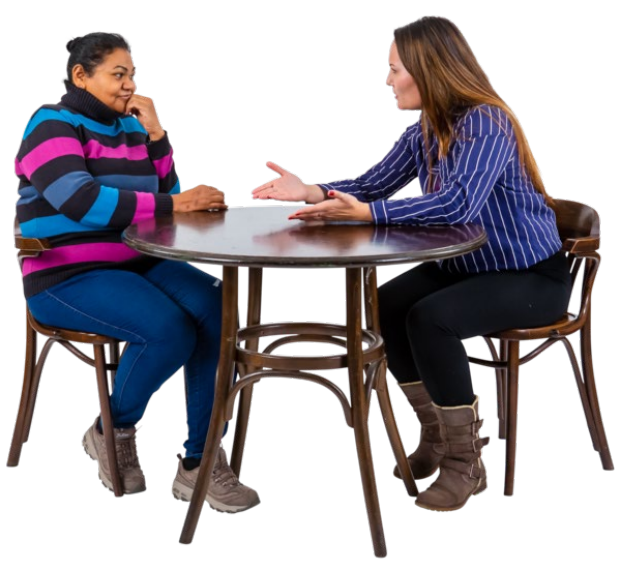

We talked to 9 people with intellectual disability about self-collection.

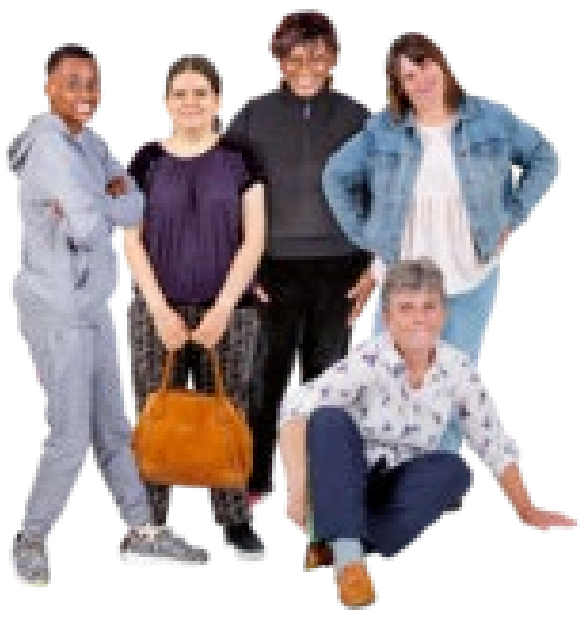

We also talked to

- 12 family members and support people
- 19 managers from disability services
- 19 health professionals.

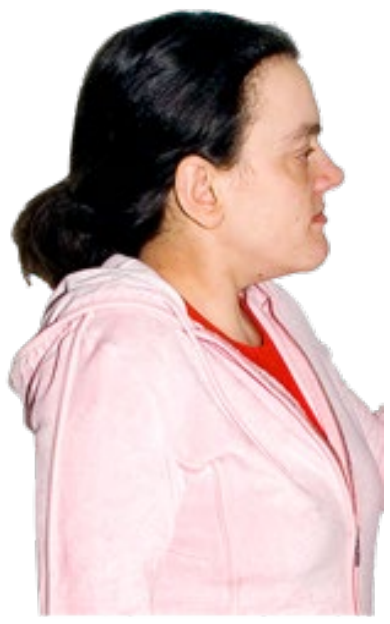

In this booklet, we use fake names for people.

What they said is in purple.

What we found out

Idea 1 – More choice and control

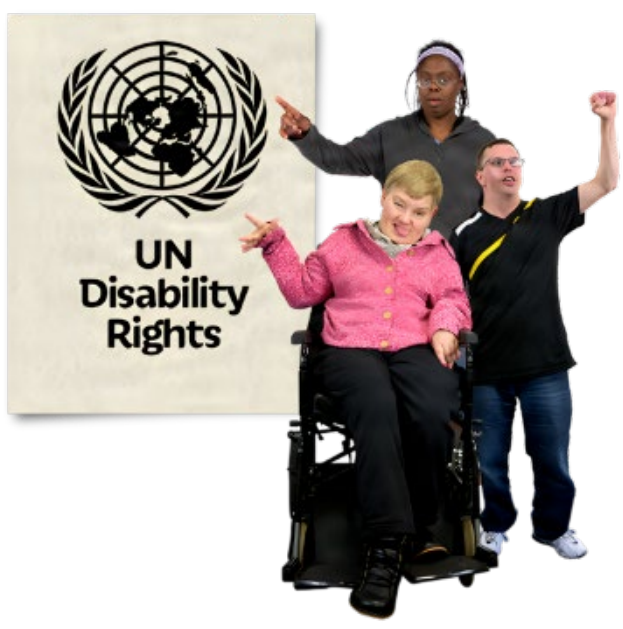

This about people with intellectual disability having the right

- to make their own decisions about self-collection.

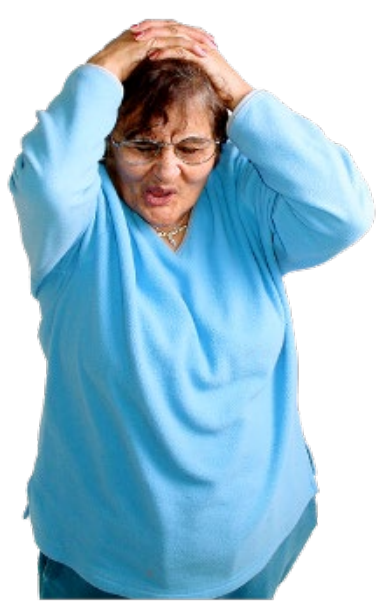

Many people liked self-collection

- because it gave them more control

over their health care.

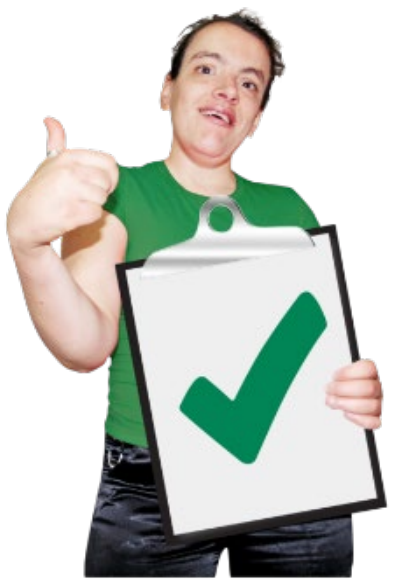

Lexy has intellectual disability.

Lexy said

Women should have a right to decide

- whether or not we should have it.

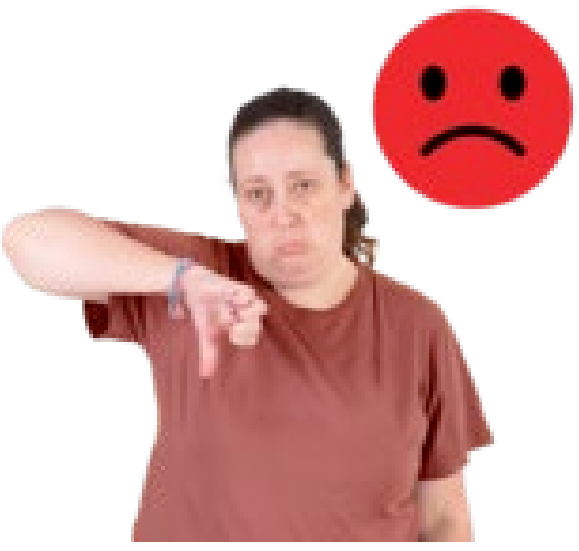

Some people with intellectual disability

- did not want the test.

That was their choice.

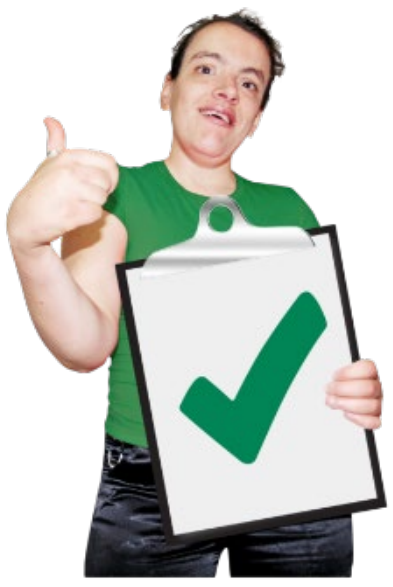

Lexy said

I have never had mine done.

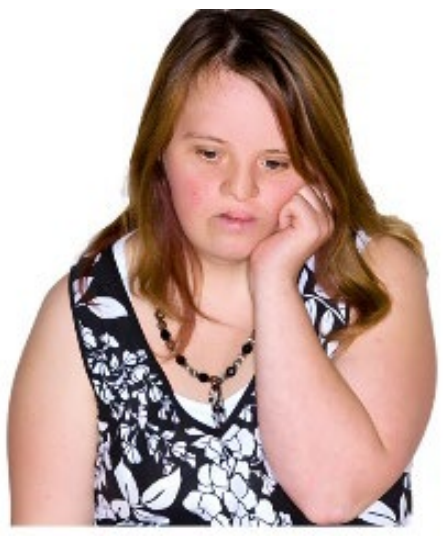

Some people with intellectual disability

- worried the test would be hard.

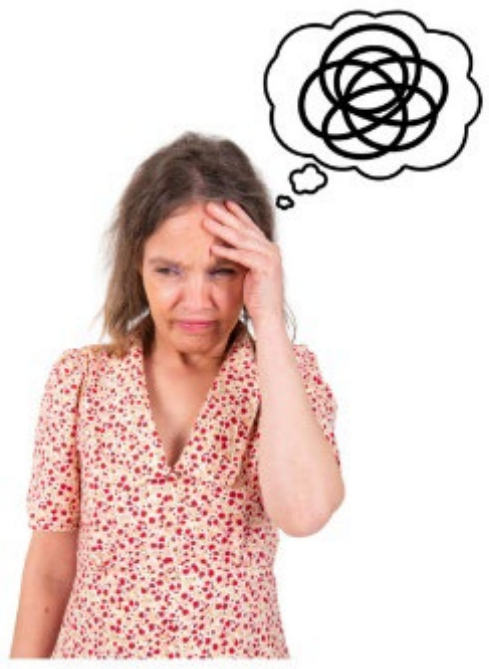

Cindy has intellectual disability.

Cindy said

I would not know what I am doing.

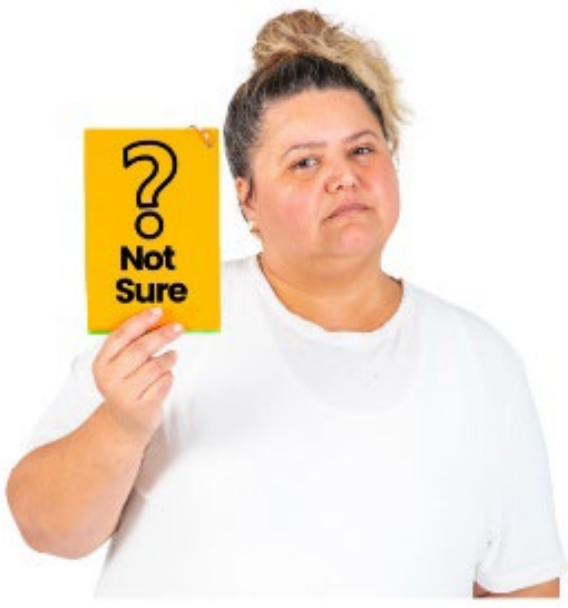

Tanya has intellectual disability.

Tanya said

I have never done it before.

I do not know where I am looking.

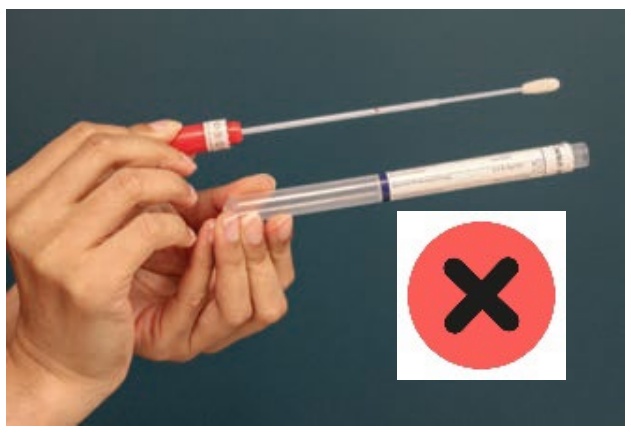

Some people with intellectual disability worried

- about making mistakes.

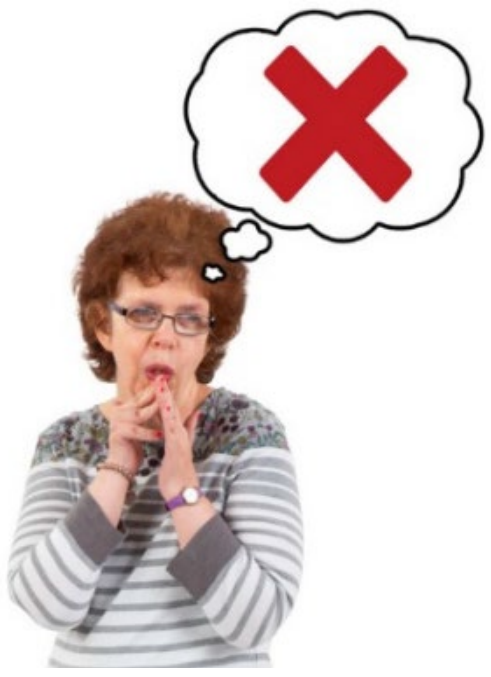

Leo has intellectual disability.

Leo said

I do not know if I do it right, or if I muck it up.

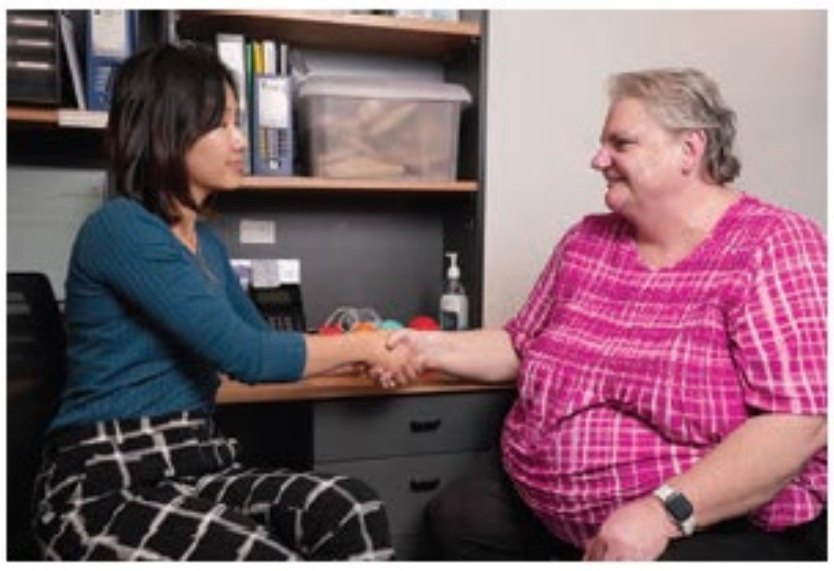

Some people liked the idea of having

- a doctor or nurse to help them.

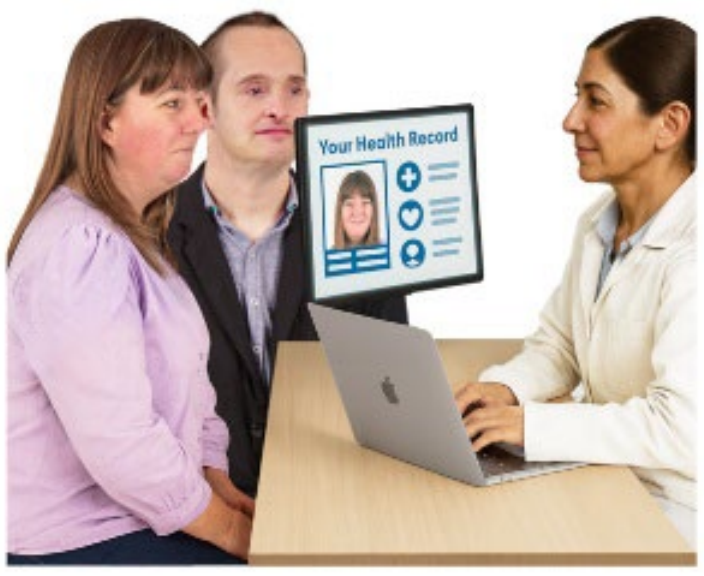

Cindy said

I would have a doctor do it.

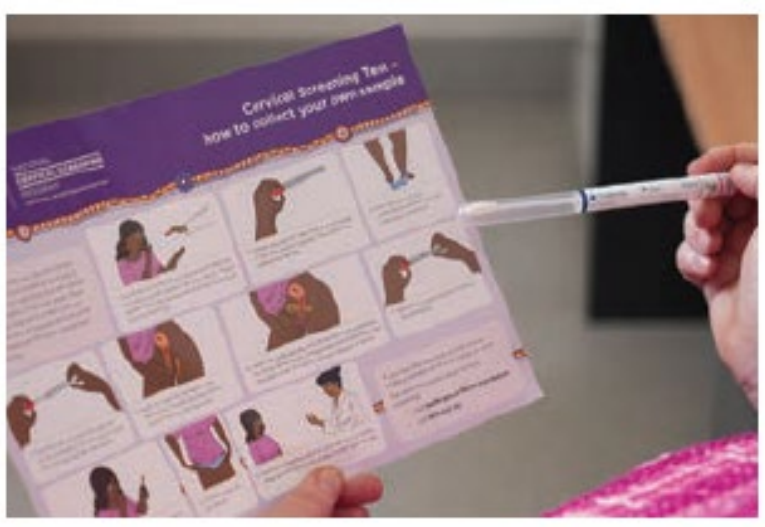

Doctors said they should offer self-collection

- to all people with intellectual disability.

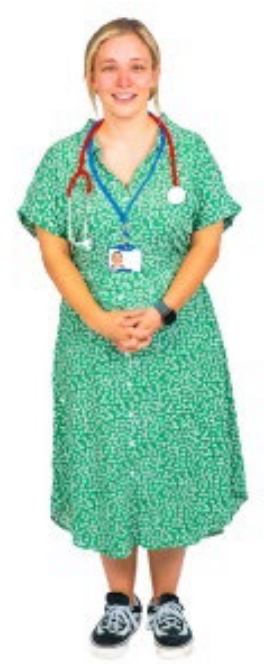

Cynthia is a doctor.

Cynthia said

I spoke to them about it, showed the swab...

…at least I knew that somebody had had

- that chat with them about it.

Idea 2 – Feeling safe and comfortable

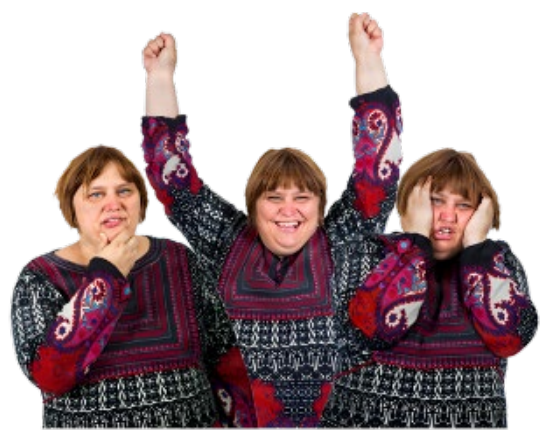

This is about how people felt about self-collection.

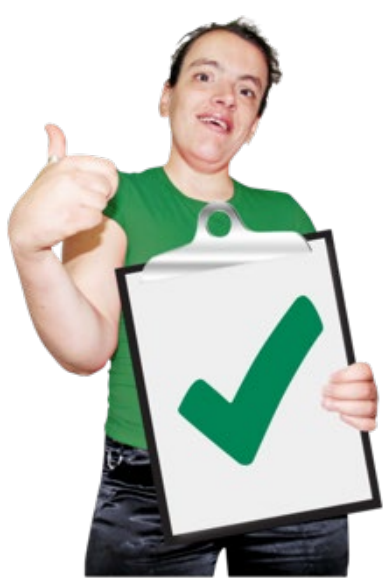

People liked self-collection because it made them feel

- safe
- respected
- in control
- comfortable.

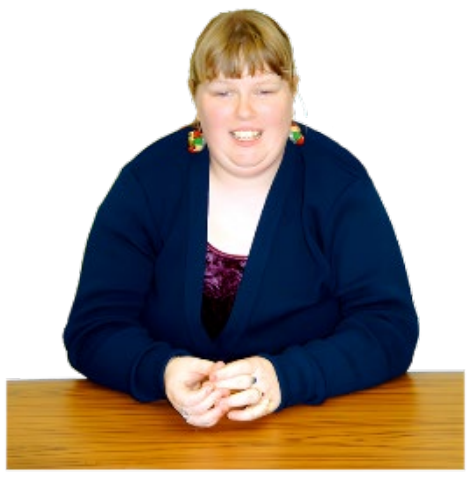

Sally has intellectual disability.

Sally said

I will feel more comfortable.

I probably won't be upset.

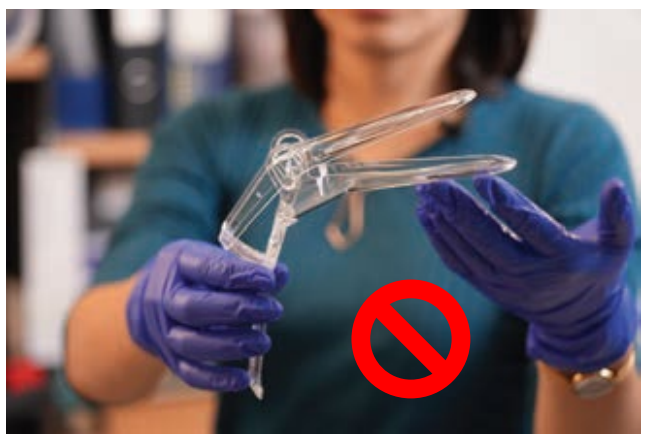

Some people liked self-collection

- because it meant they did not need a speculum.

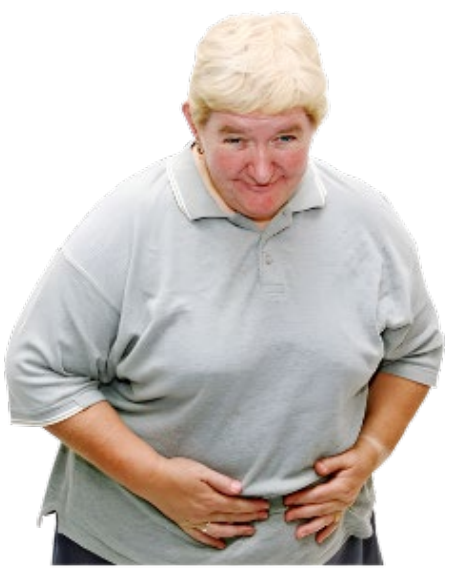

Leo found the speculum very uncomfortable.

Leo said

Pap smears were painful…

That is why I wouldn’t do it.

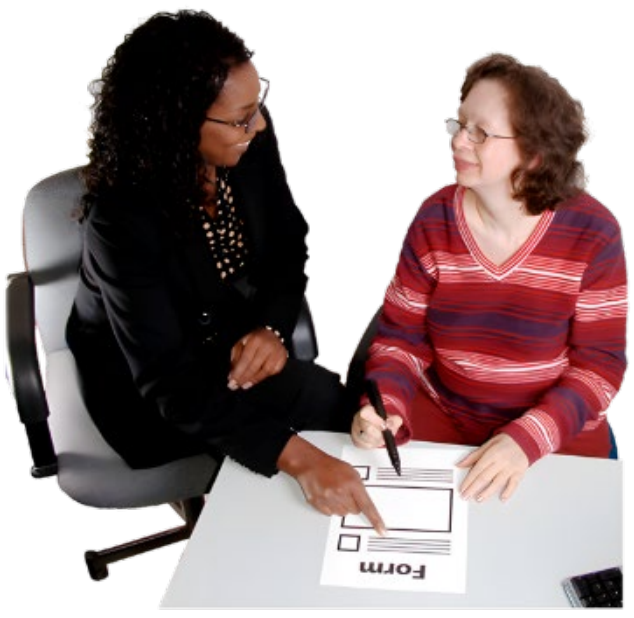

Cythina said self-collection

is something we can discuss now…

rather than it being like

Oh God, how are they going to do a speculum?

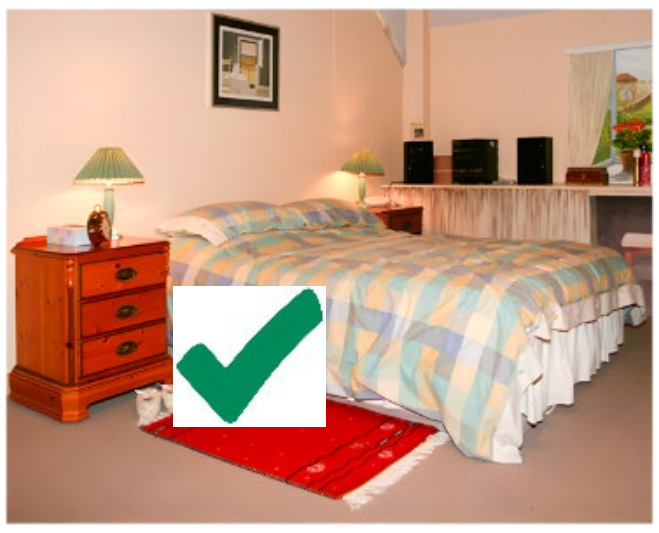

It helped when people could take the test at home

- where they felt safe

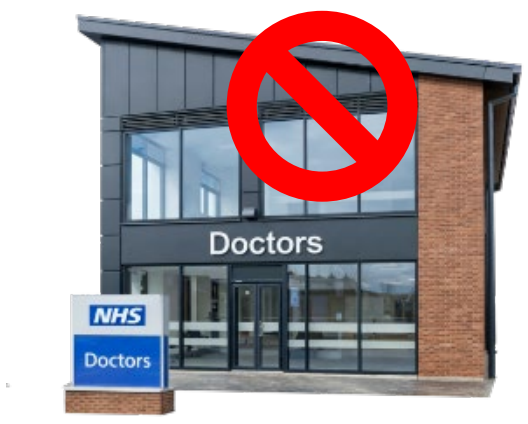

Jane is a family member.

Jane said

In a GP clinic, it would not happen.

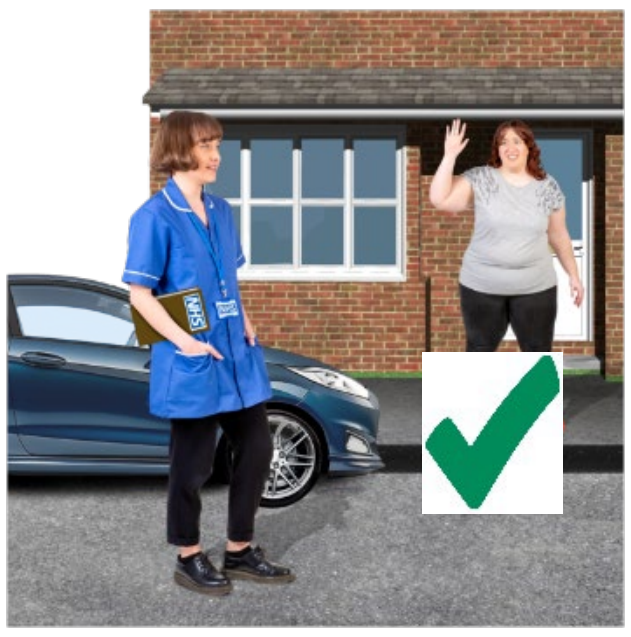

Georgia is a nurse.

Georgia helped someone with intellectual disability take the test at home.

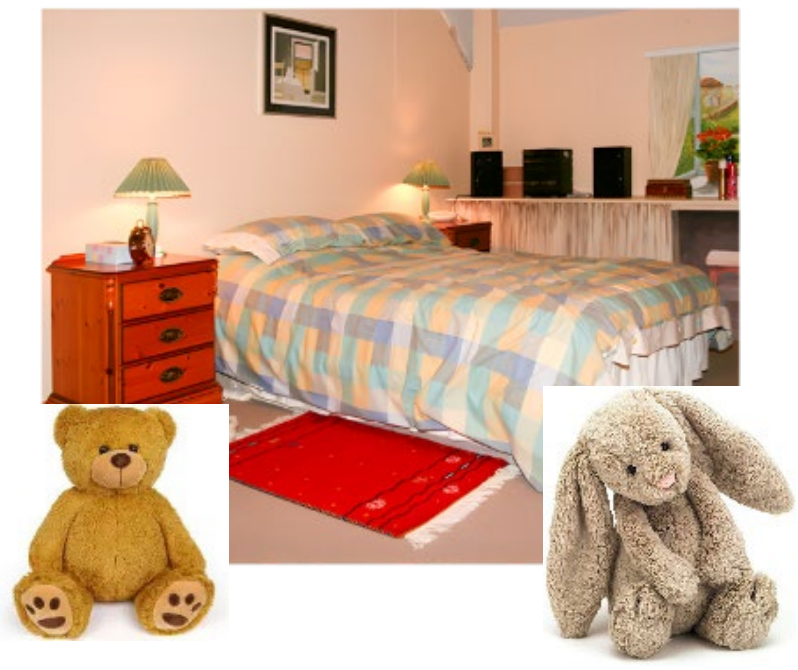

We did it at home…

They were in their room…

They had the comfort toys…

…and happy to do it.

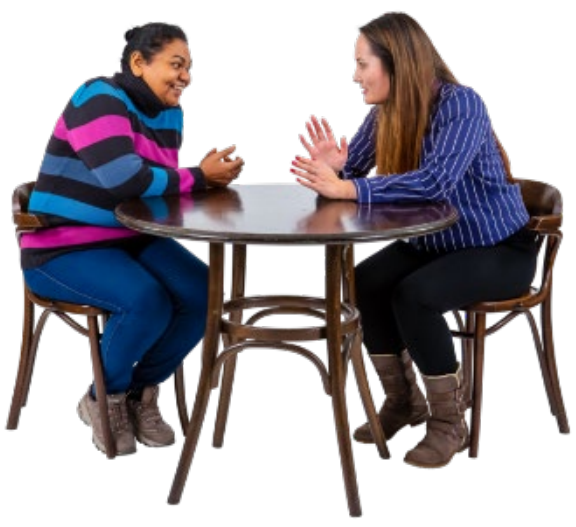

Kamiliah works at a disability organisation.

Kamiliah said about self-collection

I definitely think this is something we can discuss now.

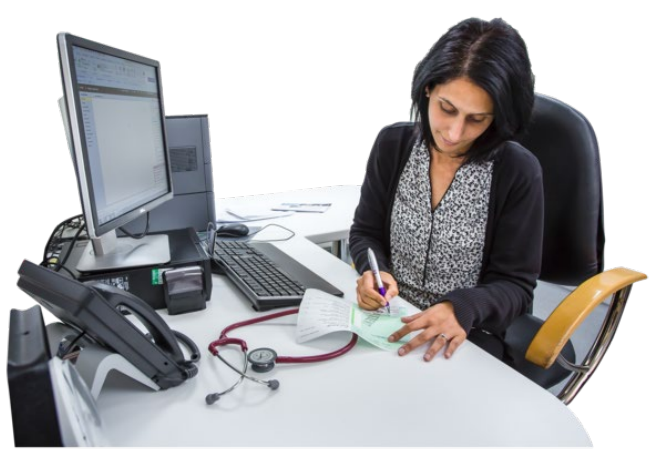

Most people with intellectual disability wanted

- a female doctor or nurse to help them with the test.

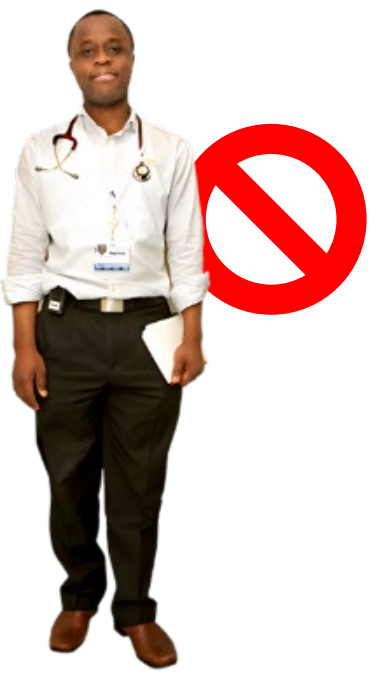

Lexy said

I would never let a man look at me down there.

Idea 4 – what makes self-collection harder

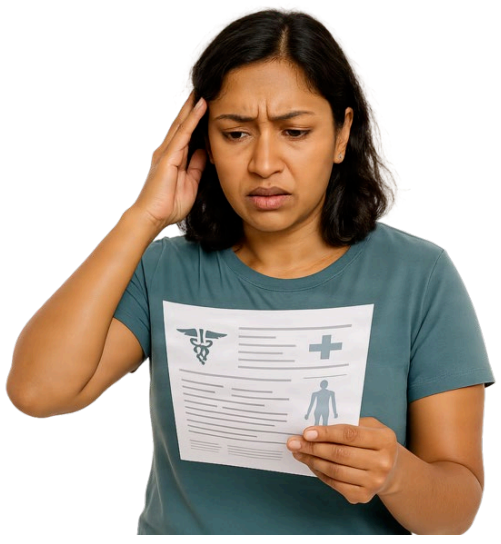

This is about what makes self-collection hard

- for people with intellectual disability.

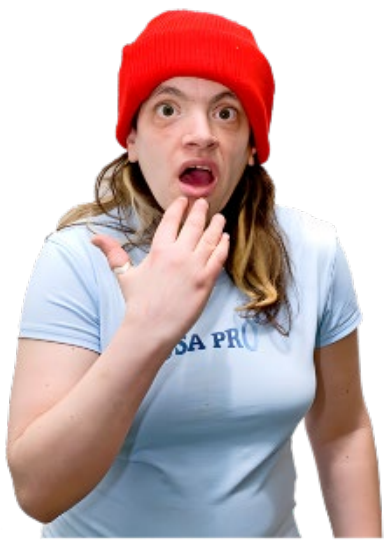

Some people did now know about self-collection.

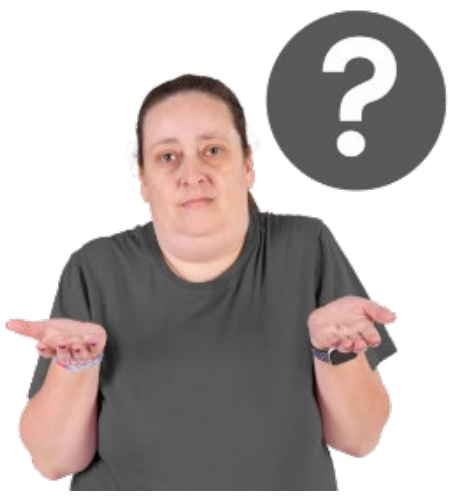

Barbara is a family member.

Barbara said

I did not even know that was possible.

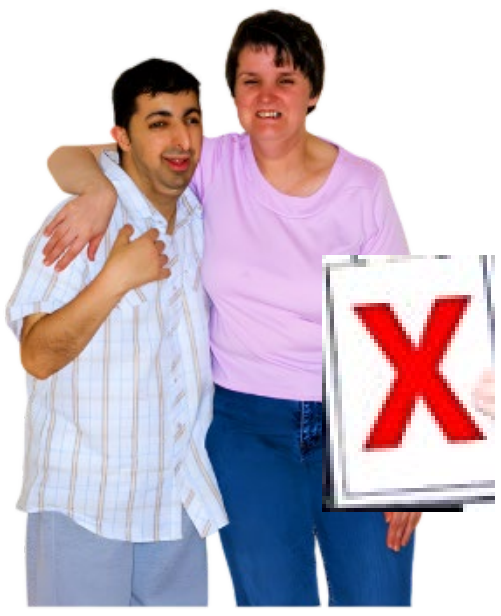

Some family members think people with intellectual disability

- do not need self-collection
- because they are not sexually active

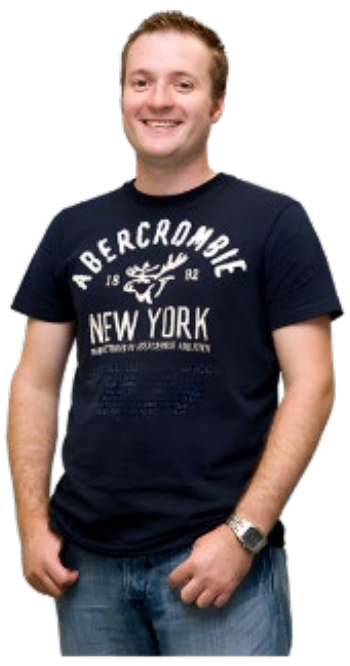

Hunter is a disability support worker.

Hunter said some families think

…they are not sexually active so why do they need it?

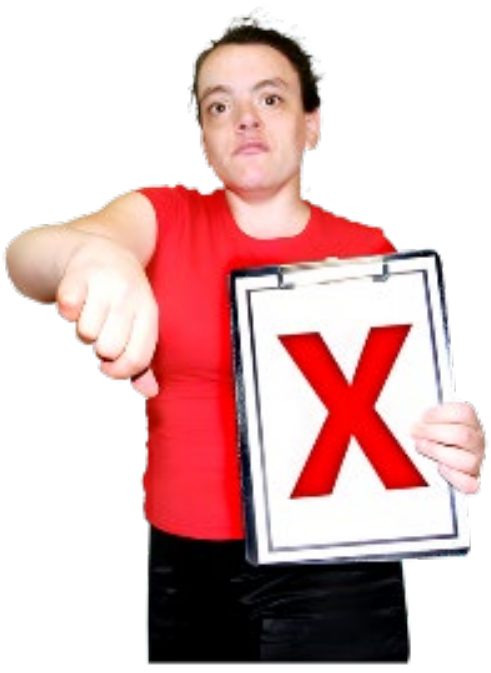

Some people are not given the choice

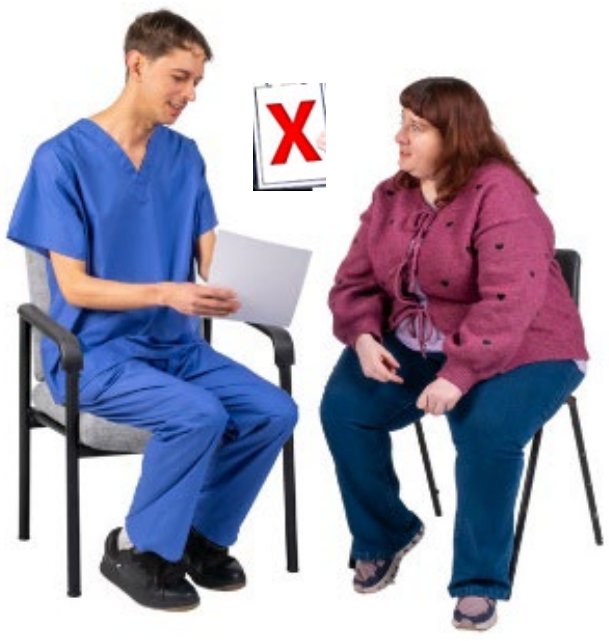

Dale is a nurse.

Dale said

A lot of doctors are not offering self-collection.

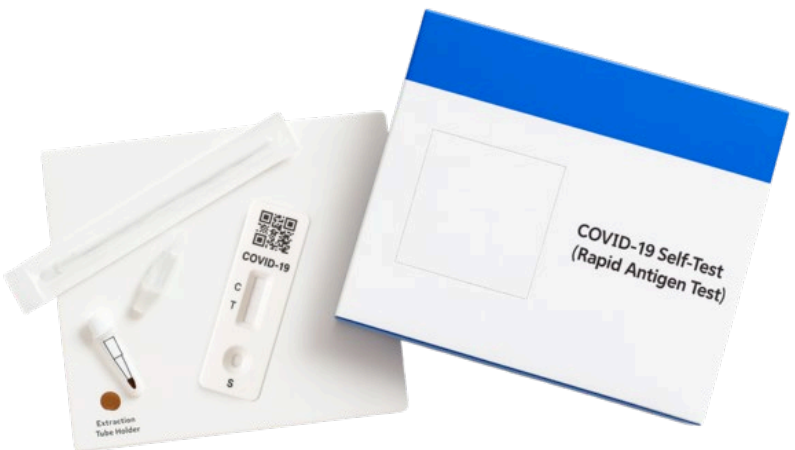

Some people talked about COVID-19.

People remembered the Rapid Antigen Test (RAT test)

- they put in their nose.

People said the RAT test was sometimes wrong.

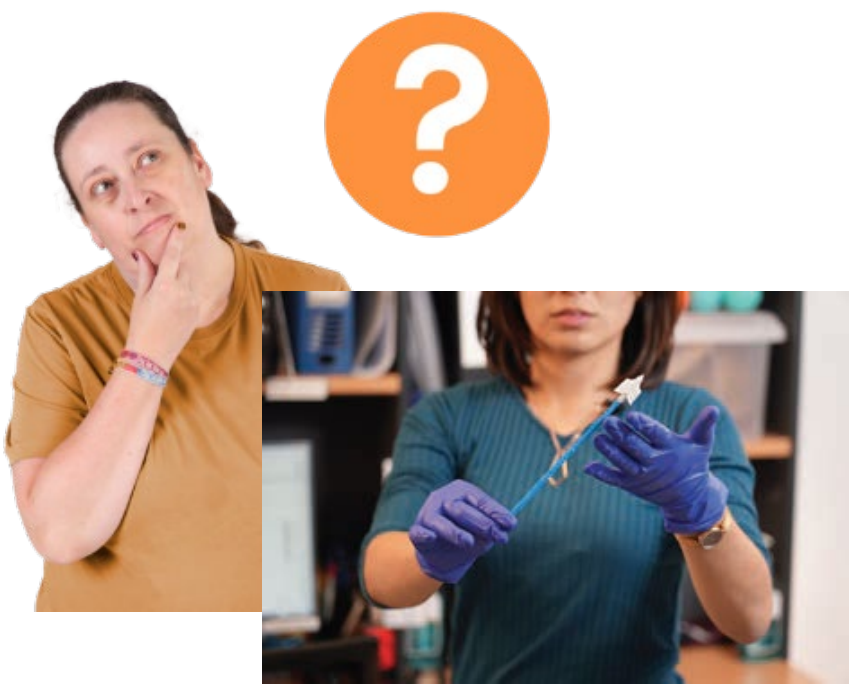

They worried that self-collection is not as good

- as a clinician-collected test from the cervix.

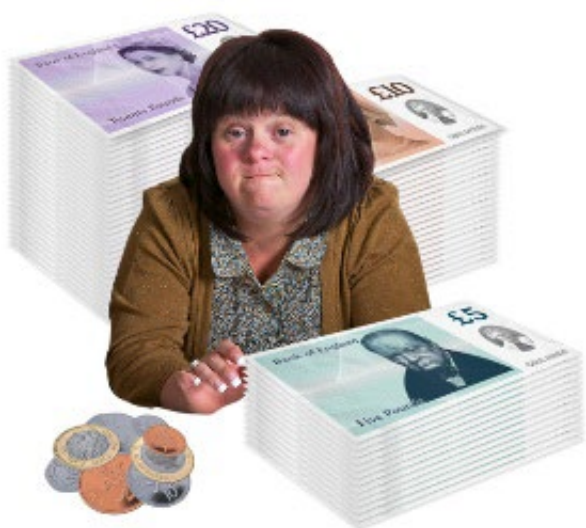

Some people worried about

- the cost of seeing a doctor or nurse.

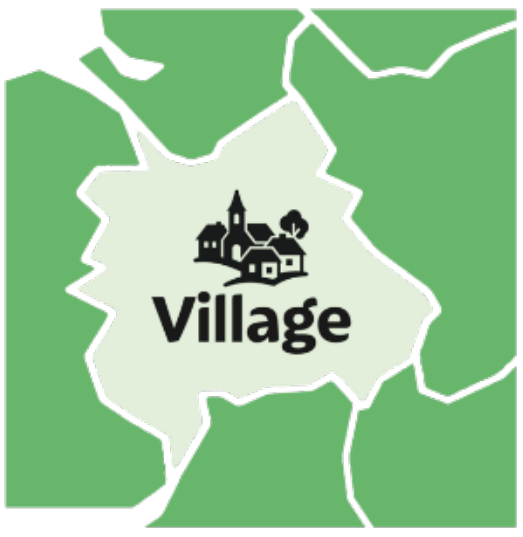

Managers at disability services said

- there are not enough doctors and nurses for people with intellectual disability
- who live in regional areas

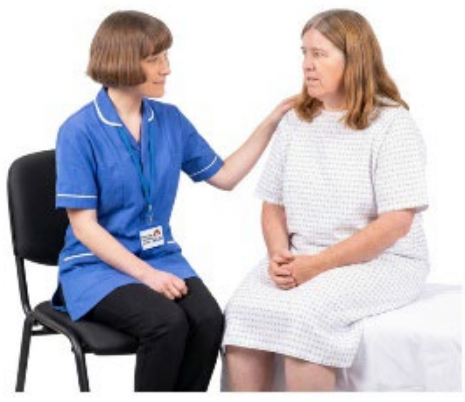

This can make it hard to find

- a doctor or nurse they trust.

Idea 3 – Systems

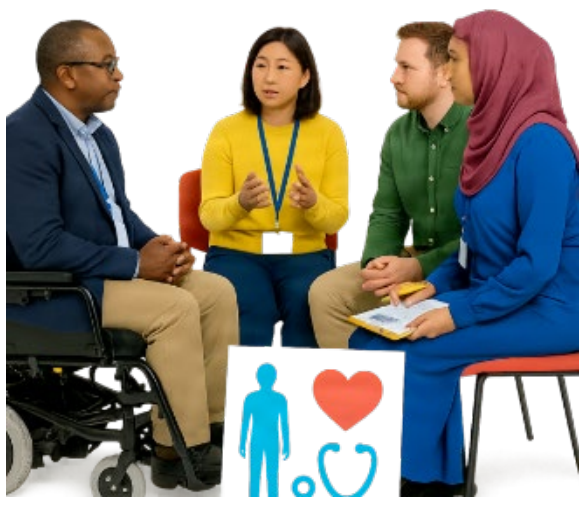

This is about how health care services

- can support people with intellectual disability.

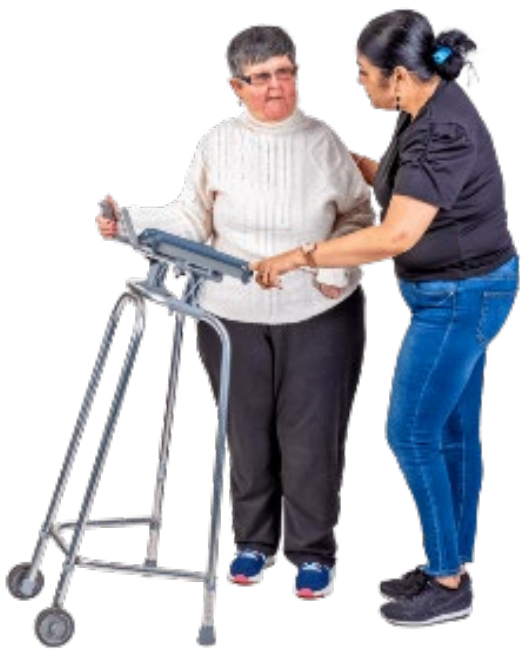

Family members and support person said self-collection is private.

Only a doctor or nurse or trusted family member

- should help with the swab.

Support workers must not help with the swab.

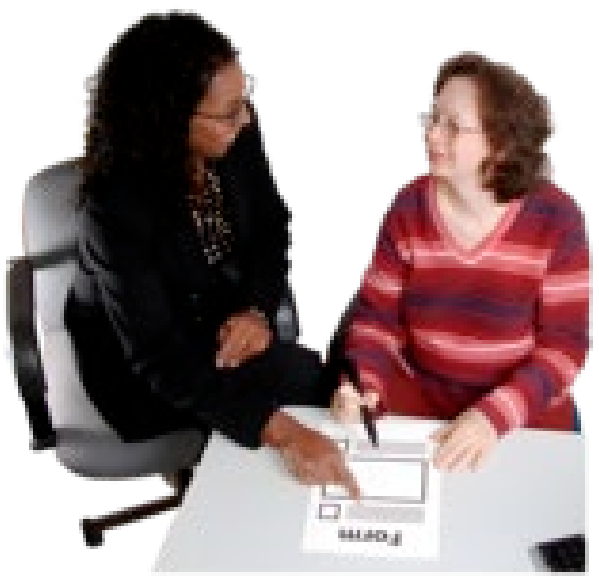

Liz is a family member said

I do not want it to be done by a support worker…

It scares me.

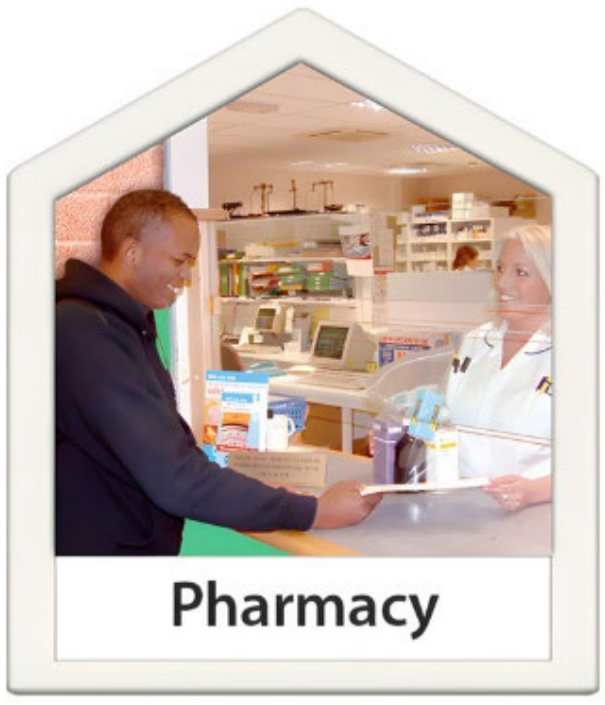

Some people thought it would be helpful

- to collect a self-collection swab from the pharmacy.

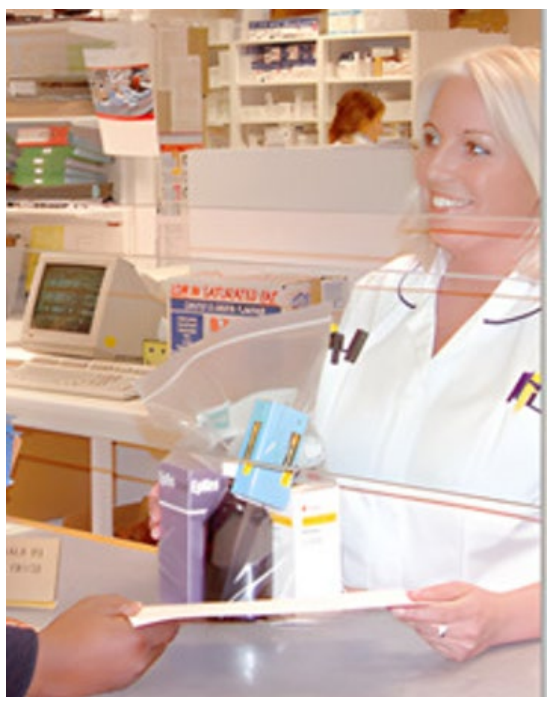

Aria works at a disability service.

Aria said

People know their pharmacists really well.

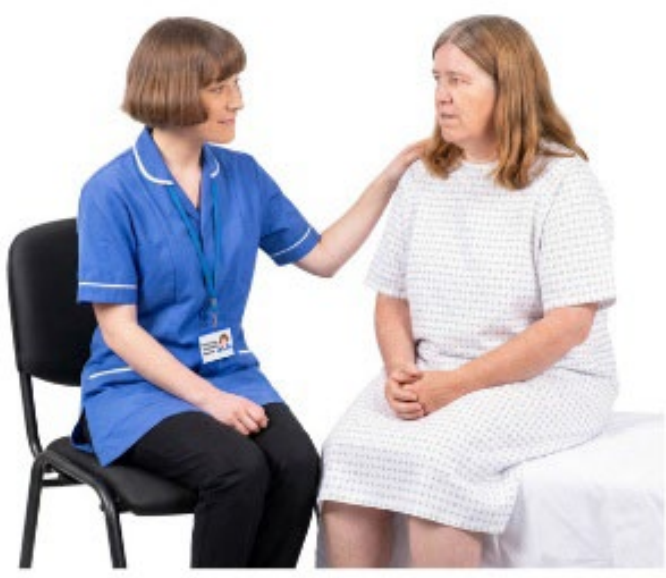

Billy is a nurse.

Billy said

nurses could reduce wait times and speed up access.

How to make things better

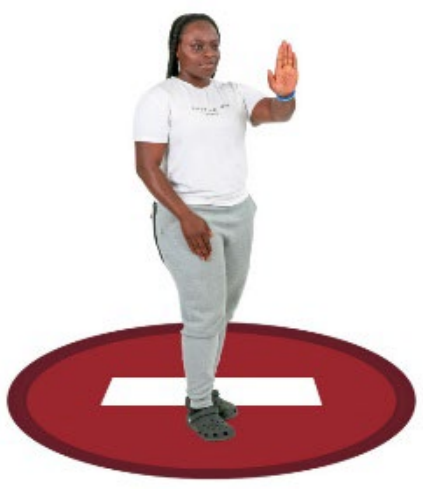

This study found many barriers that make self-collection hard

- for people with intellectual disability.

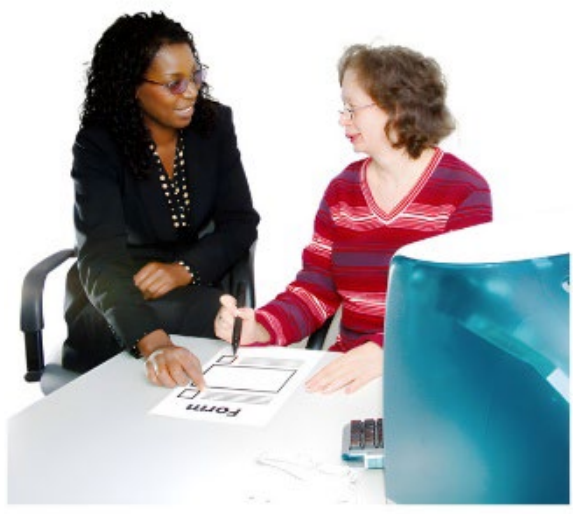

Health care workers need to see people with intellectual disability

- as experts in their own care
- who learn information in different ways.

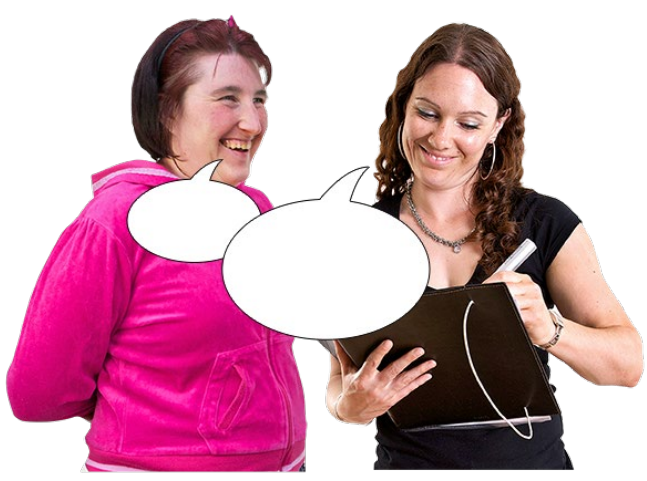

Health professionals need to give people

- the same rights and choices
- as everyone else

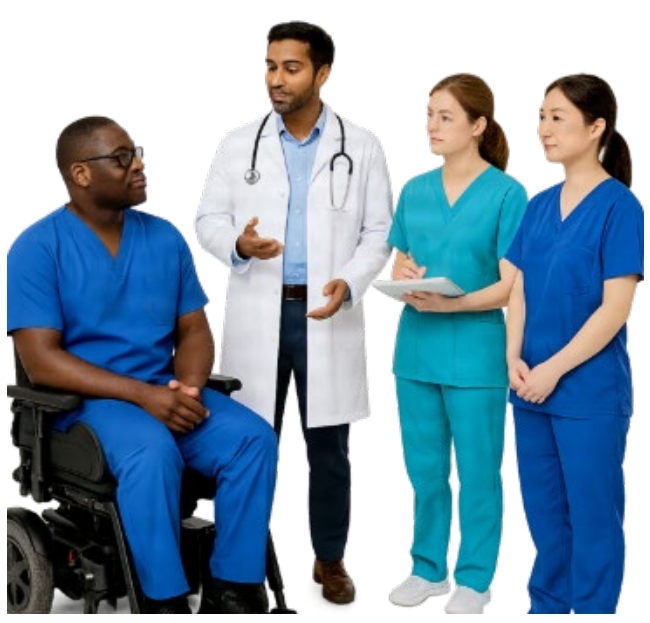

Health professionals need training

- so they can talk about self-collection safely.

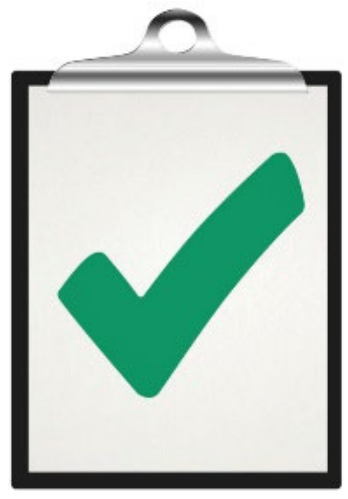

People should have the choice to

- take the test themselves
- ask the doctor or nurse to help
- or not have the test

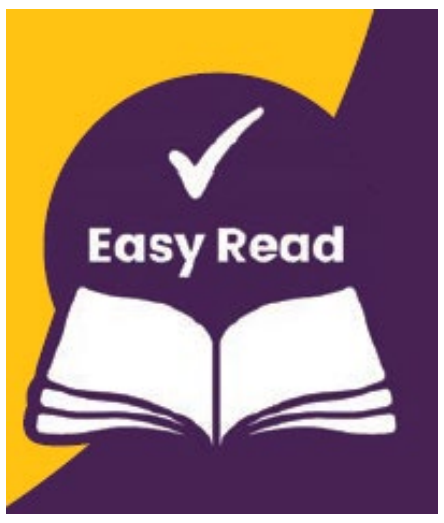

People with intellectual disability need

- Easy Read information about self-collection.

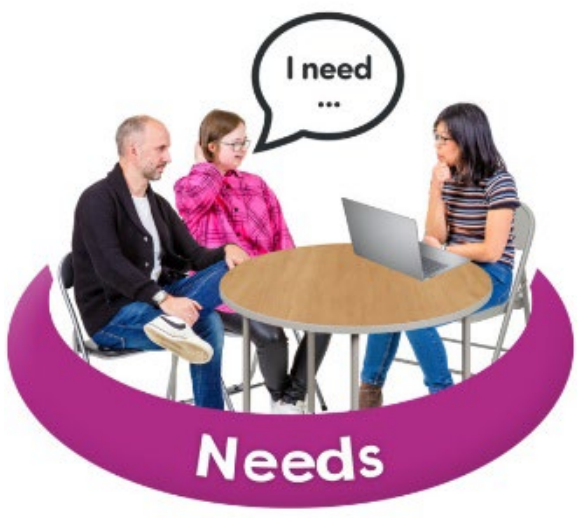

People with intellectual disability wanted

- choices about where they can do the test.

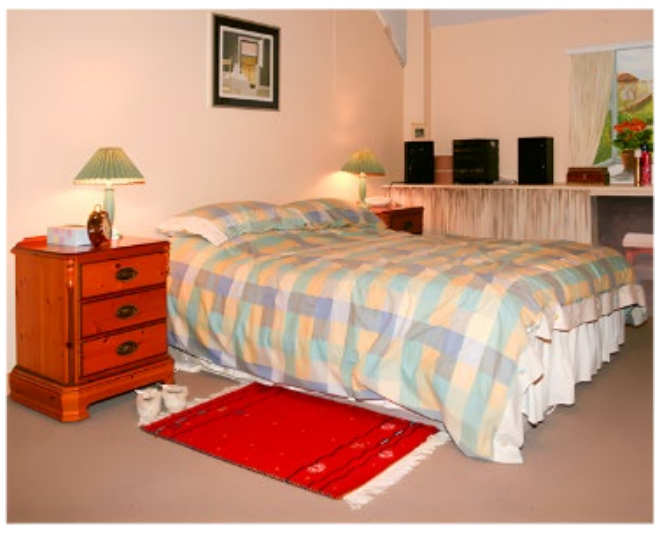

Some people with intellectual disability wanted

- to do the test at home.

More information

The ScreenEQUAL team made this booklet.

It was made with Photosymbols.

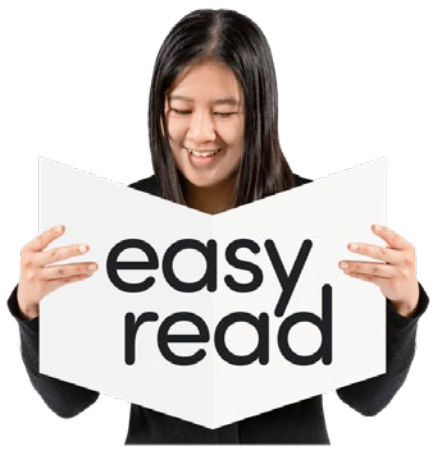

This booklet is an Easy Read summary about

- some of our research.

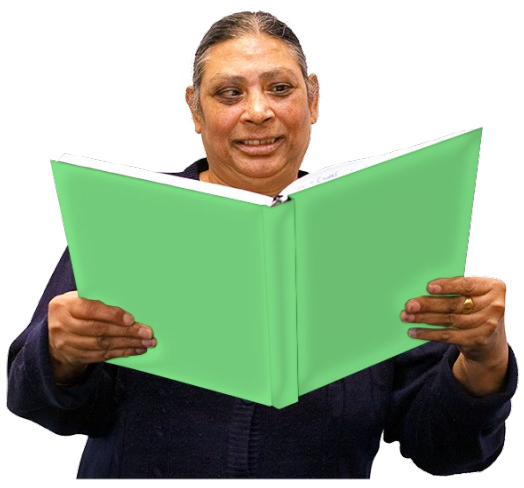

The full article is called:

- The role of self-collection in optimising cervical screening for people with intellectual disability: A multi perspective qualitative study

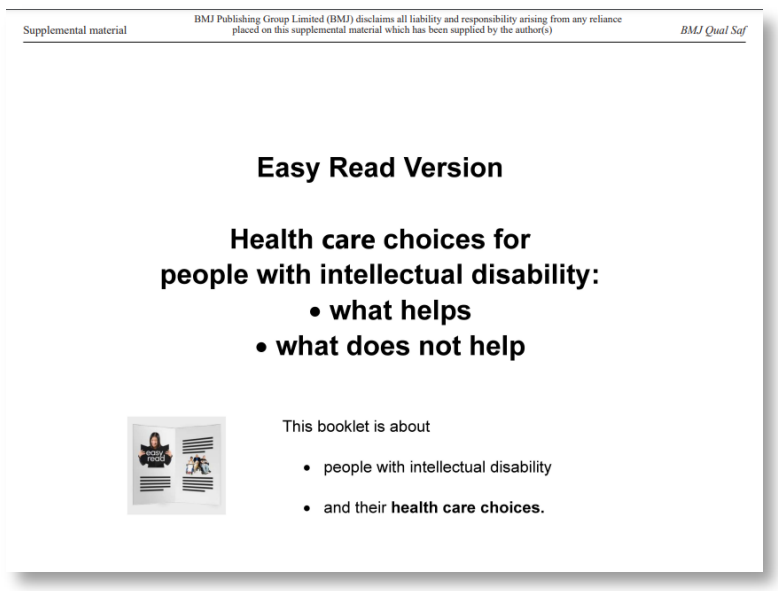

To make this booklet we looked at other Easy Read research:

- Health care choices for people with intellectual disability

  https://doi.org/10.1136/bmjqs-2023-016113

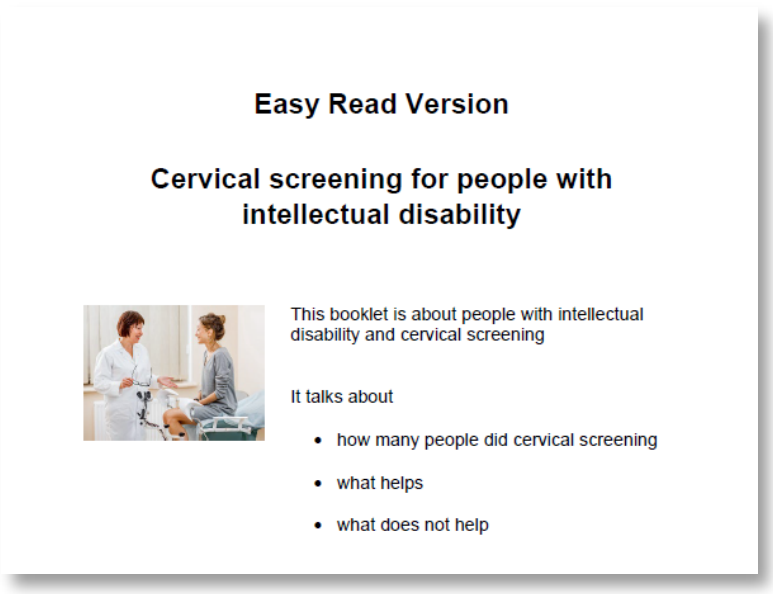

- Cervical screening for people with intellectual disability

  https://doi.org/10.3389/fpsyt.2024.1379497

Suggested citation

Winkler L, Power R, Allison KR, Strnadová I, Carter A, Ussher J, Jolly H, Basckin C, Sweeney S, Loblinzk J and Bateson D. (2026). What do people with intellectual disability think about self-collection? Easy Read Version. Social Sciences and Medicine. doi: 10.1111/hex.70920
