# Supplementary material for: The Role of Self‐Collection in Optimising Cervical Screening for People With Intellectual Disability: A Multi Perspective Qualitative Study
Source: Health Expect. 2026 Oct 25;29(5):e70920. doi: 10.1111/hex.70920 (PMC13696019; doi:10.1111/hex.70920)
Supplement: Supplementary file 2 [file HEX-29-e70920-s005.pdf]

PARTICIPANT INFORMATION STATEMENT AND CONSENT FORM

ScreenEQUAL

This sheet has information about

- a research study called **ScreenEQUAL**

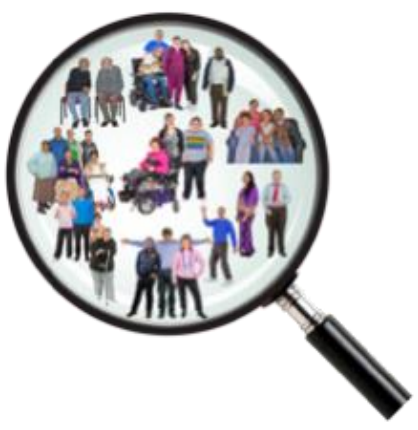

Research means

- finding out what people think about things
- using the information to help other people

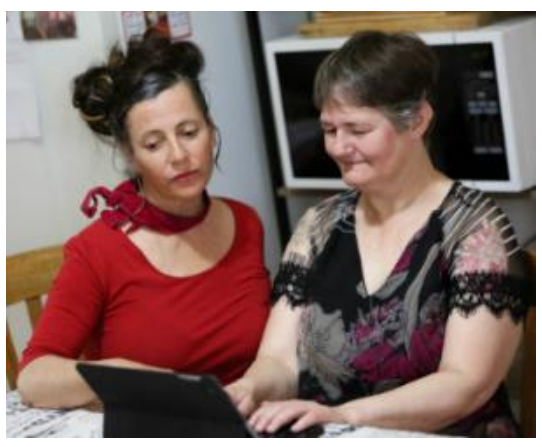

You can ask someone you know and **trust**

- to help you understand this sheet.

Who is doing the research?

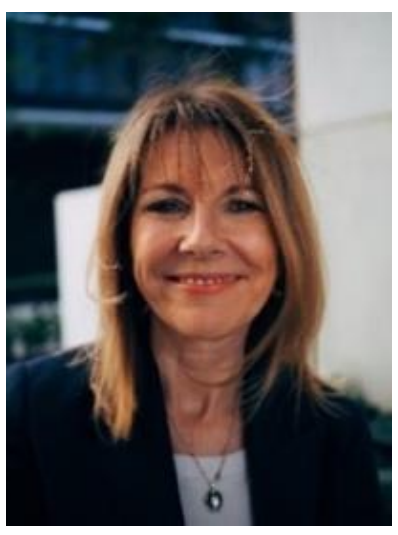

Professor Deborah Bateson

- The Daffodil Centre, Cancer Council NSW
- The University of Sydney

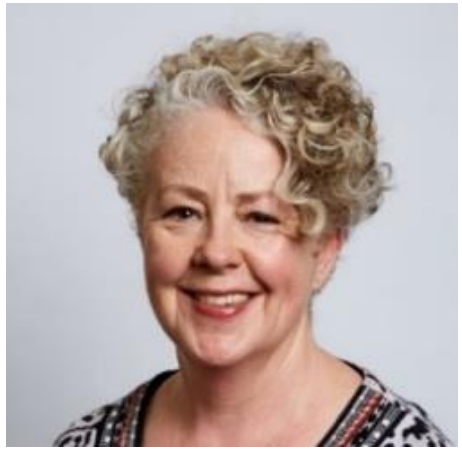

Professor Jane Ussher

- Western Sydney University

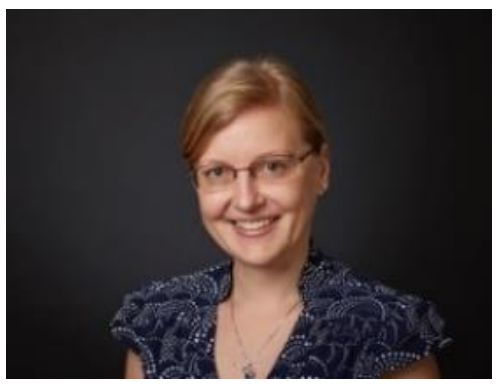

Professor Iva Strnadová

- UNSW Sydney
- Self-Advocacy Sydney

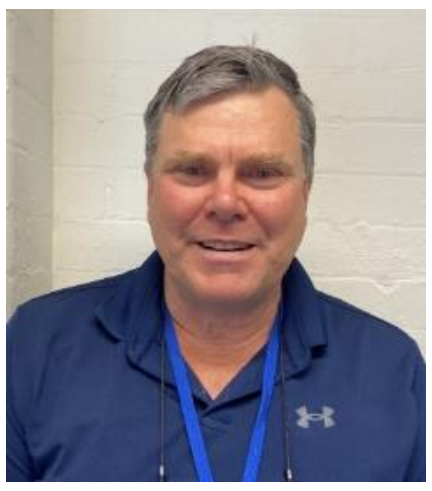

Professor Michael David

- The Daffodil Centre, Cancer Council NSW
- The University of Sydney

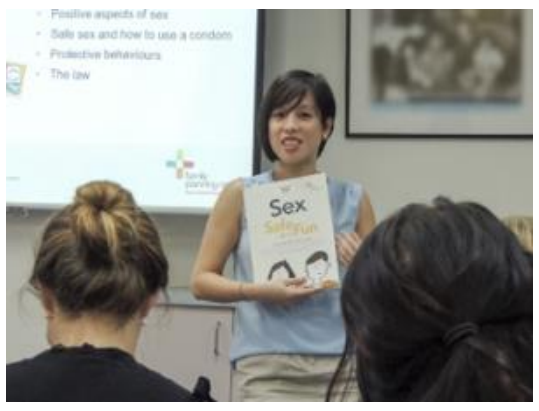

Ee-Lin Chang

- Family Planning NSW

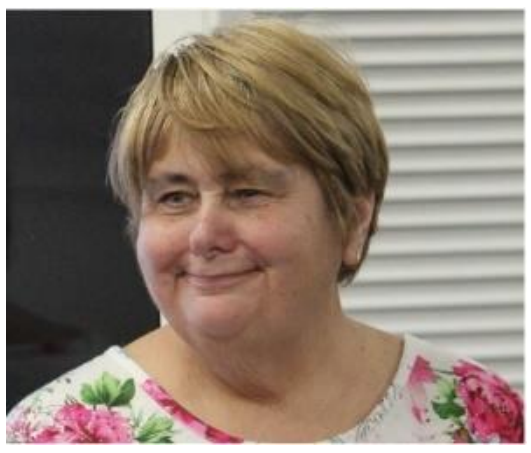

Julie Loblinzk OAM

- UNSW Sydney
- Self-Advocacy Sydney

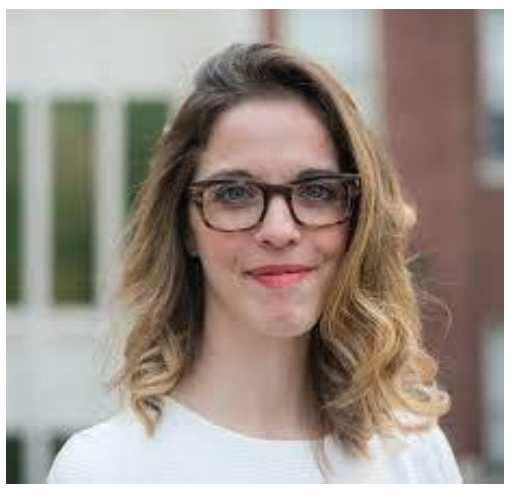

Dr Allison Carter

- Kirby Institute, UNSW Sydney

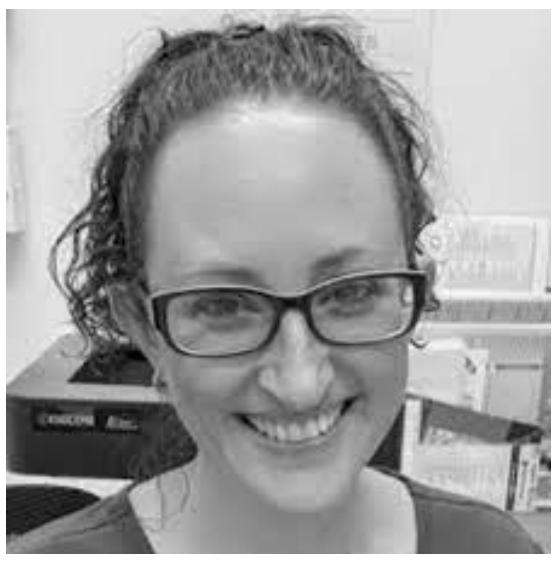

Dr Sally Sweeny

- Family Planning NSW

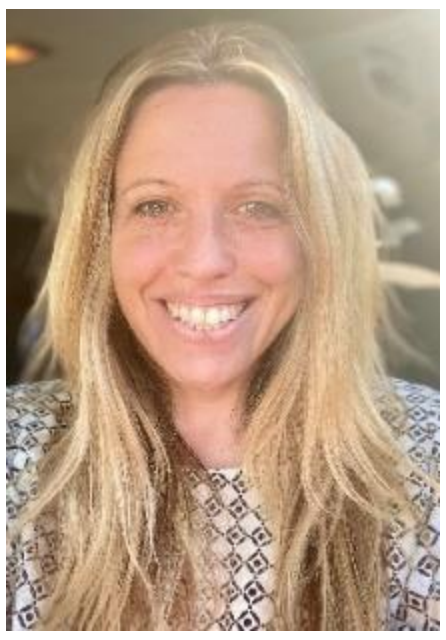

Dr Lauren Winkler

- The Daffodil Centre, Cancer Council NSW
- The University of Sydney

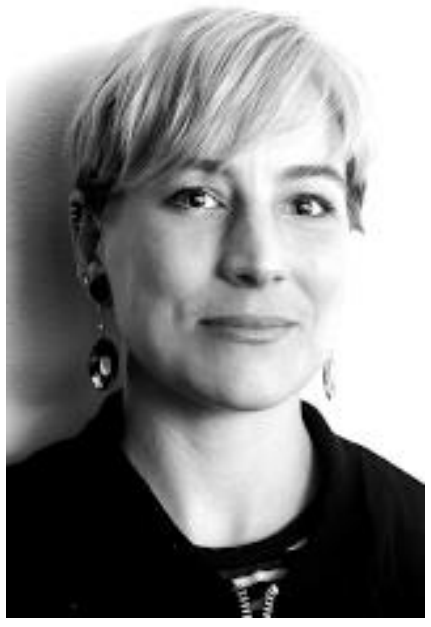

Dr Rosalie Power

- Western Sydney University

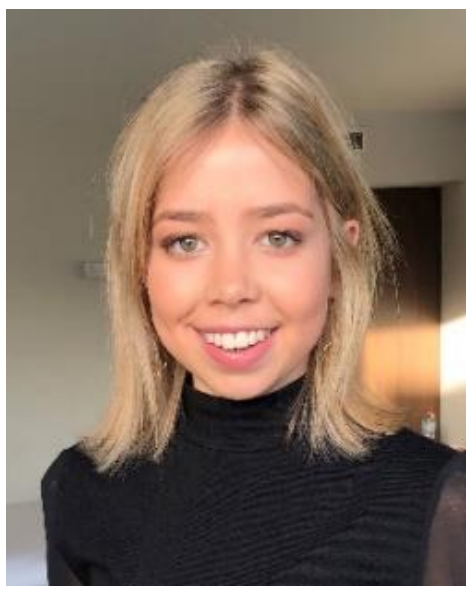

Ms Lizzie Kennedy

- The Daffodil Centre, Cancer Council NSW
- The University of Sydney

What do we want to find out?

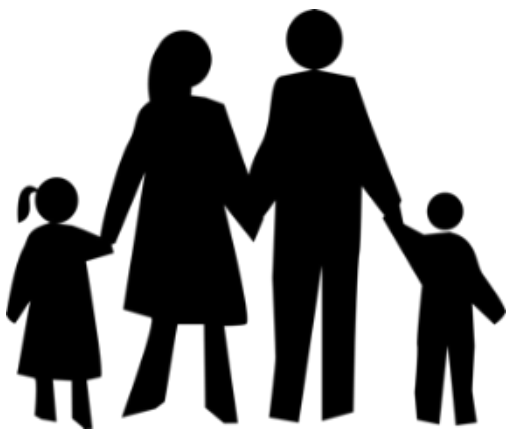

We want to learn what you think about:

- **Preventing cervical cancer**
- **Cervical Screening Tests**

Why is this study important?

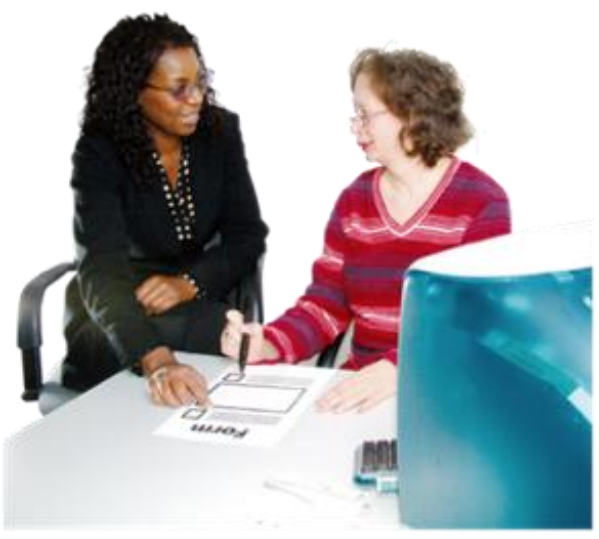

Preventing Cervical Cancer

Cervical cancer is

- one of the most stoppable cancers
- caused **by** the **human papillomavirus infection**, **HPV for short**

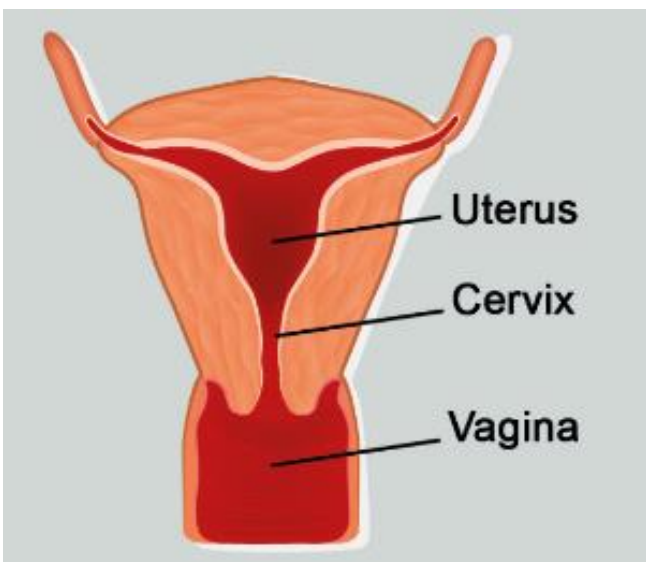

HPV can cause changes in the cervix

- that could lead to cancer

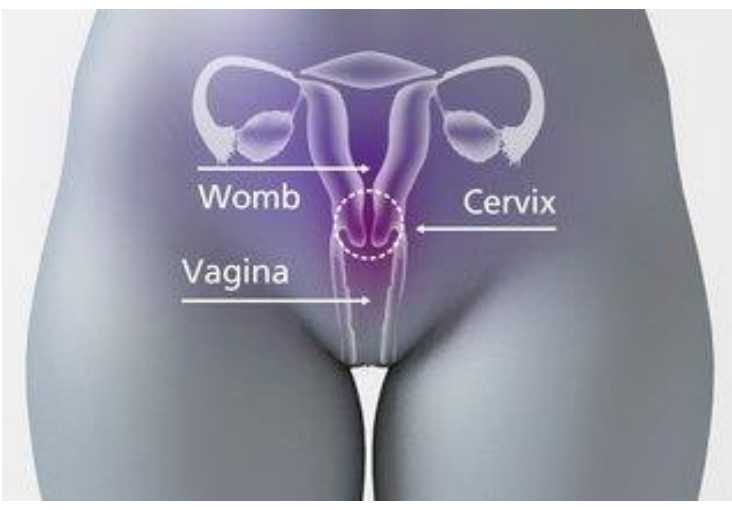

The cervix is inside your body

- at the top of your **vagina**

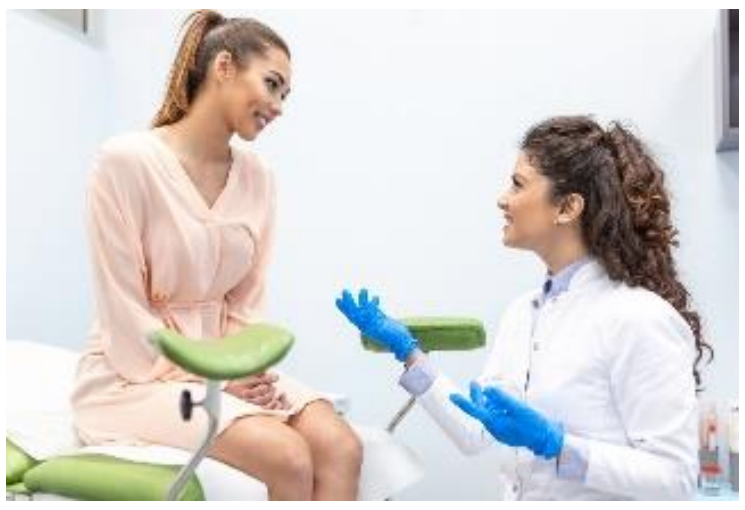

There is a test to check

- that your **cervix is healthy**

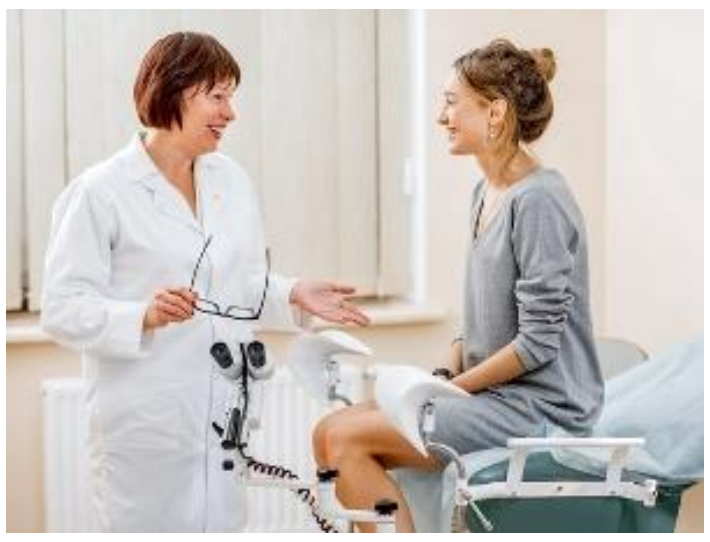

It is called a **Cervical Screening Test**

A **Cervical Screening Test**

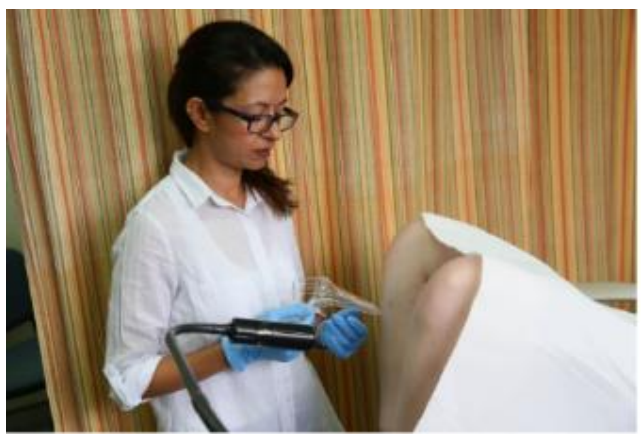

- can protect you from having cervical cancer
- is like the old 'Pap smear'
- should be done every 5 years

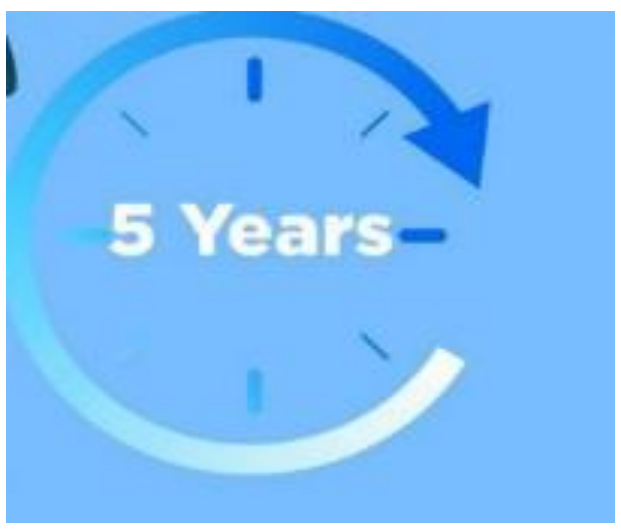

There are 2 ways to have a **Cervical Screening Test**:

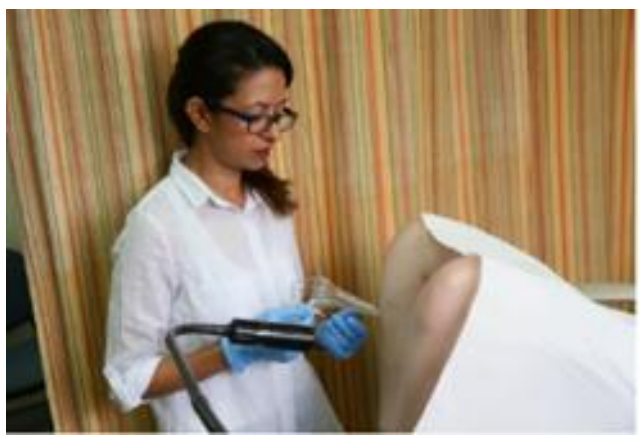

- a doctor or nurse can take the test from the **cervix** with a soft brush

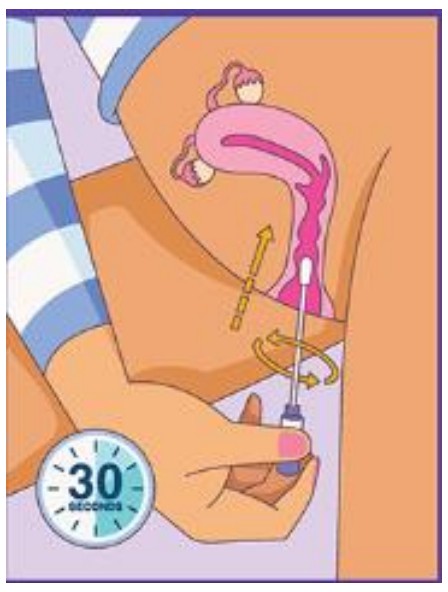

- or you can take the test yourself from the lower part of the **vagina**

- a doctor or nurse could also help you do this

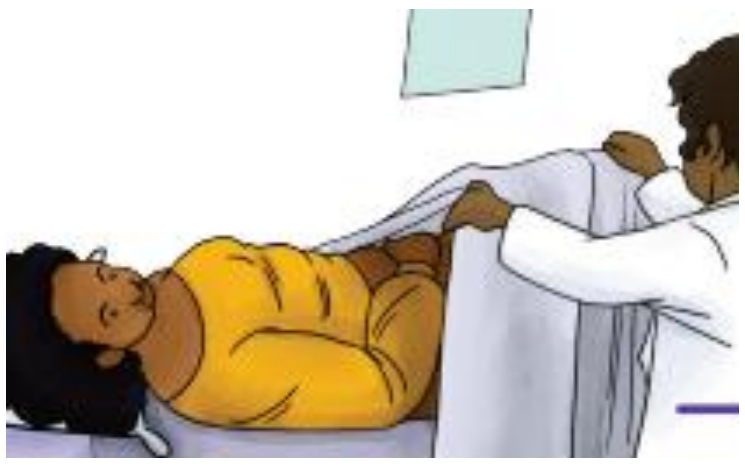

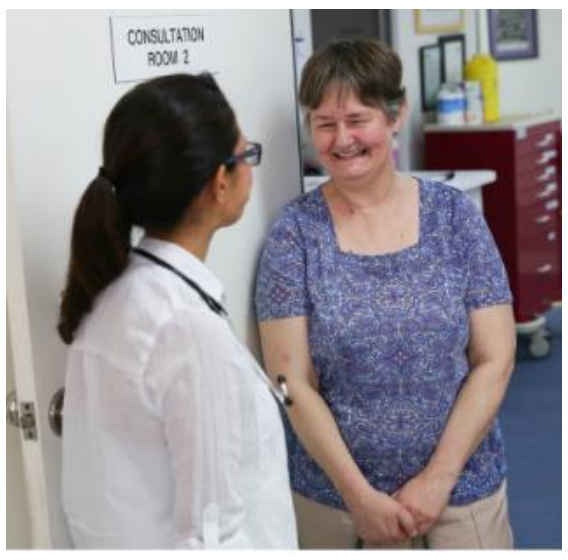

We want to learn about

- how to make it easier for people with intellectual disability
- to get a **Cervical Screening Test**

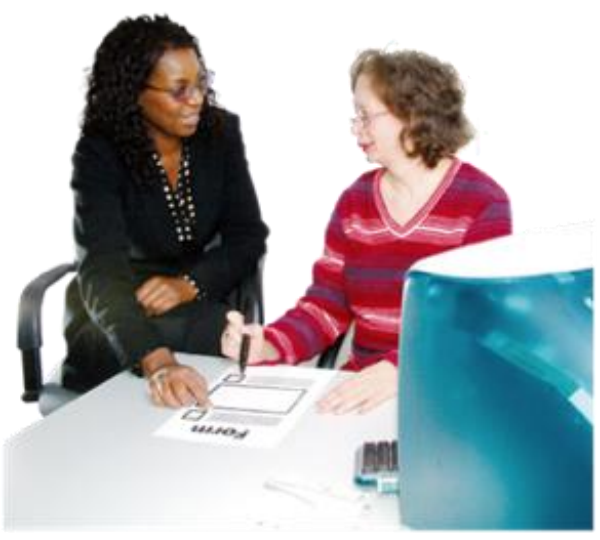

What does taking part in this study involve?

You will **talk** about cervical screening

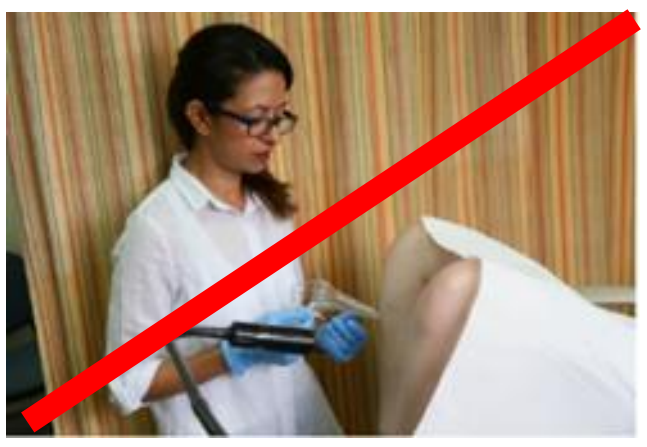

BUT

you will **not have** a cervical screening test

This meeting is **only about us talking** with you

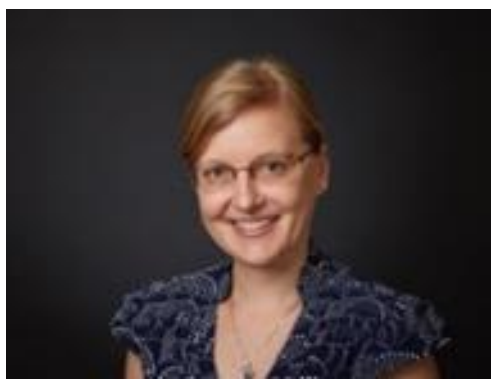

You will talk with Iva and Julie

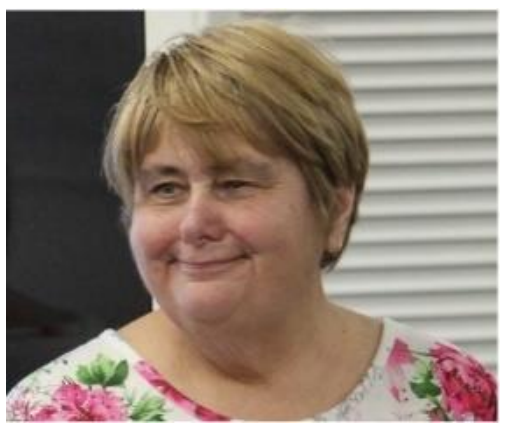

You can talk with them:

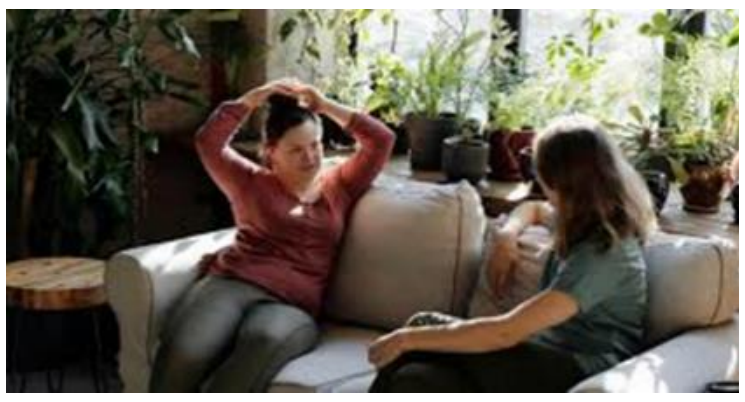

- in-person

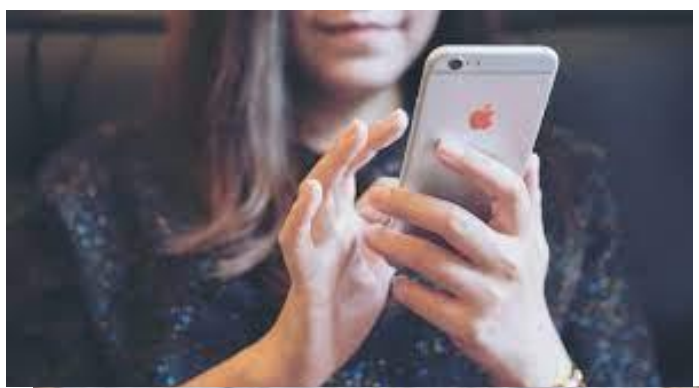

- on the phone

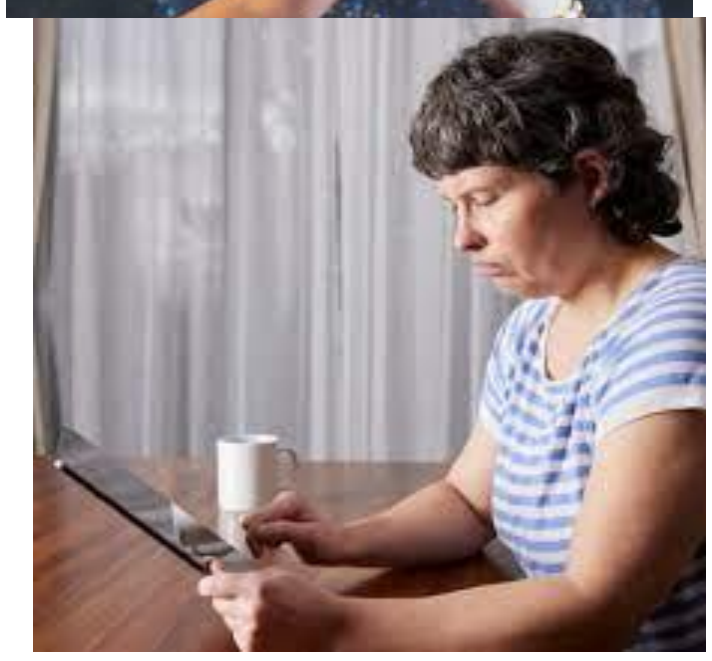

- online

Iva and Julie will ask you about:

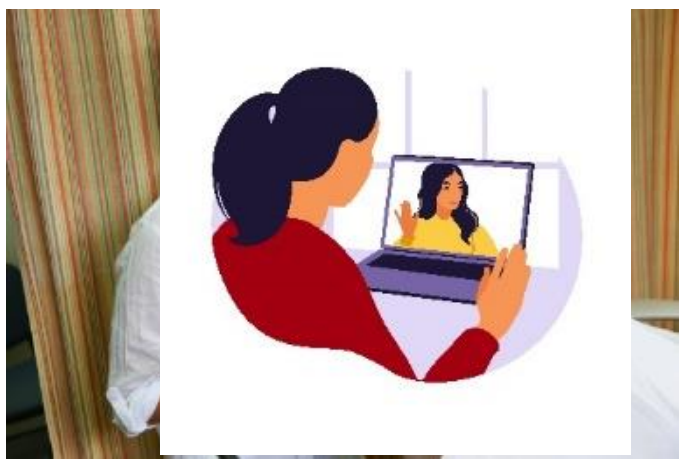

- what it was like to have Cervical Screening Test

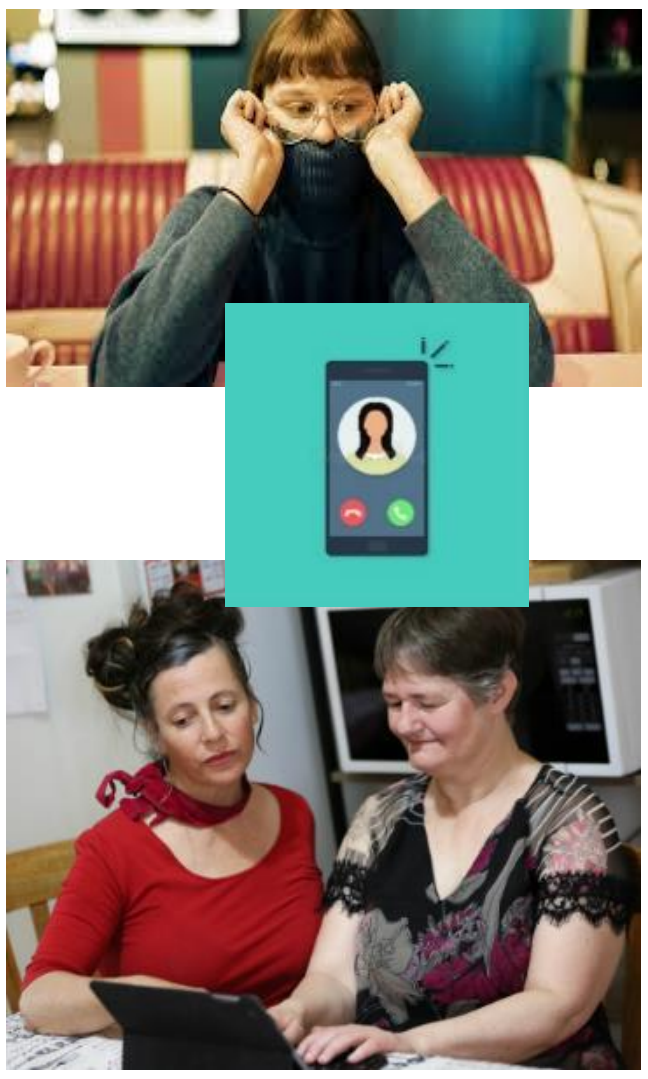

- what makes it difficult to have a Cervical Screening Test

- what makes it easier to have a Cervical Screening Test

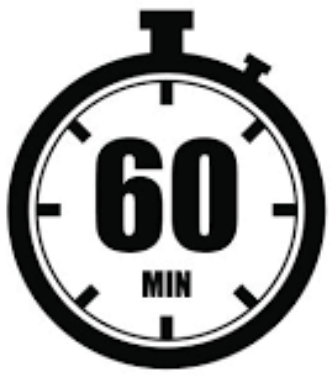

They will talk with you for about an hour

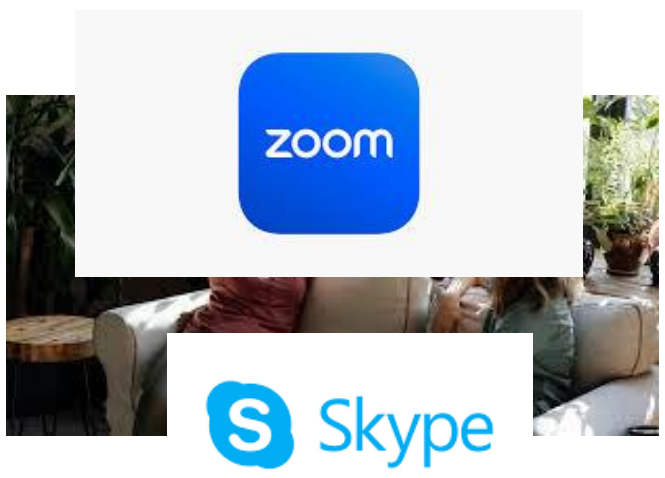

If you would like to meet in-person

we will meet you at a place you choose

If you want to talk to Iva and Julie online you can choose to talk with them on:

- Zoom

- Skype

- Facetime

- Teams

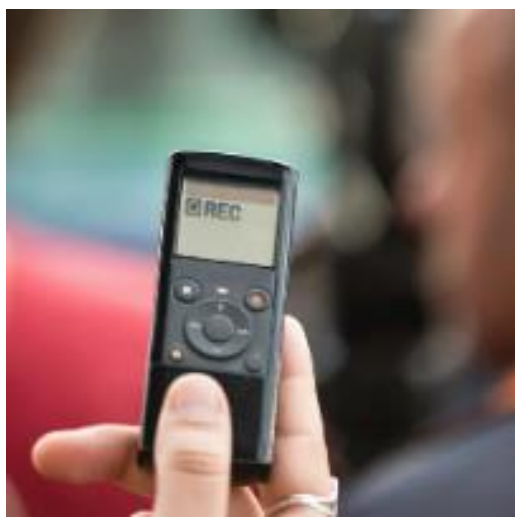

We will ask to record what you say

If you do not want us to, that is OK

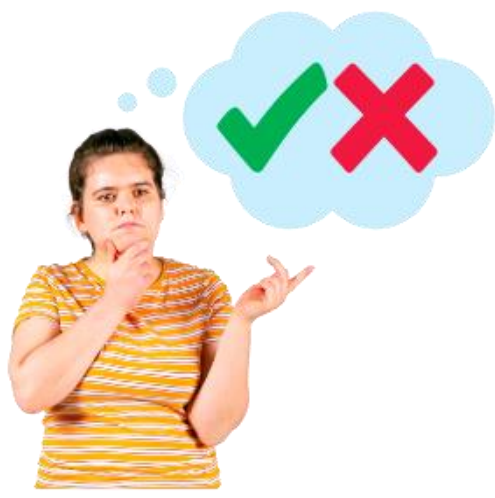

You can choose which questions to answer

You can say no if you do not want

- to answer a question

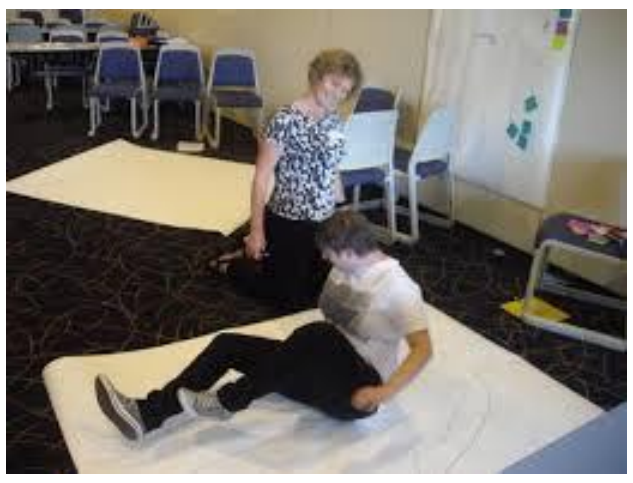

You can **also** choose to take part in:

- Body Mapping
- **or**
- Photovoice

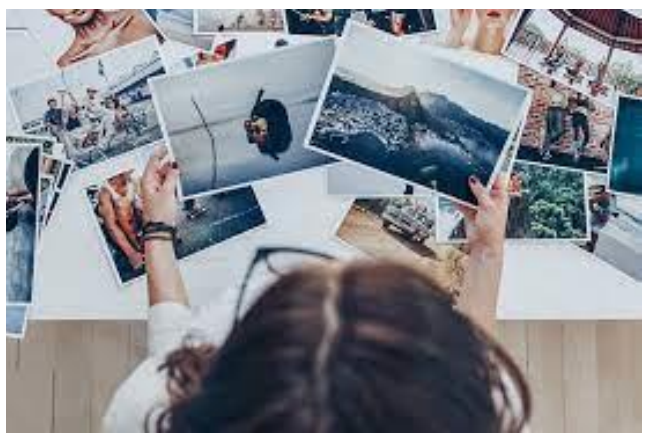

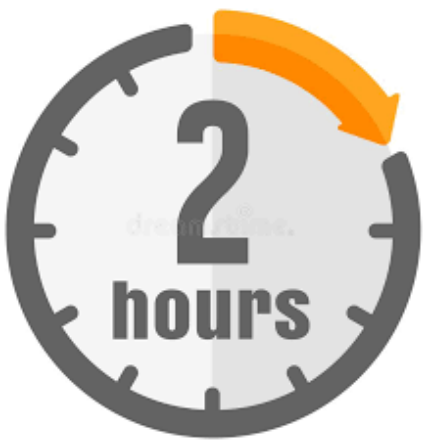

This will take about 2 hours.

We will explain these choices to you.

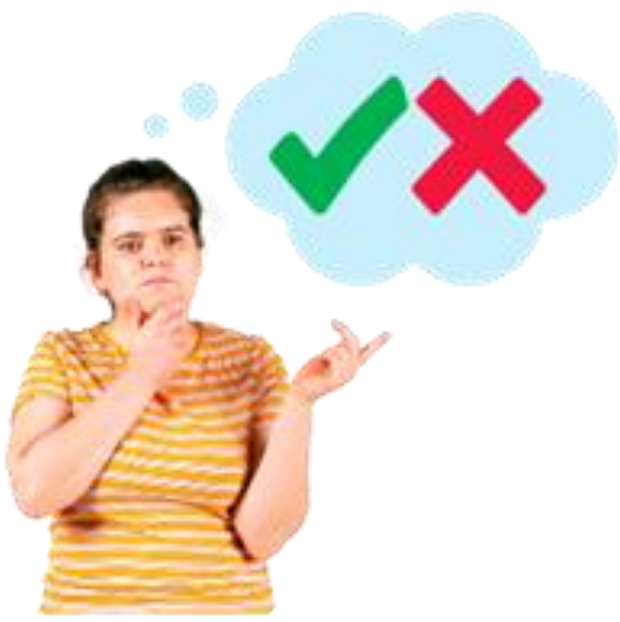

You can say no to taking part in:

- Body Mapping
- **or**
- Photovoice

Body Mapping

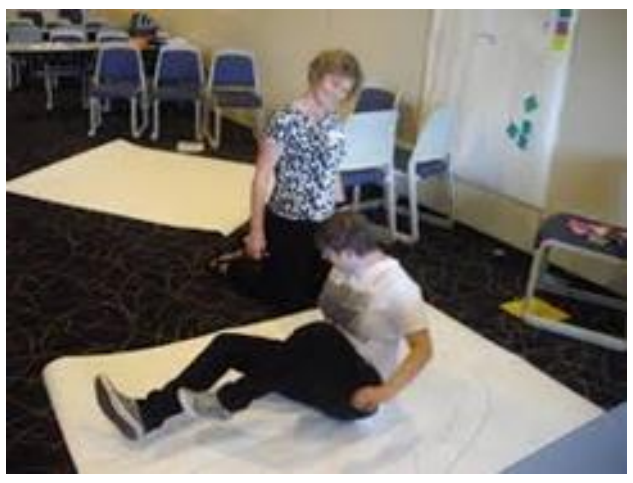

- We will draw an outline of your body.

  This body represents you.

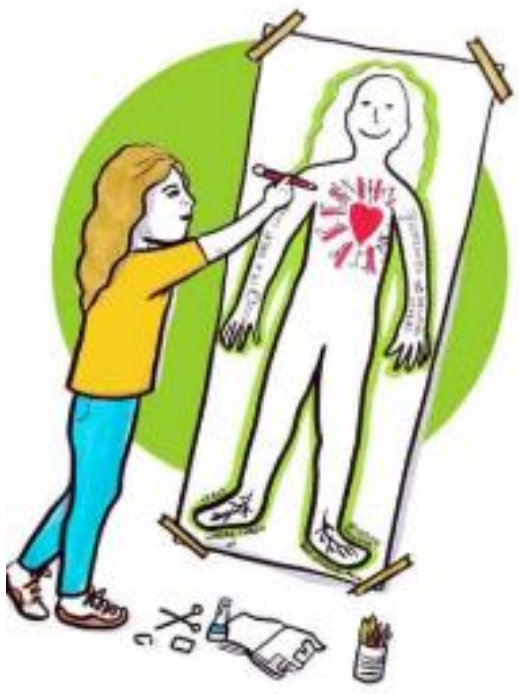

- You can draw on and around the outline about your experiences

- We will then talk to you about what you have drawn

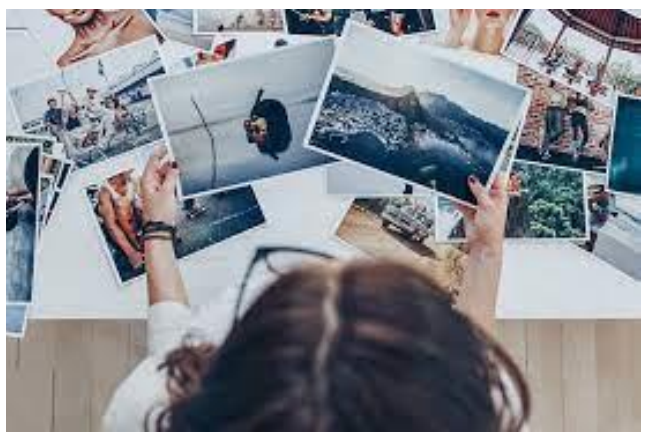

Photovoice

- You will talk to Iva and Julie about **Cervical Screening**

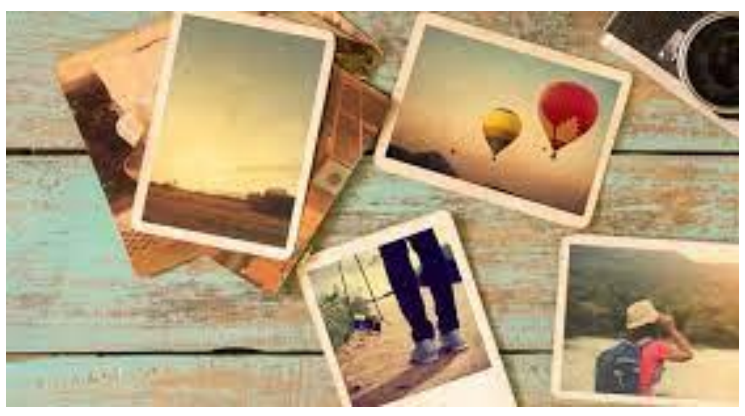

- You will choose photos that represent your feelings and experiences

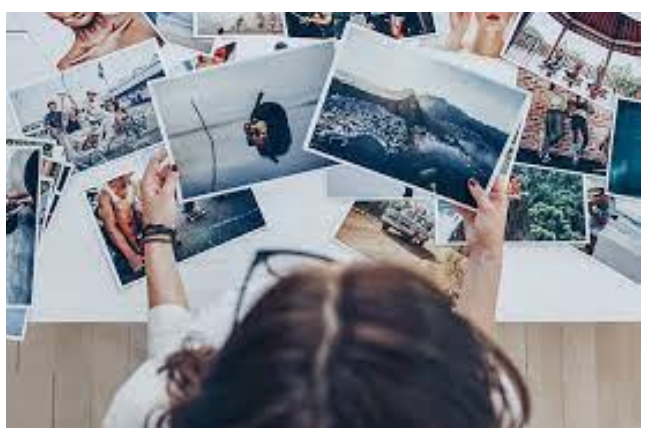

- We will talk about the photos and your experiences

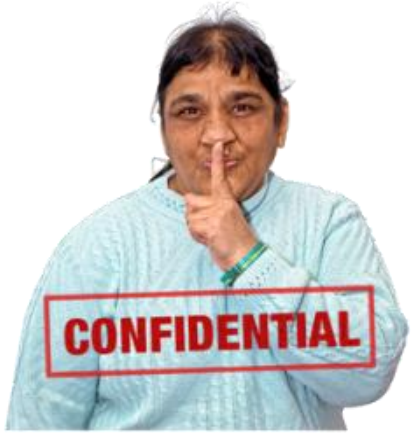

We will keep your personal information private

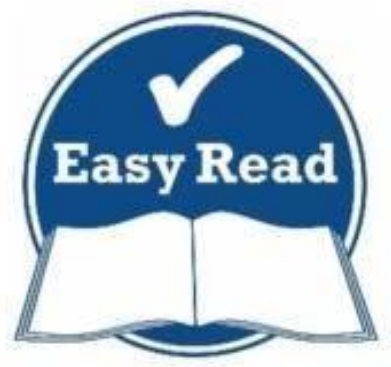

We will not tell anybody

- your name or where you live

No one will know it was you who took part

We will write about what we find

If you want, we will send you

- Easy Read summary of what we found

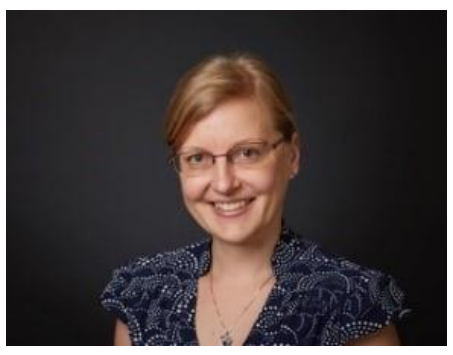

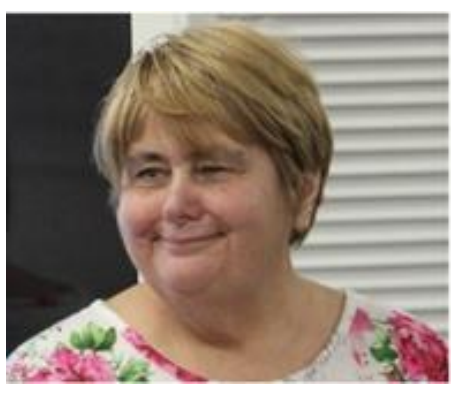

If you need help to understand

- Iva and Julie will talk with you

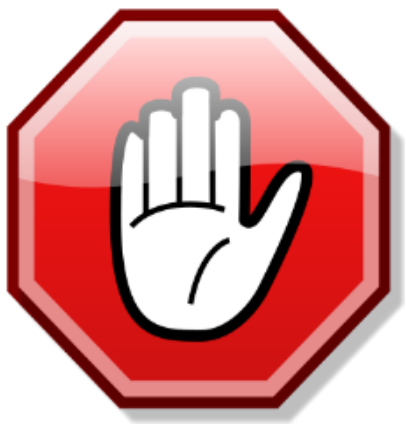

You can take part if you want to.

You can say no.

It is your choice.

No one will be angry if you say no.

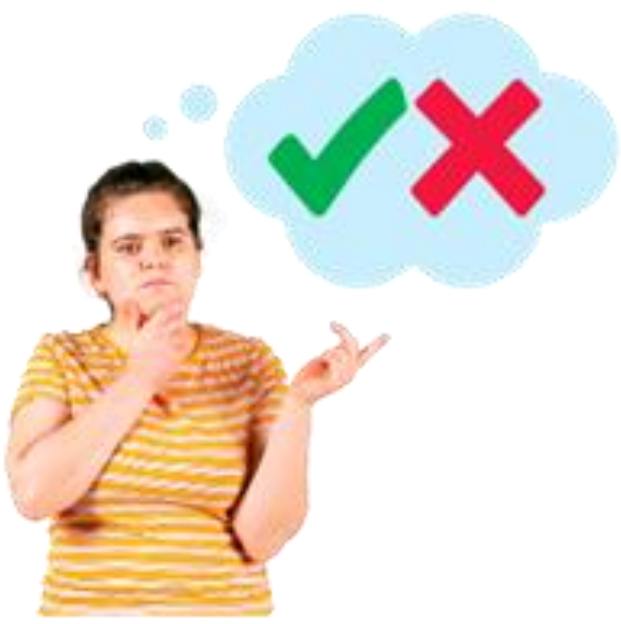

Even if you say yes

- you can change your mind later.

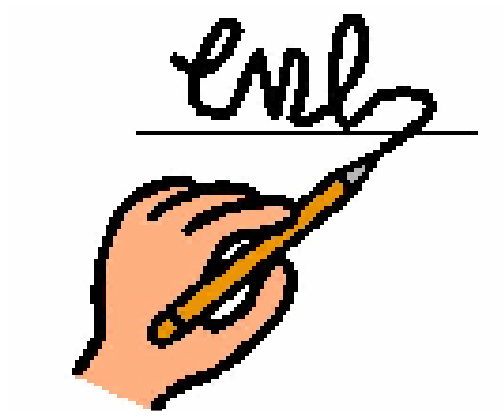

If you want to take part

- please sign the consent form.

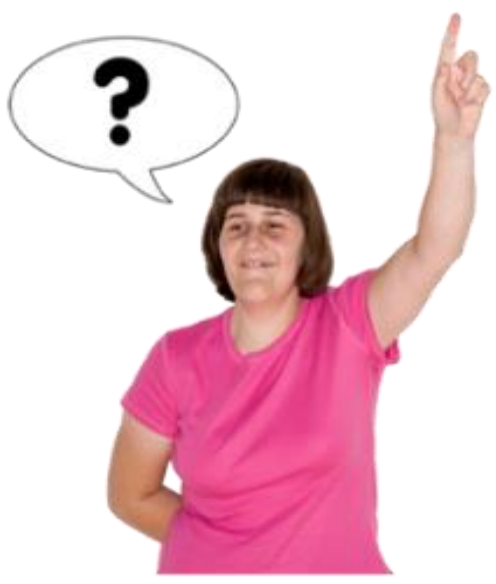

If you have **any questions**

call or email Iva and Julie

Phone: [removed for anonymity]

Email: [removed for anonymity]

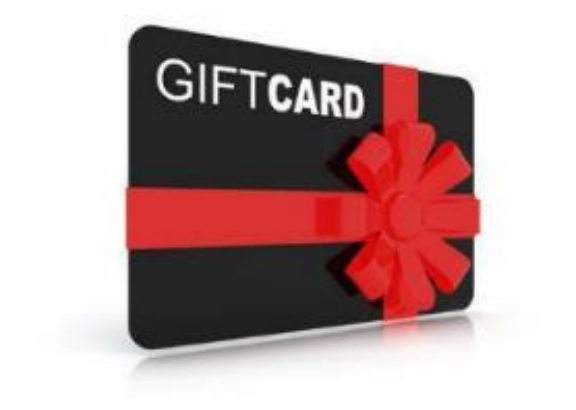

If you decide to take part

- we will give you a **[removed] gift card**.

The gift is to thank you for your time.

We will give it to you at your interview.

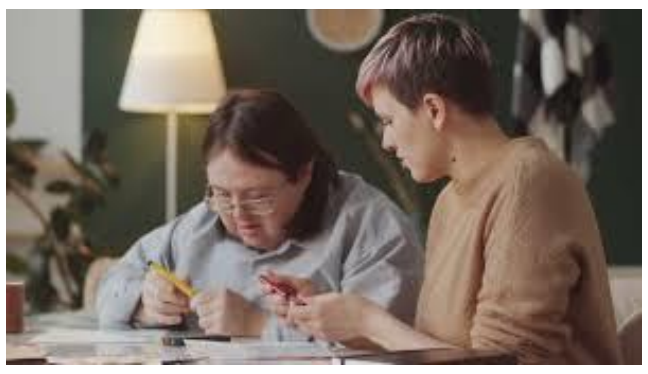

You can ask someone you know and trust to come with you.

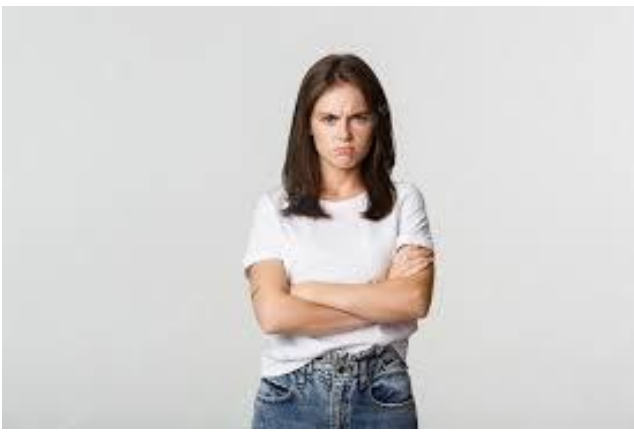

If you are you not happy with the study, you can get in touch with the lead researcher:

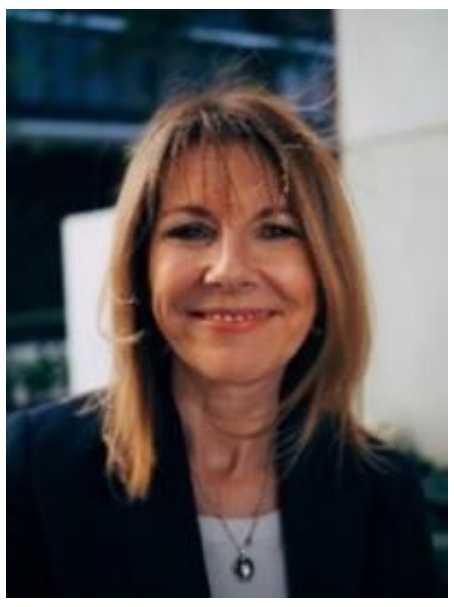

Name: Deborah Bateson

Phone: [removed for anonymity]

Email: [removed for anonymity]

Or

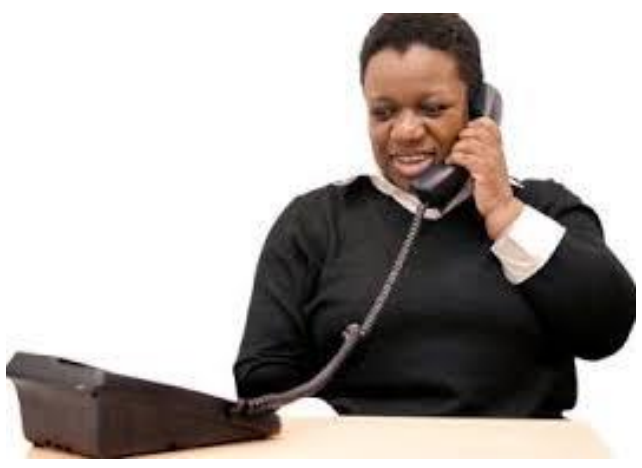

Human Ethics Manager

Phone: (02) [removed]

Email: [removed]

It is their job to listen to you and find out what happened

Tell them this number: [insert HREC number].

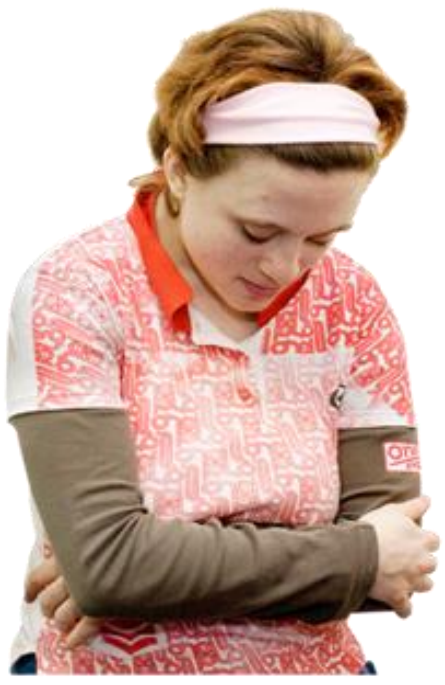

If you feel upset after taking part, talk to some who supports you or call:

- Mental Health Access Line: [removed]
- Lifeline: [removed]
- People with Disability Australia: [removed]

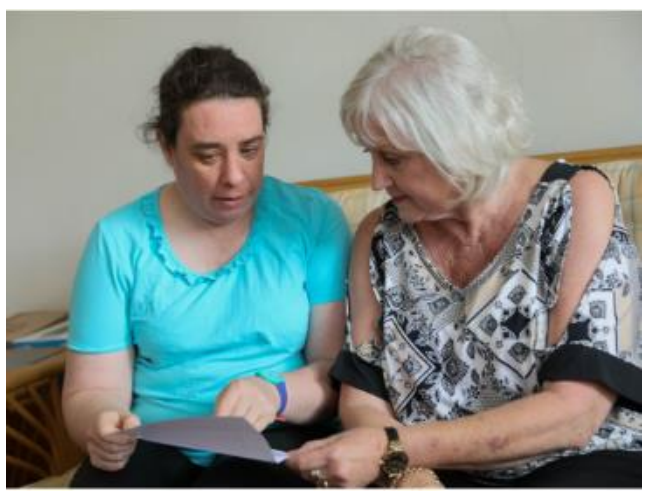

Before you sign the consent form, please check:

- I have read the sheet or had someone read it to me

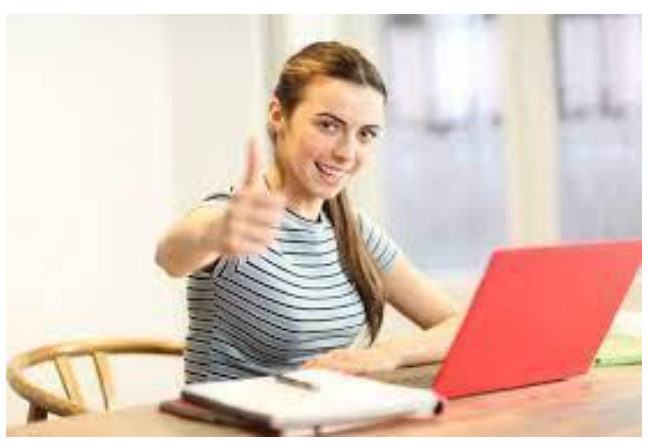

- I understand what will happen during the study
- why it is happening

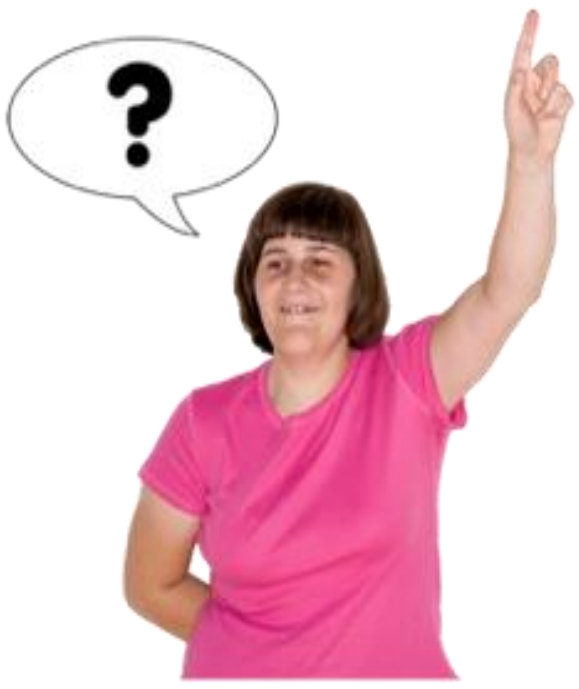

Before you sign the consent form, do you agree:

- I was allowed to ask questions about what will happen.

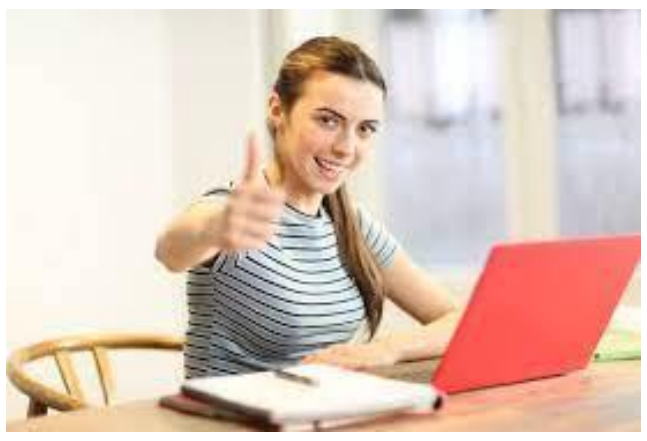

- I was happy with the answers I was given.

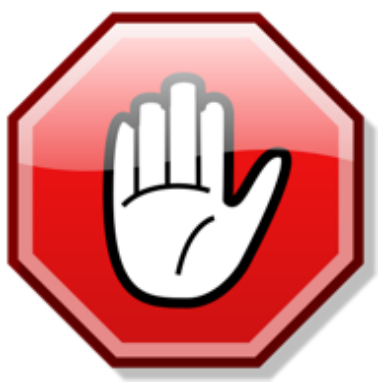

- I choose to take part in the study.
- No one is making me.

I know that I can change my mind at any time.

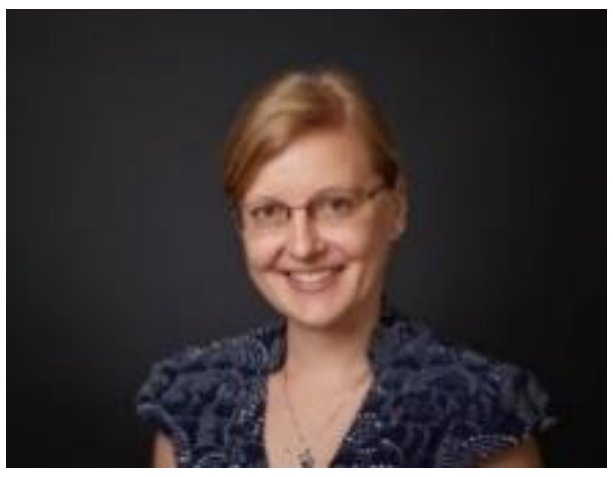

I know that Iva and Julie

will collect information about me

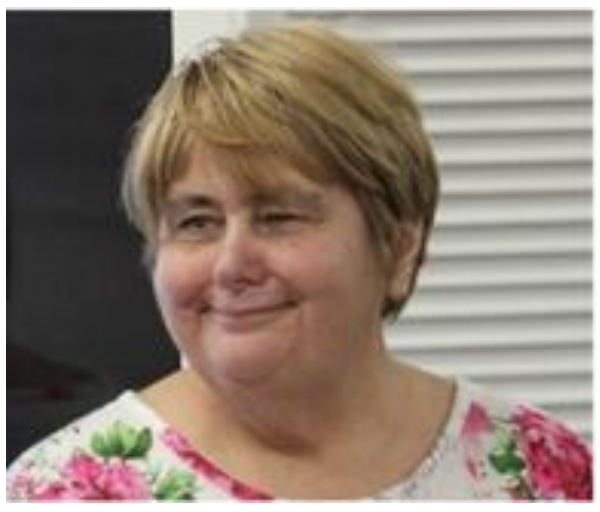

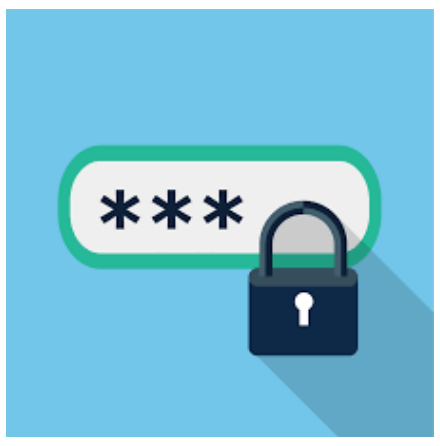

- Iva and Julie will keep my information private

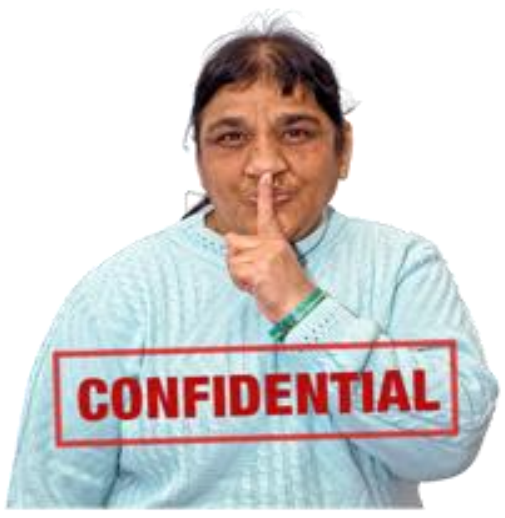

- no one will know it was me who took part

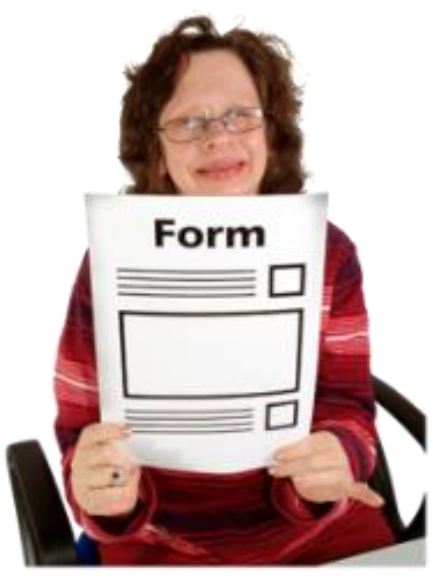

I know that I will get a copy of this form to keep

ScreenEQUAL

I WANT TO TAKE PART

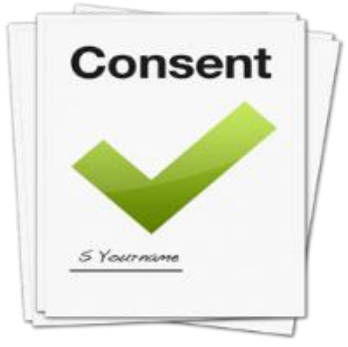

1. I WANT TO TAKE PART IN AN INTERVIEW

I am signing this form as I want to take part in an interview.

I understand what this research is about.

My signature: ____________________________________

Date: ____________________________________

2. AND I WANT TO DO BODY MAPPING OR PHOTOVOICE

My signature: ____________________________________

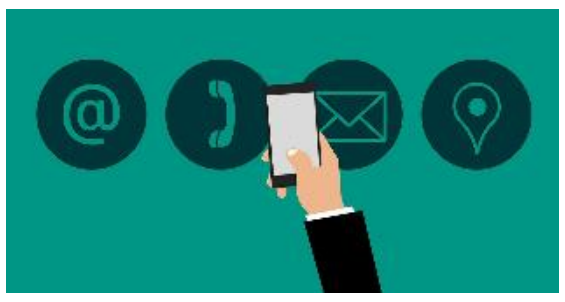

My contact details

My phone number: ____________________________________

My address: ____________________________________

My email address: ____________________________________

I understand why you need my contact details.

I know you will need to contact me to take part in research.

Witness signature: ______________________________

Date: ______________________________

A **witness** is someone who saw you sign the form.

You can email this sheet to Iva and Julie: [removed]

Or

You can bring this sheet to your interview

I DO NOT WANT TO TAKE PART ANYMORE

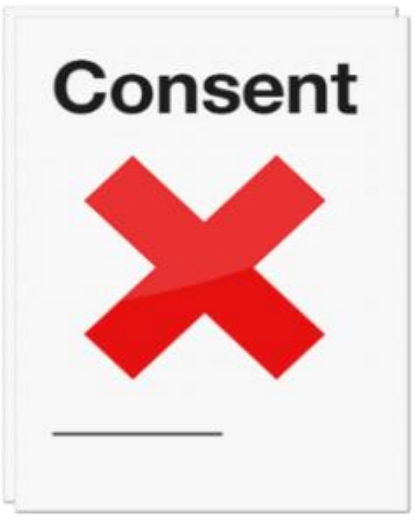

I am signing this form because I changed my mind.

I do not want to take part anymore.

My signature: ______________________

Date: ______________________

Witness signature: ______________________

Date: ______________________

A **witness** is someone who saw you sign the form.

You can email this sheet to Iva and Julie: [removed]
