# Supplementary material for: The Role of Self‐Collection in Optimising Cervical Screening for People With Intellectual Disability: A Multi Perspective Qualitative Study
Source: Health Expect. 2026 Oct 25;29(5):e70920. doi: 10.1111/hex.70920 (PMC13696019; doi:10.1111/hex.70920)
Supplement: Supplementary file 3 [file HEX-29-e70920-s001.docx]

# Supplementary Table. Composition of the Advisory Group (N=12)

Advisory group members are described by role and expertise to preserve anonymity.

| **Advisory group member role** | **Background and expertise** |
| --- | --- |
| 1. Lived Experience Expert | Team leader of a peer support and self-advocacy program at an Australian non-for-profit advocacy organization run by and for people with intellectual disabilities to empower young people aged 18-35 to speak up and represent themselves and others in their community. She also advocates for and supports parents and carers of children with disabilities. |
| 2. Lived Experience Expert | Inclusion projects officer at an Australian advocacy organisation led by people with intellectual disability and is involved in several advocacy projects to support people with intellectual disability to participate in decision-making, including their rights and choices as disability and health consumers. |
| 3. Youth and Disability Advocate with Lived Experience of Physical Disability | Youth Development and Member Engagement Officer for a national advocacy organisation for women with disabilities with expertise in social sciences, community and systematic advocacy in disability rights, gender equality, and youth representation globally. |
| 4. Adolescent and Paediatric Gynaecologist | Staff Specialist Gynaecologist at a tertiary hospital in the state of New South Wales, with extensive experience providing healthcare to adolescents and people with intellectual disability, including management of patients requiring gynaecological care. |
| 5. LGBTQIA+ Health Communications Expert | Health communications and program development specialist with expertise in inclusive health promotion, cancer prevention campaigns, and engagement with LGBTQIA+ communities. |
| 6. Community Disability Health Manager | Health service and project management professional with expertise in improving healthcare accessibility and inclusion for people with intellectual disability. |
| 7. Intellectual Disability Health Researcher and Clinician | Academic and clinical leader with extensive experience in intellectual disability health, health service innovation, advocacy, education, and applied health research. |
| 8. Cervical Screening and Public Health Expert | Public health professional with expertise in cancer screening programs, stakeholder engagement, implementation, and primary care partnerships. |
| 9. Disability Services Leader | Senior nursing and disability sector leader with extensive experience in disability support services, service reform, and supported independent living. |
| 10. Women's Health Nurse | Community health nurse with expertise in women's health, cervical screening, outreach services, and care for underserved and regional populations. |
| 11. Aboriginal And Torres Strait Islander Background Obstetrician and Gynaecologist | Obstetrician and Gynaecologist with expertise in colposcopy and in improving healthcare access and outcomes for Aboriginal and Torres Strait Islander communities. |
| 12. Researcher in intellectual disability and psychologist | Registered psychologist, researcher, and disability advocate with expertise in intellectual disability, mental health, ageing, and health outcomes for people with intellectual disability. Extensive experience in research, education, public engagement, and disability rights advocacy, including improving quality of life and health equity for people with intellectual disability. |
