# Supplementary material for: The Role of Self‐Collection in Optimising Cervical Screening for People With Intellectual Disability: A Multi Perspective Qualitative Study
Source: Health Expect. 2026 Oct 25;29(5):e70920. doi: 10.1111/hex.70920 (PMC13696019; doi:10.1111/hex.70920)
Supplement: Supplementary file 4 [file HEX-29-e70920-s002.docx]

## Supplementary Material 2: COREQ (COnsolidated criteria for REporting Qualitative research) Checklist

**Description**

Completed 32-item COREQ checklist outlining reporting of the research team, study design, data collection, analysis, and reporting procedures for this multi-perspective qualitative study.

**Manuscript:** The role of self-collection in optimising cervical screening for people with intellectual disability: A multi perspective qualitative study

**Reference:** Tong, A., Sainsbury, P., & Craig, J. (2007). Consolidated criteria for reporting qualitative research (COREQ): A 32-item checklist for interviews and focus groups. International Journal for Quality in Health Care, 19(6), 349–357.

| **Topic** | **Item No.** | **Guide Questions/Description** | **Reported on Page/Section** |
| --- | --- | --- | --- |
| Domain 1: Research Team and Reflexivity | | | |
| ***Personal Characteristics*** | | | |
| Interviewer | 1 | Which author/s conducted the interview or focus group? | Section 2.3: Data collection was conducted by the authors (LW), (RP), (IS), (JL), (CB) and (HJ). Specifically, interviews with were undertaken by:  (IS) and (JL) for people with intellectual disability; (LW) for health professionals; (CB) for family members and support people; (RP for disability sector stakeholder); and (HJ) across participant groups, as needed. |
| Credentials | 2 | What were the researcher’s credentials? e.g. PhD, MD | Section 2.3:  (LW): PhD in Women’s and Children’s Health, focusing on the design, implementation and evaluation of health interventions to improve outcomes for young cancer survivors, MClinPsych and BPsych (Hons).  (RP): PhD, University of Sydney in marginalized population health, specialising in sexual health, disability and LGBTQ health, MPH (Sexology), BAppSci (Leisure and Health).  (KA): PhD in Psychology, examining how antisocial online behaviours are rationalised and normalised within toxic and subversive online communities, MRes, BPsyc (Hons).  (AC): PhD in Public Health and Epidemiology, focusing on the social  determinants of sexual and reproductive health and rights, with a focus on important but understudied and underrepresented communities, including people with intellectual disability; BSc; MPH.  (CB): PhD in Special Education and Teaching examining literacy instruction for students with disability; Bed (Special Education)  (IS): Professor in Special Education and Disability Studies which aims to improve health equity and accessibility with, and for people with intellectual disability through inclusive research approaches that meaningfully involve people with lived experience as co-researchers; Master of Special Education; Doc Special education.  (JL): Lived experience researcher with expertise in the co-production of new knowledge to improve health outcomes for people with intellectual disabilities and a board member of a community-controlled intellectual disability advocacy organisation aimed at helping members learn to speak up for themselves, know their rights, and make their own choices, Certificate III and IV in Disabilities; Graduate Certificate in Developmental Disabilities.  (JU): Professor of Women's Health Psychology with expertise in qualitative research methodologies and its application in understanding gender issues in cancer and cancer care, BA; Dip (Psych).  (SS):  (SS): BSc. (Med)MBBS FRACGP and clinician researcher in the fields of general practice, sexual & reproductive medicine.  (DB): Professor of Practice focusing on translational and implementation aspects of cervical cancer control and elimination, in Australia and globally; MA (Oxon); MSc (LSTM); MBBS. |
| Occupation | 3 | What was their occupation at the time of the study? | Section 2.3: Academic researcher (LW), (RP), (KA), (IS), (AC), (JL), (DB), (CB); Senior Health Promotion Officer and Co-Researcher (HJ); Clinician researcher (SS) |
| Gender | 4 | Was the researcher male or female? | Section 2.4: The interviewers were female. The project team included a male CI, and female members, including cisgender and queer-identifying members. First author (LW) and co-researchers (RP), (KA), (AC), (HJ) and (CB) undertook interviews with healthcare professionals, families and support people and disability service providers. Co-author (IS) undertook interviews with people with intellectual disability. |
| Experience and training | 5 | What experience or training did the researcher have? | This project brings together clinicians, researchers and lived experience researchers, with experience in  sexual and reproductive health, disability studies, and cervical cancer screening. Section 2.3: (LW), (RP), (KA), (AC) and (CB) are early-to-mid career researchers who have 5 or more years of experience in qualitative research in various fields including cancer, special education, equity and health systems ; (IS) and (JL) have over two decades of experience conducting inclusive research with people with intellectual disability including the delivery of >20 inclusive research studies over 12 years; has worked in trauma-informed practice and co-facilitate trauma-informed training; (DB)  has worked as a clinician, researcher and educator in reproductive and sexual health for > 20 years and clinical expertise as a member of the National Cervical Screening Program Quality and Safety  Monitoring Committee (QSMC) and Clinical Expert Panel;(SW) is a specialist General Practitioner and clinician researcher and Chief Investigator on several competitive research grants involving HPV screening and vaccination. |
| ***Relationship with Participants*** | | | |
| Relationship established | 6 | Was a relationship established prior to study commencement? | Section 2.2: Participants with intellectual disability were recruited through established disability organisations; no prior relationships with individual participants. |
| Participant knowledge of the interviewer | 7 | What did the participants know about the researcher? | Section 2.2: Participants with intellectual disability received Easy Read participant information sheet explaining the research and research team. Other participant groups received Lay English participant information sheet explaining the research and what participation involved. |
| Interviewer characteristics | 8 | What characteristics were reported about the interviewer/facilitator? | Section 2.4: Interviewers comprised women researchers, with and without disability, cisgender and queer-identifying members, researchers from different cultural backgrounds; reflexive practices described. |
| Domain 2: Study Design | | | |
| ***Theoretical Framework*** | | | |
| Methodological orientation and theory | 9 | What methodological orientation was stated to underpin the study? | Section 2.1: Inclusive co-production framework; trauma-informed approach. Section 2.4: Reflexive thematic analysis (Braun & Clarke, 2022). |
| ***Participant Selection*** | | | |
| Sampling | 10 | How were participants selected? | Section 2.2: Purposive sampling through professional networks, peak bodies, disability organisations; snowball sampling. |
| Method of approach | 11 | How were participants approached? | Section 2.2: People with intellectual disability were approached through grassroots disability advocacy organisations using Easy Read materials; partner organisations shared social media advertisements. Other participant groups were approached via community-controlled organisations and peak bodies for carers and healthcare professionals; partner organisations shared social media advertisements. |
| Sample size | 12 | How many participants were in the study? | Section 3: People with intellectual disability (9), families and support people (12), healthcare professionals (19) and disability service providers (19). |
| Non-participation | 13 | How many people refused to participate or dropped out? Reasons? | Section 2.2: Two online interviews with people with intellectual disability were excluded due to fraudulent participation; number of refusals not reported. |
| *Setting* | | | |
| Setting of data collection | 14 | Where was the data collected? | Section 2.3.1: All interviews were conducted in person, in Sydney Australia. |
| Presence of non-participants | 15 | Was anyone else present besides the participants and researchers? | One participant with intellectual disability was accompanied by their mother as a support person, at the participant's request. This was consistent with the study's person-centered approach, which offered participants choice about support during interviews (Section 2.1). |
| Description of sample | 16 | What are the important characteristics of the sample? | Section 3 and Table 1: People with intellectual disability. Age range 26-65 (mean 42); 9 cis-women, 1 non-binary; 7 with screening experience, 2 never screened; demographic details in Table 1. |
| ***Data Collection*** | | | |
| Interview guide | 17 | Were questions, prompts, guides provided by the authors? Was it pilot tested? | Section 2.3.1: Semi-structured schedule used.  Section 2.3.2: Body mapping prompts described in separate publication referenced in-text. |
| Repeat interviews | 18 | Were repeat interviews carried out? If yes, how many? | Not applicable; single interviews conducted. The first interview served as a pilot test, but the data was included as no significant changes to the interview schedule were made. |
| Audio/visual recording | 19 | Did the research use audio or visual recording to collect the data? | Section 2.3: Interviews and body mapping sessions were audio-recorded. |
| Field notes | 20 | Were field notes made during and/or after the interview or focus group? | Field notes were taken either before or after the interview and included demographic information. |
| Duration | 21 | What was the duration of the interviews or focus group? | Section 2.3: Interviews lasted 17-99 minutes (); body mapping sessions approximately 60 minutes followed by brief interviews (5-15 minutes). |
| Data saturation | 22 | Was data saturation discussed? | Section 2.2: Sample size guided were by information power (Malterud et al., 2016) rather than data saturation, as data saturation is not conceptually consistent with reflexive thematic analysis (Braun & Clarke, 2021). Justification provided based on study aim, sample specificity, dialogue quality, data collection methods, and analysis strategy. |
| Transcripts returned | 23 | Were transcripts returned to participants for comment and/or correction? | No. Returning written transcripts is not accessible for people with intellectual disability. Alternative strategies to ensure trustworthiness included: co-facilitation with a researcher with intellectual disability who provided insider perspective during data collection and analysis; continuous consent processes with verbal checking during interviews; and guidance from an Advisory Group that included people with intellectual disability. To ensure equitable research methods, written transcripts were not returned to across participant groups |
| Domain 3: Analysis and Findings | | | |
| ***Data Analysis*** | | | |
| Number of data coders | 24 | How many data coders coded the data? | Section 2.4 The initial coding to develop the coding frame was conducted by four team members. Coding of the data pertaining to people with intellectual disability was conducted by the first, second, seventh and eight author and verified by the fourth and sixth authors. |
| Description of the coding tree | 25 | Did authors provide a description of the coding tree? | Section 2.4: Eight initial categories described; four final themes; Supplementary Material 3 provides detailed analytical process. |
| Derivation of themes | 26 | Were themes identified in advance or derived from the data? | Section 2.4: Themes derived from data through iterative inductive analysis. |
| Software | 27 | What software, if applicable, was used to manage the data? | Section 2.4: NVivo Version 14. |
| Participant checking | 28 | Did participants provide feedback on the findings? | Findings were not returned to individual participants, as they preferred only to receive the final Easy Read report with the findings. However, the research was guided by an Advisory Group that included people with intellectual disability who provided input throughout the study (Section 2.1).  Additionally, a researcher with intellectual disability was involved in data analysis and theme development, providing insider perspective on interpretation of findings (Section 2.4). |
| ***Reporting*** | | | |
| Quotations presented | 29 | Were participant quotations presented to illustrate the themes/findings? Was each quotation identified? | Section 3: Quotations presented throughout with participant pseudonyms. |
| Data and findings consistent | 30 | Was there consistency between the data presented and the findings? | Yes. Section 3: Quotations support theme interpretations. |
| Clarity of major themes | 31 | Were major themes clearly presented in the findings? | Yes. Section 3 and Figure 1: Four themes clearly presented with subthemes. |
| Clarity of minor themes | 32 | Is there a description of diverse cases or discussion of minor themes? | Section 3: Variation within themes discussed (e.g., positive vs. negative healthcare experiences; different views on self-collection). |
