# Supplementary material for: The Role of Self‐Collection in Optimising Cervical Screening for People With Intellectual Disability: A Multi Perspective Qualitative Study
Source: Health Expect. 2026 Oct 25;29(5):e70920. doi: 10.1111/hex.70920 (PMC13696019; doi:10.1111/hex.70920)
Supplement: Supplementary file 5 [file HEX-29-e70920-s003.docx]

Supplementary File S1
*Interview Schedule Questions and Prompts for Participants with Intellectual Disability*

Note. Interview schedules were used flexibly, with probing and follow-up questions used where appropriate.

**Description**

The semi-structured interview schedule used to explore the knowledge, experiences, preferences, barriers, and facilitators related to cervical screening and HPV self-collection among people with intellectual disability.

# People with Intellectual Disability

## Rapport-building

- What is it like seeing your GP?
- Prompt: What are the things you like and/or do not like about seeing your GP?

## Knowledge and awareness

- What do you know about cervical screening?
- Prompt: Imagine you are explaining cervical screening to a friend. What would you tell them?
- Why is it important to go for cervical screening?
- Prompt: Why would you recommend it to others?

## Self-collection

- Did you know that you can do the test yourself?
- [Explanation]: Self-collection involves taking a swab from the vagina yourself.

## Screening preferences

- If you had a choice, would you prefer a doctor to perform the test, a female doctor to perform the test, or to do it yourself?
- Prompt: Why would you prefer [insert choice]?

## Access enablers and barriers

Are the any advantages that you can think of? Whether it's better that the doctor does it, or nurse, or doing it by yourself?

## Experience with cervical screening (if any)

- Have you ever had a cervical screening test?
- Prompt: [If no] Why did you decide not to have a cervical screening test?
- Prompt: [If yes]: What were your experiences?
- Prompt: What was the doctor and/or nurse like?
- Prompt: What was the room like?
- Prompt: How was the screening process explained?
- What materials were used to explain the process? (e.g., Easy Read materials, pictures)
- Did you attend alone or with a support person? Why?

Prompt: Who was your support person?

Prompt: Were they family members? Or paid support worker?

Prompt: Did you choose the support person?

- What made cervical screening easier?
- What made cervical screening difficult?

## Support and Resources

- What can family members, guardians, or disability support workers do to make cervical screening a positive experience?
- What can doctors’ and nurses do to make cervical screening a positive experience?
- What support or resources should be offered?
